# HSV-1 Infection Induces a Downstream Shift of Promoter-Proximal Pausing for Host Genes

**DOI:** 10.1128/jvi.00381-23

**Published:** 2023-04-24

**Authors:** Elena Weiß, Thomas Hennig, Pilar Graßl, Lara Djakovic, Adam W. Whisnant, Christopher S. Jürges, Franziska Koller, Michael Kluge, Florian Erhard, Lars Dölken, Caroline C. Friedel

**Affiliations:** a Institute of Informatics, Ludwig-Maximilians-Universität München, Munich, Germany; b Institute for Virology and Immunobiology, Julius-Maximilians-University Würzburg, Würzburg, Germany; c Helmholtz Institute for RNA-based Infection Research (HIRI), Helmholtz-Center for Infection Research (HZI), Würzburg, Germany; University of Virginia

**Keywords:** HSV-1 infection, RNA polymerase II pausing

## Abstract

Herpes simplex virus 1 (HSV-1) infection exerts a profound shutoff of host gene expression at multiple levels. Recently, HSV-1 infection was reported to also impact promoter-proximal RNA polymerase II (Pol II) pausing, a key step in the eukaryotic transcription cycle, with decreased and increased Pol II pausing observed for activated and repressed genes, respectively. Here, we demonstrate that HSV-1 infection induces more complex alterations in promoter-proximal pausing than previously suspected for the vast majority of cellular genes. While pausing is generally retained, it is shifted to more downstream and less well-positioned sites for most host genes. The downstream shift of Pol II pausing was established between 1.5 and 3 h of infection, remained stable until at least 6 hours postinfection, and was observed in the absence of ICP22. The shift in Pol II pausing does not result from alternative *de novo* transcription initiation at downstream sites or read-in transcription originating from disruption of transcription termination of upstream genes. The use of downstream secondary pause sites associated with +1 nucleosomes was previously observed upon negative elongation factor (NELF) depletion. However, downstream shifts of Pol II pausing in HSV-1 infection were much more pronounced than observed upon NELF depletion. Thus, our study reveals a novel aspect in which HSV-1 infection fundamentally reshapes host transcriptional processes, providing new insights into the regulation of promoter-proximal Pol II pausing in eukaryotic cells.

**IMPORTANCE** This study provides a genome-wide analysis of changes in promoter-proximal polymerase II (Pol II) pausing on host genes induced by HSV-1 infection. It shows that standard measures of pausing, i.e., pausing indices, do not properly capture the complex and unsuspected alterations in Pol II pausing occurring in HSV-1 infection. Instead of a reduction of pausing with increased elongation, as suggested by pausing index analysis, HSV-1 infection leads to a shift of pausing to downstream and less well-positioned sites than in uninfected cells for the majority of host genes. Thus, HSV-1 infection fundamentally reshapes a key regulatory step at the beginning of the host transcriptional cycle on a genome-wide scale.

## INTRODUCTION

Lytic herpes simplex virus 1 (HSV-1) infection exerts a profound shutoff of host gene expression. Two major contributors to this shutoff are the degradation of host and viral mRNAs by the virus host shutoff protein (*vhs*) (1, 2) and a general inhibition of the host transcriptional activity by HSV-1 (3–6). Efficient recruitment of RNA polymerase II (Pol II) and elongation factors from the host chromatin to replicating viral genomes leads to a substantial loss of Pol II occupancy from the host genome as early as 2 to 3 h postinfection (h p.i.) (3–6). By 8 h p.i., host transcriptional activity is estimated to be only 10 to 20% of uninfected cells (7). We previously showed that HSV-1 infection disrupts transcription termination for the majority but not all cellular genes, leading to read-through transcription for tens of thousands of nucleotides beyond the poly(A) site (8). More recently, Rivas et al. (9) and Birkenheuer et al. (10) demonstrated that HSV-1 also impacts promoter-proximal pausing of Pol II on host genes. Following transcription initiation, Pol II pauses 20 to 60 nucleotides (nt) downstream of the transcription start site (TSS) (11, 12) as a consequence of one or more structural rearrangements within the transcription elongation complex (13). Pausing makes Pol II vulnerable to nucleosome-induced arrest, backtracking of the elongation complex along the DNA, and promoter-proximal premature termination (13). The elongation factor TFIIS can rescue Pol II from pausing and restart transcription by mediating cleavage of backtracked RNA (14). In contrast, 5,6-dichloro-1-beta-d-ribofuranosylbenzimidazole sensitivity-inducing factor (DSIF) and negative elongation factor (NELF) stabilize paused Pol II (15, 16). Phosphorylation of DSIF, NELF, and the Pol II C-terminal domain by the CDK9 subunit of the positive transcription elongation factor b (P-TEFb) is required for the release of paused Pol II into gene bodies and the switch to productive elongation (17–19). As a consequence, inhibition of P-TEFb by CDK9 inhibitors increases promoter-proximal pausing (20). While the facilitates chromatin transcription (FACT) histone chaperone complex has previously been reported to cooperate with P-TEFb to overcome NELF/DSIF-mediated inhibition of Pol II elongation (21), recent studies in *Drosophila* instead suggest a role of FACT in the maintenance of Pol II pausing, with FACT knockdown decreasing Pol II pausing (22).

The HSV-1 immediate early protein ICP22 inhibits Pol II transcription elongation by direct interaction with CDK9 (23), and ectopic expression of a short segment of ICP22 mimics the effects of P-TEFb inhibition on Pol II transcription (24). Moreover, ICP22 directly interacts with both FACT subunits (25) and ICP22 is required for the redistribution of FACT as well as the DSIF-subunit SPT5 and the elongation factor SPT6 to viral genomes (24). Consistent with ICP22 mimicking the effects of P-TEFb inhibition, Birkenheuer et al. (10) recently found that Pol II pausing was reduced for a subset of host genes in an ICP22-null mutant (ΔICP22) of HSV-1 compared to a repair virus derived from the null mutant with a genetically restored ICP22. For this purpose, they employed precise nuclear run-on followed by deep sequencing (PRO-seq), which sequences RNA that is actively transcribed by Pol II and depicts strand-specific Pol II transcriptional activity. Transcription initiation from most human gene promoters is bidirectional with productive transcription elongation occurring only in the sense direction (26–29). PRO-seq thus provides nucleotide-level resolution of Pol II activity and allows separating sense and antisense Pol II initiation and pausing. Birkenheuer et al. (5) previously also reported that Pol II levels at the promoter-proximal pause site were altered in a gene-specific manner in HSV-1 strain F (WT-F) infection compared to mock. However, they did not explicitly investigate Pol II pausing for host genes in WT-F infection but only for HSV-1 genes in a later study (30). When comparing promoter-proximal Pol II pausing between mock and HSV-1 strain KOS infection using Pol II chromatin immunoprecipitation sequencing (ChIP-seq), Rivas et al. (9) recently found that HSV-1 infection frequently reduced promoter-proximal Pol II pausing, at least for activated genes. This was largely dependent on ICP4. ICP4 is one of five immediate early proteins (including also ICP0, ICP22, ICP27, and ICP47) expressed shortly after infection and is necessary for the transcription of early and late viral genes (31). HSV-1-activated genes exhibited a greater increase in Pol II occupancy on gene bodies than on promoters, consistent with increased transcriptional elongation. For repressed genes, promoter-proximal Pol II pausing was increased independently of ICP4 with Pol II occupancy decreasing more strongly on gene bodies than in the promoter region.

Here, we report on a genome-wide investigation of the impact of HSV-1 infection on promoter-proximal pausing of all expressed host genes. This is based on a reanalysis of PRO-seq data for mock and 3-h p.i. WT-F infection from the study by Birkenheuer et al. (5). Our reanalysis revealed that HSV-1 infection only seemingly leads to a reduction of Pol II pausing for the majority of genes when using standard Pol II pausing index analyses. More detailed analyses, however, demonstrated that Pol II pausing is retained in HSV-1 infection for most host genes but shifted to sites further downstream of the promoter. In contrast to well-defined Pol II pausing peaks at the TSS observed in mock infection, HSV-1 infection resulted in more varied and less well-positioned patterns of Pol II pausing. This included both broadening of Pol II pausing peaks into downstream regions for some genes as well as newly originating or increasing Pol II peaks at downstream sites for other genes. In summary, our study demonstrates that HSV-1 impacts promoter-proximal Pol II pausing in a more complex and unexpected manner than previously thought.

## RESULTS

### Widespread changes in promoter-proximal Pol II pausing during HSV-1 infection.

The standard measure for quantifying promoter-proximal pausing is the so-called pausing index (PI) of a gene, which is calculated as the ratio of normalized read counts in a window around the TSS (=promoter window) divided by normalized read counts in a window on the gene body excluding the promoter. We thus started by performing a genome-wide PI analysis using the published PRO-seq data of mock and 3-h p.i. WT-F infection from the study by Birkenheuer et al. (5). Notably, PIs were also used by Rivas et al. (9) to quantify the effects of lytic HSV-1 infection on Pol II pausing from Pol II ChIP-seq and by Birkenheuer et al. (10) to determine differences in Pol II pausing between ΔICP22 and repair virus infection. Since annotated gene 5′ ends do not necessarily reflect the used TSS in a cell type and multiple alternative TSSs are often annotated, we first identified the dominantly used TSS for each gene from published PROcap-seq and PRO-seq data of flavopiridol-treated uninfected human foreskin fibroblasts (HFF) (32) (see Materials and Methods). PROcap-seq is a variation of PRO-seq that specifically maps Pol II initiation sites. Flavopiridol inhibits CDK9 and thus arrests Pol II in a paused state at the TSS (33) and allows also identifying the TSS for genes that are not or weakly paused in untreated cells. Consistent peaks in PROcap-seq and PRO-seq of flavopiridol-treated HFF provided an initial set of 136,090 putative TSS positions, which were further filtered to identify high-confidence sites by requiring a maximum distance of 500 bp to the nearest annotated gene. This identified 42,193 potential TSS positions for 7,650 genes (median number of TSS per gene = 4 with a median distance of 42 bp). For each gene, the TSS with the highest expression was selected for further analysis. Although the PRO-seq data by Birkenheuer et al. (5) was obtained in HEp-2 cells, for most genes the identified TSS in HFF matched very well to PRO-seq peaks in mock-infected HEp-2 cells (Fig. S1a in the supplemental material), better than gene 5′ ends annotated in Ensembl (Fig. S1b).

**FIG 1:**
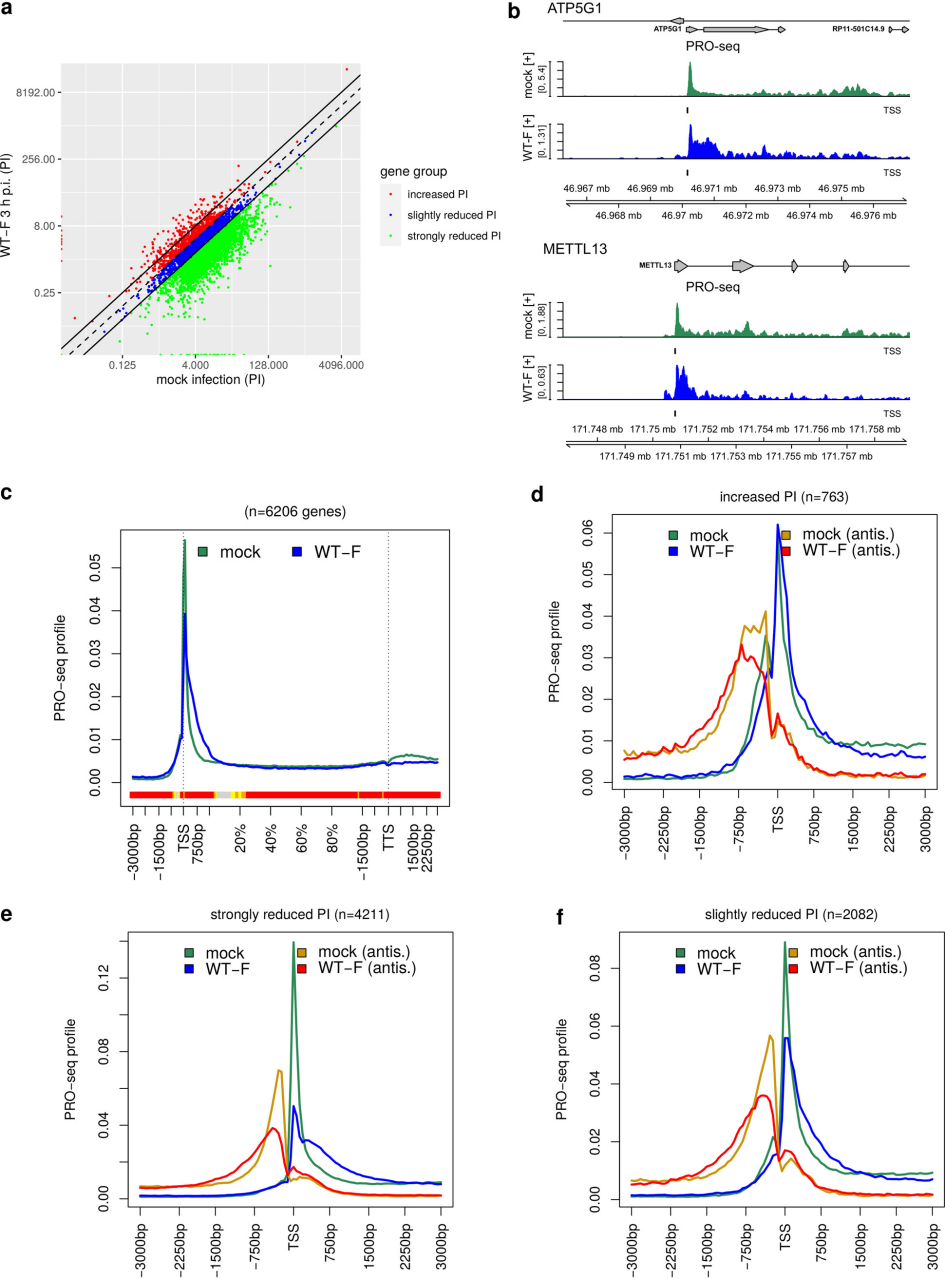
HSV-1 infection impacts promoter-proximal pausing of most host genes. (a) Scatterplots comparing pausing indices (PI) between mock and WT-F infection at 3 h p.i. The dashed line indicates equal PI values and solid lines a 2-fold change in PIs. Genes were divided into three groups according to changes in their PI: (i) increased PI in HSV-1 infection (fold change >1, 763 genes, red); (ii) slightly reduced PI in HSV-1 infection (fold change <1 but ≥0.5, 2,082 genes, blue); and (iii) strongly reduced PI in HSV-1 infection (fold change <0.5, 4,211 genes, green). (b) Read coverage around the TSS in PRO-seq data (sense strand only) for mock (dark green) and WT-F infection at 3 h p.i. (dark blue) for example genes with a reduction in PI upon HSV-1 infection. Read coverage was normalized to total number of mapped reads and averaged between replicates. The identified TSS used in the analysis is indicated by a short vertical line below each read coverage track. Gene annotation is indicated at the top. Boxes represent exons, lines represent introns, and direction is indicated by arrowheads. Genomic coordinates are shown at the bottom. Figures are not centered around the TSS, but a larger region downstream of the TSS was included than upstream of the TSS. (c) Metagene plot showing the distribution of PRO-seq profiles from 3 kb upstream of the TSS to 3 kb downstream of the TTS in sense direction for mock (dark green) and 3-h p.i. WT-F infection (dark blue) for all analyzed genes with a gene length >3 kb. Here, regions from −3 kb to +1.5 kb of the TSS and from −1.5 kb to +3 kb of the TTS were divided into 90-bp bins, respectively, and the remainder of the gene body (+1.5 kb of TSS to −1.5 kb of TTS) into 100 bins of variable length to compare genes with different lengths. Shorter genes were excluded as regions around the TSS and TTS would overlap otherwise, resulting in 6,206 genes. The colored band below the metagene curves indicates the significance of paired Wilcoxon tests comparing the normalized PRO-seq coverages of genes for each bin between mock and 3-h p.i. WT-F infection. *P* values are adjusted for multiple testing with the Bonferroni method within each subfigure; color code: red = adj. *P* value ≤ 10^−15^, orange = adj. *P* value ≤ 10^−10^, yellow = adj. *P* value ≤ 10^−3^. (d to f) Metagene plots showing the distribution of PRO-seq profiles in sense (dark green and blue) and antisense (gold and red) direction from −3 kb to + 3 kb around the TSS for the three gene groups defined in panel a with increased PI (d), strongly reduced PI (e), and slightly reduced PI (f). Mock infection is shown in dark green (sense) and gold (antisense) and WT-F infection at 3 h p.i. in dark blue (sense) and red (antisense). For this purpose, the TSS ± 3 kb promoter window for each gene was divided into 101 bins, and PRO-seq read counts for each sample were determined for each bin, normalized to sequencing depth, and averaged across replicates. Subsequently, bin values for each gene were normalized to sum up to 1 to obtain the Pol II occupancy profile in the promoter window. PRO-seq profiles were determined separately for sense and antisense strands. Results of significance analyses are shown in Fig. S2d to i.

For PI calculation, normalized read counts were determined in a strand-specific manner as reads per kilobase million (RPKM) in the window from the TSS to TSS + 250 bp for the promoter region and from TSS + 250 bp to TSS + 2,250 bp (or the gene 3′ end if closer) for the gene body. It should be noted that there is no consensus on how to best define promoter and gene body windows for PI calculation and a wide range of alternative ranges have previously been used (see, e.g., references 34–36). Genes with zero reads in the promoter or gene body window in mock or WT-F 3 h p.i. were excluded, resulting in PIs for 7,056 genes (Data set S1 in the supplemental material). This analysis showed that for the vast majority of genes PIs were reduced upon 3-h p.i. HSV-1 infection compared to mock (Fig. 1a). Even with very lenient criteria for an increase in PI, i.e., a fold change >1 in HSV-1 infection compared to mock, only 763 genes (10.8%) showed an increase in PI upon HSV-1 infection (red in Fig. 1a). In contrast, 2,082 genes (29.5%) exhibited a slightly reduced PI (fold change <1 but ≥0.5, blue in Fig. 1a) and 4,211 genes (59.7%) showed a strongly reduced PI (fold change <0.5, i.e., more than 2-fold reduced, green in Fig. 1a). Thus, HSV-1 infection induces widespread changes in promoter-proximal Pol II pausing of host genes, resulting in PI reductions for almost all genes and strong PI reductions for the majority of genes. This is consistent with findings by Birkenheuer et al. (5) that Pol II occupancy at promoter-proximal regions was reduced for the majority of genes in WT-F infection compared to mock. While they also found a reduction of Pol II on gene bodies for most of these genes, they did not investigate the relative change between promoter-proximal regions and gene bodies, i.e., the change in PI.

### HSV-1 infection shifts Pol II pausing to downstream sites for most host genes.

A disadvantage of PI analyses is that PIs are not only impacted by increases or decreases in Pol II pausing with decreased or increased elongation across the gene body but also by any alteration in Pol II occupancy affecting the number of reads in either promoter or gene body windows. We thus next investigated read coverage in a genome viewer for several example genes with reduced PI. This indicated that changes in promoter-proximal Pol II pausing during HSV-1 infection were highly complex as exemplified by the ATP5G1 and METTL13 genes in Fig. 1b. Instead of narrow promoter-proximal PRO-seq peaks observed in mock infection, promoter peaks in HSV-1 infection often extended into the gene body by a few hundred nucleotides (e.g., ATP5G1) and/or additional downstream peaks were observed as, e.g., for METTL13. However, at the end of these extended/additional peaks, read levels dropped again to similarly low levels relative to the promoter peak as in uninfected cells. Read levels were not increased across the whole gene body relative to the promoter peak as would be expected with increased levels of elongation and productive transcription. The extended promoter peaks and additional downstream peaks in HSV-1 infection reduced PI values, as they extended >250 bp from the TSS into the gene body. Consequently, gene body RPKM, i.e., the denominator in PI calculation, was increased relative to the promoter RPKM, i.e., the numerator. It should be noted that overall Pol II occupancy was reduced both on the promoter and gene body for the majority of genes during HSV-1 infection as already reported by Birkenheuer et al. (5). To allow comparing the distribution of Pol II occupancy, not absolute levels, and visualize downstream shifts in pausing sites, different scales are used for mock and HSV-1 infection in read coverage plots like Fig. 1b. These reflect the highest values observed in mock and HSV-1 infection, respectively, for the selected genomic region.

To investigate whether such complex pausing changes were a global trend, we performed metagene analyses in ±3 kb windows around promoters for all 7,650 analyzed genes (Fig. S2a, excluding 1 gene without reads in some samples, significance analysis for differences in antisense and sense transcription between mock and WT-F infection in Fig. S2b and c, respectively) as well as separately for the three gene groups defined above based on PI changes (Fig. 1d to f, significance analyses shown in Fig. S2d to i). For metagene analyses, the 6 kb promoter windows for each gene were divided into 101 bins. PRO-seq read counts were determined for each bin in a strand-specific manner, normalized to sequencing depth, and averaged across replicates. Subsequently, bin values for each gene were normalized to sum up to 1 to obtain the Pol II occupancy profile around the promoter for each gene before averaging across all genes. This normalization allows comparing Pol II occupancy around the promoter between genes with different expression levels and makes the analysis independent of global changes in Pol II occupancy between mock and HSV-1 infection. As a consequence, sharp, singular peaks at promoters are characterized by higher peak maxima (e.g., dark green curves in Fig. 1c to f), while broader peaks or multiple peaks have lower peak maxima (e.g., dark blue curves in Fig. 1c, e, and f). Normalization was performed independently for sense and antisense PRO-seq profiles; thus, the height of peaks does not reflect relative levels of sense versus antisense transcription but the distribution of sense and antisense transcription, respectively, around the TSS. To assess the significance of differences between two conditions, Wilcoxon signed-rank tests were performed for each bin in metagene plots comparing normalized coverage values for each gene between the two conditions across all genes. Multiple testing corrected *P* values are color coded at the bottom of metagene plots (red = adjusted [adj.] *P* value ≤ 10^−15^; orange = adj. *P* value ≤ 10^−10^; yellow = adj. *P* value ≤ 10^−3^). If >2 curves are included in metagene plots, significance results for pairwise comparisons are shown in the supplemental material.

Analysis of all genes already showed that PRO-seq profiles for both sense and antisense direction were significantly altered between mock and HSV-1 infection (Fig. S2a to c). In HSV-1 infection, lower Pol II occupancy was observed directly at the TSS and increased occupancy down- and upstream of the TSS for sense and antisense transcription, respectively. This was limited to within 2,250 bp of the TSS in both cases. Metagene analysis on complete genes from the promoter to downstream of the transcription termination site (TTS) confirmed that this relative increase in occupancy downstream of the TSS did not extend across gene bodies (Fig. 1c). Significance analysis showed highly significant differences in the distribution of Pol II occupancy between mock and WT infection for almost the complete gene body, with the notable exception of the region at the end of the promoter window, where increased relative Pol II occupancy in HSV-1 infection downstream of the TSS changed to decreased relative Pol II occupancy on the gene body. The reduction in Pol II occupancy downstream of the TTS during HSV-1 infection reflects the loss of Pol II pausing at the TTS associated with disruption of transcription termination previously reported in HSV-1 infection (8). Interestingly, genes with increased PI upon HSV-1 infection (red in Fig. 1a) only showed a small change in Pol II occupancy in sense direction at the promoter in HSV-1 infection (Fig. 1d; Fig. S2e). In contrast, genes with strong PI reduction upon HSV-1 infection showed a strong reduction of the major peak height at the TSS, a pronounced broadening of the peak into the gene body, and a second minor peak downstream the TSS (Fig. 1e; Fig. S2g). A similar but less pronounced effect was observed for genes with a weak reduction in PI, with a general broadening of the TSS peak but no minor peak (Fig. 1f; Fig. S2i). These changes in the distribution of Pol II occupancy explain the reduction in PIs as read counts in the gene body window are increased relative to read counts in the promoter window.

**FIG 2:**
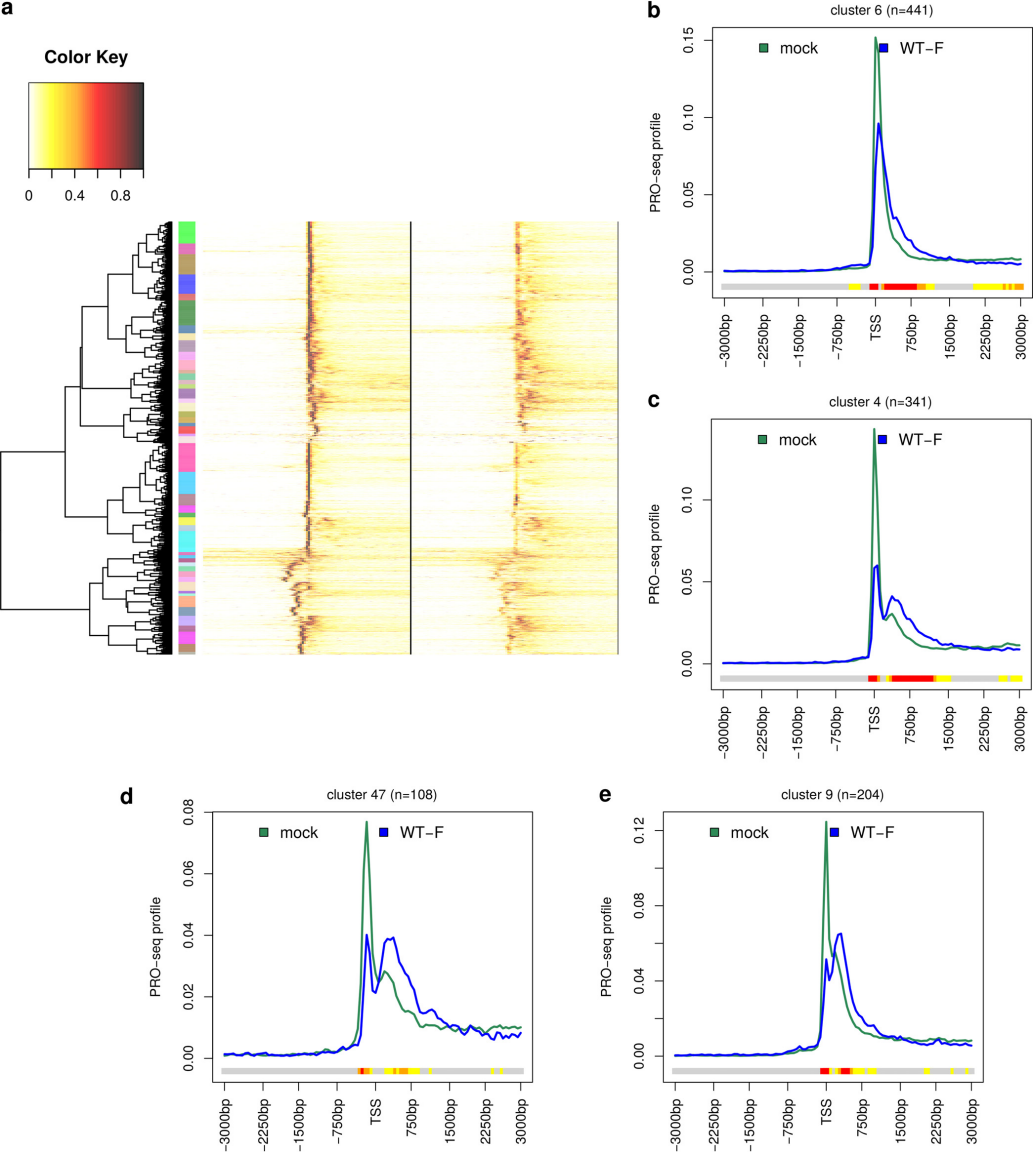
Distinct patterns of changes in promoter-proximal pausing upon HSV-1 infection. (a) Heatmap showing the result of the hierarchical clustering analysis of PRO-seq profiles in mock and WT-F infection. For clustering, PRO-seq profiles in sense direction for mock and WT-F infection were first concatenated and then divided by the maximum value in the concatenated profiles. This resulted in a value of 1 for the position of the highest peak in either mock or HSV-1 infection. Hierarchical clustering was performed according to Euclidean distances and Ward’s clustering criterion, and the cutoff on the hierarchical clustering dendrogram was selected to obtain 50 clusters (marked by colored rectangles between the dendrogram and heatmap). Clusters are numbered from top to bottom. (b to e) Metagene plots of PRO-seq profiles on the sense strand in mock (dark green) and WT-F infection at 3 h p.i. (dark blue) for example clusters 6, 4, 47, and 9 in panel a. See Materials and Methods and Fig. 1 legend for an explanation of metagene plots. The colored bands below the metagene curves in each panel indicate the significance of paired Wilcoxon tests comparing the normalized PRO-seq coverages of genes for each bin between mock and 3-h p.i. WT-F infection. *P* values are adjusted for multiple testing with the Bonferroni method within each subfigure; color code: red = adj. *P* value ≤ 10^−15^, orange = adj. *P* value ≤ 10^−10^, yellow = adj. *P* value ≤ 10^−3^.

To identify groups of genes with distinct patterns of changes of Pol II occupancy around the TSS between HSV-1 and mock infection, we performed hierarchical clustering of genes based on their PRO-seq profiles in sense direction for both mock and HSV-1 infection (Fig. 2a). Since we wanted to ensure that genes with distinct patterns of changes were placed in different clusters, a stringent cutoff was applied on the clustering dendrogram to obtain 50 gene clusters at the cost of obtaining multiple clusters with similar patterns. While most clusters exhibited only a narrow peak at the TSS in mock infection (e.g., Fig. 2b; Fig. S3a, b, f to h), some already exhibited a second minor peak shortly after the TSS already before infection (e.g., Fig. 2c and d; Fig. S3c to e). In addition, a few clusters representing a total of 2,018 genes showed peaks in mock infection that were shifted relative to the TSS we had identified (e.g., Fig. 2d; Fig. S3e). In all cases, the peak was shifted at most 750 bp from the identified TSS and was commonly within 100 to 200 bp of the identified TSS. Since the position of peaks within clusters was highly similar due to the stringent clustering cutoff, analysis of individual clusters thus avoids confounding effects resulting from the misidentification of TSS positions. Typical Pol II occupancy changes upon HSV-1 infection included a reduction of peak height at the TSS with a broadening of the peak into the gene body (e.g., Fig. 2b) and changes in minor and major peak heights in case multiple peaks were already present before infection (e.g., Fig. 2c and d) as well as new peaks originating downstream of the TSS in HSV-1 infection (e.g., Fig. 2e). Figure S4 provides an overview on positions, number, and relative heights of Pol II occupancy peaks in mock and HSV-1 infection for each cluster. In total, 30 clusters shared the same major TSS peak between mock and HSV-1 infection, which included also clusters with only a reduction in peak height but no broadening of the TSS peak (cluster 20; Fig. S3f) and clusters without loss of Pol II pausing (clusters 21 and 27; Fig. S3g and h). Twenty-eight clusters showed a second peak in HSV-1 infection downstream of the TSS peak with a median distance of 480 bp to the major peak (e.g., Fig. 2c and d). For 9 of these 28 clusters, the major TSS peak differed between mock and HSV-1 infection (e.g., Fig. 2e). Almost all clusters exhibited a reduced Pol II peak height at the TSS (except clusters 27 and 21, 262 genes; Fig. S3g and h), and most clusters with a reduced Pol II peak height showed an extension of the peak into the gene body or an increased downstream peak (except clusters 7, 20, 23, 24, and 36, 480 genes). Read coverage plots for example genes from different clusters are shown in Fig. S5, and a UCSC genome browser session showing PRO-seq read coverage for all human genes separately for replicates is available at https://genome.ucsc.edu/cgi-bin/hgTracks?hgS_doOtherUser=submit&hgS_otherUserName=Caroline+Friedel&hgS_otherUserSessionName=PROseq_HSV1.

**FIG 3:**
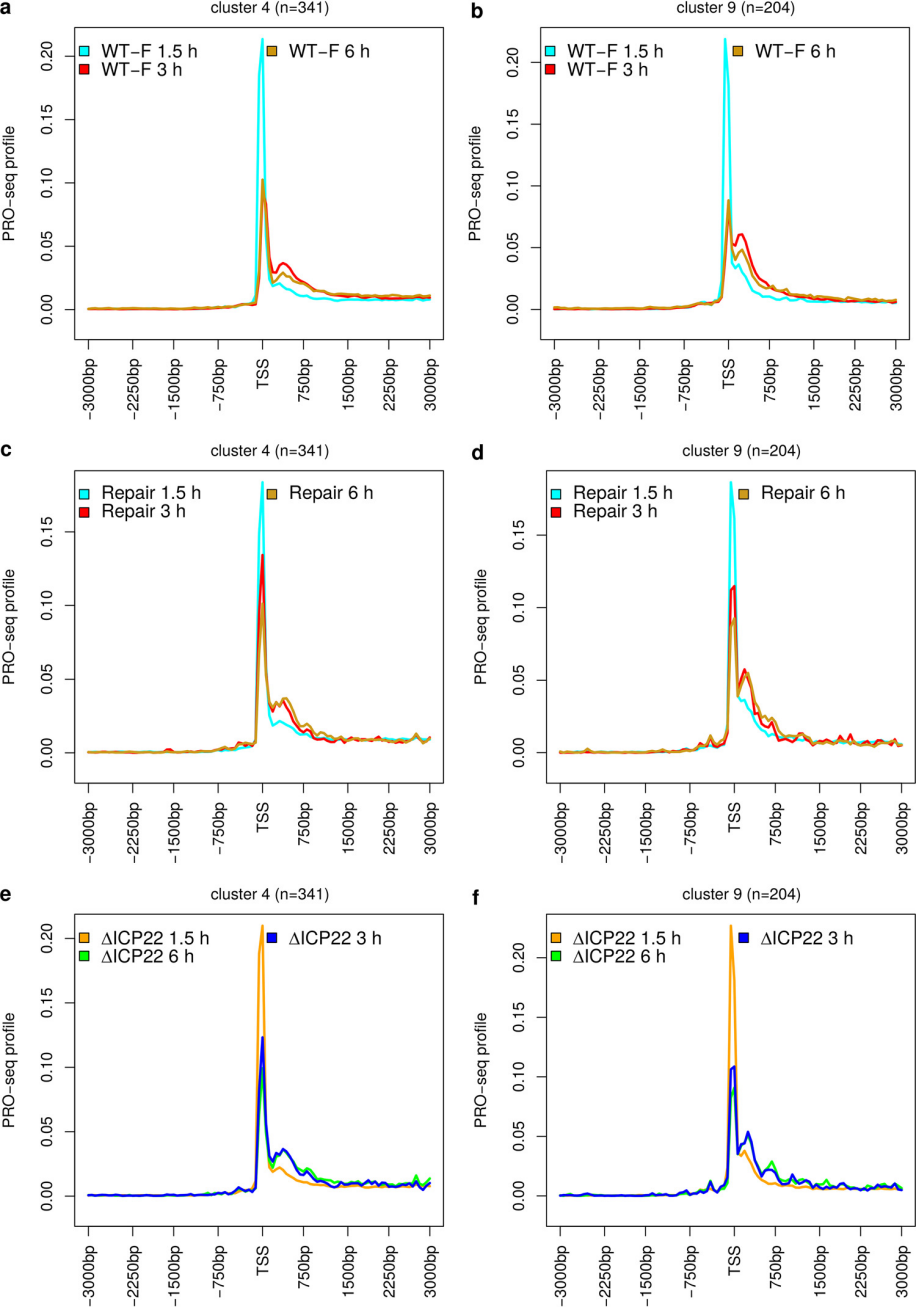
Downstream shift of Pol II pausing throughout the first 6 h of HSV-1 infection and impact of ICP22. Metagene plots of PRO-seq profiles on the sense strand for 1.5-, 3-, and 6-h p.i. WT-F infection (a and b), repair virus infection (c and d), and ΔICP22 infection (e and f) for example clusters in Fig. 2a. Metagene plots with significance analyses of pairwise comparisons between time points for WT-F infection for these and other example clusters can be found in Fig. S6. Metagene plots with significance analyses of pairwise comparisons between repair virus and ΔICP22 infection for the same time points for these and other example clusters can be found in Fig. S7.

Recently, the Baines lab also published PRO-seq data of 1.5-, 3-, and 6-h p.i. WT-F, ΔICP22, and its repair virus infection as well as 3-h p.i. WT-F infection with cycloheximide (CHX) treatment (10, 30, 37). This allowed investigation of both the progression of the changes in Pol II pausing during infection as well as the impact of ICP22. Metagene analyses confirmed the downstream shift of Pol II pausing at 3 and 6 h p.i. for both WT-F and the repair virus (Fig. 3a to d; Fig. S6 for significance analysis of pairwise comparisons for WT-F), with a reduction of the major peak height compared to 1.5 h p.i. and a broadening of the TSS peak or increasing or newly originating downstream peaks. Both 3 and 6 h p.i. differed significantly from 1.5 h p.i., but only a few differences were observed between 3 and 6 h p.i. (Fig. S6). Alterations in pausing were slightly less pronounced at 6 h p.i. than at 3 h p.i. for WT-F infection (Fig. 3a and b; Fig. S6), whereas they were slightly more pronounced at 6 h p.i than at 3 h p.i. for the repair virus infection (Fig. 3c and d). Taken together, these results indicate that HSV-1 infection impacts Pol II pausing already very early in infection (after 1.5 h p.i. but before 3 h p.i.) and that the downstream shift in Pol II pausing remains stable until at least 6 h p.i. ΔICP22 infection generally showed the same trend as the repair virus (Fig. 3e and f) with no significant differences downstream of the TSS between ΔICP22 and repair virus infection at 3 and 6 h p.i. (Fig. S7). For several clusters, a small reduction of the TSS peak was observed in ΔICP22 compared to repair virus infection, which was statistically significant for some of these clusters at either 3 h p.i. (clusters 1, 6, and 13) or 6 h p.i. (clusters 6 and 25). This is consistent with the observation by Birkenheuer et al. (10) that pausing indices were increased in the repair virus compared to ΔICP22 infection for 472 and 721 genes at 3 and 6 h p.i., respectively. In summary, these results show that ICP22 is not required for the downstream shift in Pol II pausing but leads to more retention of Pol II directly at the TSS for some genes. Unexpectedly, at 1.5 h p.i. the opposite effect was observed with a significantly increased Pol II pausing peak at the TSS in ΔICP22 infection and reduced Pol II levels downstream of the TSS. A possible explanation for this observation is that ΔICP22 infection progresses more slowly than repair virus infection such that small effects are already detectable in repair virus infection by 1.5 h but not in ΔICP22 infection. By 3 h p.i., the downstream shift in Pol II pausing is then well established in both viruses. Interestingly, while inhibition of protein translation by CHX during the first 3 h of WT-F infection significantly attenuated changes in pausing both at the TSS and downstream of the TSS, some Pol II pausing changes were still observed in the absence of viral protein translation compared to mock infection (Fig. S8). While most clusters showed increased Pol II levels shortly downstream of the TSS in WT-F infection with CHX treatment compared to mock, indicative of a small downstream shift in Pol II pausing, these differences were not statistically significant. However, for some clusters, Pol II occupancy was significantly reduced at or shortly upstream of the TSS and, for many clusters, it was significantly reduced further downstream of the TSS (>1.5 kb). Although the interpretation of these changes is not straightforward, they indicate a role of viral tegument proteins, e.g., VP16, which also interacts with P-TEFb (24), or a virus entry-induced stress or immune response in manipulating Pol II pausing.

To investigate whether the different patterns in Pol II pausing changes were correlated with gene function or transcription factor binding, we performed over- and underrepresentation analysis for Gene Ontology (GO) terms and transcription factor binding motifs from TRANSFAC for each cluster (Data set S2; adj. *P* value cutoff < 0.001, a stringent *P* value cutoff was chosen to adjust for performing this analysis separately for 50 clusters). This revealed an enrichment of subunits of the spliceosomal snRNP complex, specifically U5 and U6 snRNAs, in cluster 27 (adj. *P* value < 1.31 × 10^−7^), one of the clusters without change in pausing. Since U6 snRNAs are transcribed by RNA polymerase III (38), not Pol II, we investigated the PRO-seq signal of the U6 snRNAs in a genome browser. While some signal was found, it commonly either started already upstream of the U6 snRNA locus or resulted from many reads mapping at the same position. Since U6 snRNA loci are repeated several times in the human genome, we performed a BLAT search for the 70-bp sequence covered by these reads. We found many occurrences of this sequence with few mismatches within Pol II transcribed regions, either in introns of protein-coding genes or in regions downstream of their 3’ end that are still reached by Pol II before RNA cleavage at the upstream poly(A) site. This suggests that these reads were mismapped to other U6 snRNA loci due to sequencing errors making them more similar to these other loci than their actual genomic origins. It is thus not surprising that these loci are enriched in cluster 27, which exhibits no changes between mock and WT-F infection. Nevertheless, they still represent only a very small fraction of this cluster (~7.5%). Cluster 16 was enriched for genes encoding proteins of the large ribosomal subunit (adj. *P* value < 0.00057), but again these represented only a very small fraction (5.9%) of this cluster. No other over- or underrepresentation was observed. Thus, clusters identified based on changes in pausing did not represent functionally related gene groups. Interestingly, however, cluster 32 was strongly enriched for a number of G- or C-rich transcription factor binding motifs, with 90% of genes having a match for a long G-rich motif (GGGMGGGGSSGGGGGGGGGGGG, adj. *P* value < 0.00025). In contrast, several A- and T-rich motifs (e.g., NNNNRNTAATTARY, adj. *P* value < 6.94 × 10^−9^) were underrepresented. The opposite effect was observed for cluster 6, with G-/C-rich motifs being under- and A-/T-rich motifs being overrepresented. A few G-/C-rich motifs were also underrepresented in cluster 10. Recently, Watts et al. showed that GC content is high around pause sites and that GC skew [= (G − C)/(G + C)] peaks in the 100 nt upstream of the pause site (39). Analysis of GC content and GC skew around the TSS for individual clusters indeed showed a high GC content for cluster 32 at and downstream of the TSS, although no peak in GC skew (Fig. S9a). In contrast, GC content was less increased around the TSS for cluster 6, while GC skew peaked at the TSS and was increased downstream of the TSS (Fig. S9b). As clusters 32 and 6 differed considerably regarding the change in Pol II pausing upon HSV-1 infection, this raises the possibility that sequence composition around the TSS could play a role in determining the changes in Pol II pausing upon HSV-1 infection. However, analysis of the other clusters did not reveal any consistent trend in GC content or GC skew that explained differences in Pol II pausing between clusters. The one consistent trend we observed was that clusters for which the major PRO-seq peak was significantly upstream of our identified TSS commonly exhibited a plateau of high GC content starting at the PRO-seq peak and extending to the TSS (e.g., Fig. S9c and d). It should be noted that the HSV-1 genome is highly GC rich (~68%, Fig. S9e) and almost 50% of viral genome positions have a GC content at least as high as observed at host pause sites (70%; reference 39). Consistently, many occurrences of the G/C-rich over- or underrepresented transcription factor binding motifs for clusters 6 and 32 can be found in the HSV-1 genome (Fig. S9e), with these occurrences being even more G/C-rich than the viral genome overall.

**FIG 4:**
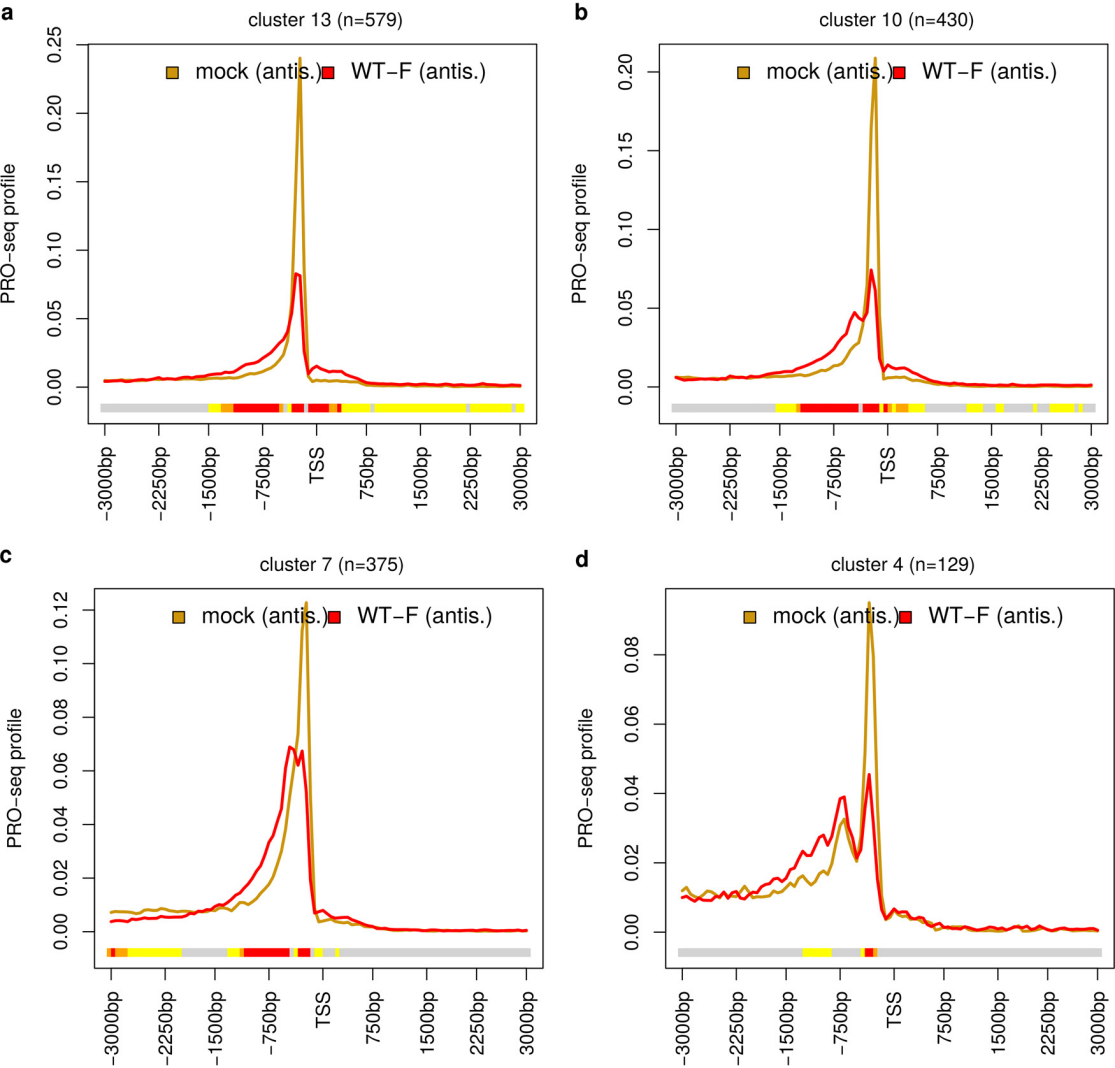
Changes in antisense promoter-proximal pausing in HSV-1 infection. Metagene plots of PRO-seq profiles on antisense direction in mock (gold) and WT-F infection at 3 h p.i. (red) for example clusters resulting from the hierarchical clustering of genes according to antisense PRO-seq profiles in mock and WT-F infection. Here, clustering was performed as described in Fig. 2a legend but applied to concatenated antisense PRO-seq profiles in mock and WT-F infection. Thus, clusters shown here differ from the clusters shown in all other figures. (a) The most common pattern observed for almost all clusters with a broadening of the antisense PRO-seq peak at the TSS. (b to d) The only three clusters that exhibit different patterns with additional peaks originating or increasing in antisense direction during infection. See Materials and Methods and Fig. 1 and 2 legends for the explanation of metagene plots.

Considering the bidirectionality of transcription initiation at human promoters, we also performed metagene analyses of PRO-seq profiles in antisense direction. Antisense transcription initiation at bidirectional promoters commonly only results in short, unspliced, nonpolyadenylated, and unstable upstream antisense RNAs (uaRNAs) (40) that have highly heterogeneous 3′ ends (41). The metagene analyses of antisense PRO-seq profiles for all genes as well as genes grouped by PI changes also showed a significant reduction in the antisense TSS peak height and a broadening of the peak in the antisense direction (Fig. 1d to f; Fig. S2). However, clustering of antisense PRO-seq profiles in mock and HSV-1 infection with the same approach as for sense profiles to obtain 50 clusters did not identify different patterns between clusters. Most of the 50 antisense clusters exhibited only the same pattern as the metagene analysis of all genes (e.g., Fig. 4a). Only two clusters (430 and 375 genes) showed a small secondary antisense peak originating in HSV-1 infection in addition to the broadening of the antisense signal upstream of the TSS (Fig. 4b and c). One other cluster (129 genes) showed a secondary peak that was already present in mock infection but increased relative to the TSS peak in WT infection (Fig. 4d); however, the increase at this secondary peak was not statistically significant.

### Delayed pausing is not an artifact of *de novo* transcription initiation or read-through transcription.

**FIG 5:**
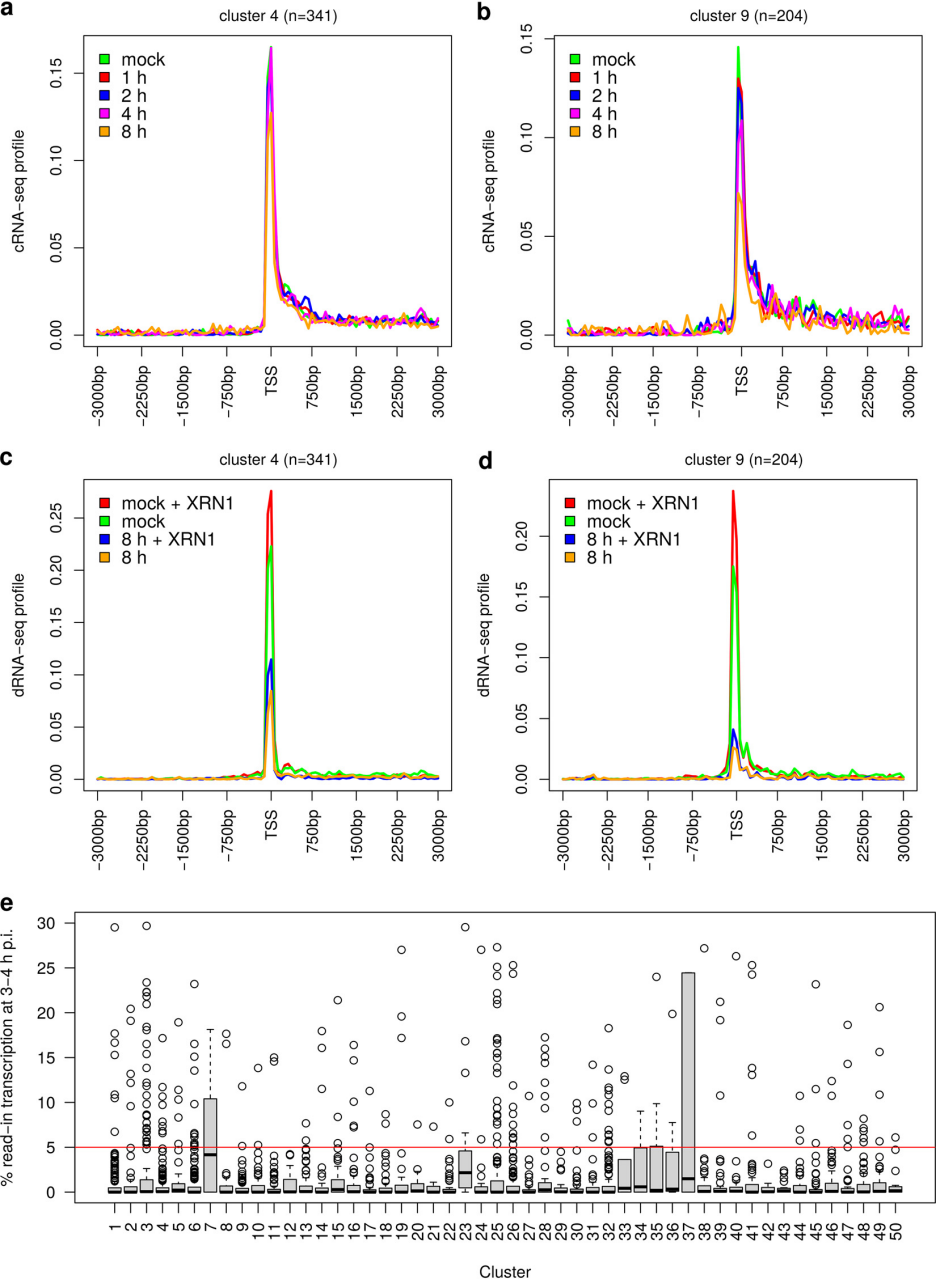
Delayed pausing is not an artifact of alternative *de novo* initiation or read-in transcription. (a to d) Metagene plots of cRNA-seq profiles on the sense strand in mock and WT-17 infection at 1, 2, 4, and 8 h p.i. (a and b) and dRNA-seq profiles on the sense strand in mock and 8-h p.i. WT-17 infection (c and d) with and without XRN1 treatment for example clusters 4 and 9, which show broadening of peaks or additional peaks originating or increasing in height in PRO-seq data during WT-F infection. For metagene plots of PRO-seq profiles for these clusters, see Fig. 2c and e. (e) Boxplots showing the distribution of read-in transcription at 3 to 4 h p.i. for genes in the 50 clusters identified from sense PRO-seq profiles. Boxes represent the range between the first and third quartiles for each cluster. Black horizontal lines in boxes show the median. The ends of the whiskers (vertical lines) extend the box by 1.5 times the interquartile range. Data points outside this range (outliers) are shown as small circles. The red horizontal line indicates the cutoff we previously used to determine that no read-in transcription is observed (≤5% read-in transcription). Metagene plots of PRO-seq profiles for clusters 7, 23, and 33 to 37 with some read-in transcription observed at 3 to 4 h p.i. are shown in Fig. S14.

Since increasing or newly originating secondary PRO-seq peaks could also represent alternative transcription initiation, we next investigated the presence of alternative TSSs for all clusters in either the PROcap-seq and PRO-seq data of flavopiridol-treated HFF or the human genome annotation. For most clusters, <15% of genes showed evidence for an alternative TSS at the additional peak positions in either flavopiridol-treated cells (e.g., Fig. S10a and c) or the genome annotation (e.g., Fig. S10b and d). This is in clear contrast to clusters in which the TSS identified from flavopiridol-treated HFF did not represent the dominant TSS in HEp-2 cells. Here, almost 50% of genes had an additional peak in flavopiridol-treated cells or an annotated transcript start at the position of the dominant TSS in HEp-2 cells (e.g., Fig. S10e and f). We furthermore investigated induction of alternative *de novo* transcription initiation downstream of the TSS during HSV-1 infection using cRNA-seq and directional RNA-seq (dRNA-seq) data of transcript 5′ ends for mock and HSV-1 strain 17 (WT-17) infection of HFF from our recent reannotation of the HSV-1 genome (*n* = 2 replicates) (42). cRNA-seq is based on circularization of RNA fragments. dRNA-seq is based on selective cloning and sequencing of the 5′ ends of cap-protected RNA molecules resistant to the 5′–3′-exonuclease XRN1. Both methods strongly enrich reads from 5′-RNA ends. cRNA-seq was performed for mock and 1-, 2-, 4-, 6-, and 8-h p.i. HSV-1 infection. dRNA-seq was performed for mock and 8-h p.i. HSV-1 infection with and without XRN1 treatment. Metagene analyses of cRNA- and dRNA-seq data showed clear peaks coinciding with the major PRO-seq peaks in mock infection and smaller peaks at minor PRO-seq peaks already present in mock infection (Fig. 5a to d; Fig. S11 and S12). In contrast, no (increased) peaks were observed at the positions of downstream PRO-seq peaks that increased or newly originated during HSV-1 infection. This was the case both early (2 and 4 h p.i. in cRNA-seq; Fig. 5a and b; Fig. S11) and later in infection (6 and 8 h p.i. in cRNA-seq, Fig. 5a and b and Fig. S11; 8 h p.i. in dRNA-seq, Fig. 5c and d; Fig. S12) around the 3 and 6 p.i. time points when the downstream shift of Pol II pausing was observed in the PRO-seq data.

Since cRNA- and dRNA-seq were obtained for WT-17 infection, while PRO-seq was performed for WT-F infection, we compared expression changes for host genes between WT-17 and WT-F infection to assess the similarity of virus-induced expression changes. For this purpose, we analyzed total RNA-seq data for WT-17 infection at 8 and 12 h p.i. and WT-F infection at 8 h and 12 h p.i. (Fig. S13). Total RNA-seq data for (i) mock and 8-h p.i. WT-17 infection were taken from our previous study (8), for (ii) mock and 12-h p.i. WT-17 infection from the study by Pheasant et al. (43), and for (iii) mock and 8- and 12-h p.i. WT-F infection from our recent study (44). Since RNA-seq data for WT-F at 8 and 12 h p.i. were obtained in the same experiment and thus expected to be more similar due to less technical noise, we included RNA-seq for WT-17 infection from two different sources to assess the extent of differences that can be ascribed to experimental noise rather than differences between virus strains. This analysis showed that gene expression fold changes compared to mock were highly correlated between WT-17 and WT-F infection both for 8 and 12 h p.i. (Fig. S13a and b), with most genes showing less than a 2-fold difference (indicated by gray lines). Comparison of fold changes for 8 and 12 h p.i. for WT-17 infection between separate experiments (Fig. S13c) showed that observed differences between WT-17 and WT-F were within the range of differences observed in separate experiments. In contrast, fold changes were highly similar between 8 and 12 h p.i. from the same experiment (Fig. S13d). Moreover, when comparing differentially expressed genes (*P* < 0.01) across time points and strains (Fig. S13e), we observed a high consistency across all four conditions, with differences reflecting more the source of the data than the HSV-1 strain. For instance, a group of genes (marked by a red rectangle in Fig. S13e) was partly downregulated in the 12-h p.i. WT-17 data from Pheasant et al. (43) but generally upregulated in the WT-17 8-h p.i and WT-F data from our lab. Since total RNA-seq reflects the cumulative effect of viral infection on host expression up to this time point, the similarity observed between WT-17 and WT-F infection late in infection confirms a strong concordance in virus-induced host expression changes up to this time point between the two strains. We conclude that changes in Pol II occupancy during HSV-1 infection are not due to alternative initiation at novel TSSs leading to capped transcripts. However, we cannot fully exclude that some may reflect abortive *de novo* initiation at novel initiation sites downstream of the TSS. This analysis also excludes Pol II creeping, which is observed upon H_2_O_2_ treatment (45), as the latter would lead to signals from capped transcripts increasing downstream of the TSS in the pausing region.

We previously showed that late in infection “read-in” transcription originating from disrupted transcription termination for an upstream gene commonly extends into downstream genes, which can be mistaken for induction of downstream genes (8). Although read-in transcription only affected very few genes within the first 4 h p.i., increased Pol II occupancy downstream of the TSS could potentially originate from read-in transcription. To quantify read-in transcription, we used our previously published 4-thiouridine sequencing (4sU-seq) time course for every hour of the first 8 h of lytic HSV-1 strain 17 (WT-17) infection of HFF (8). 4sU-seq sequences newly transcribed RNA obtained by labeling with 4sU in specific time intervals of infection (here: 1-h intervals for the first 8 h of lytic expression). Read-in transcription was quantified as previously described (see references 7 and 46 and Materials and Methods for details). In brief, we first calculated the percentage of upstream transcription (=transcription in a 5-kb window upstream of the gene 5′ end/gene expression) for mock infection and each 1-h window of HSV-1 infection. Subsequently, the percentage of read-in transcription was calculated by subtracting values in mock infection from values in each 1-h window of HSV-1 infection (multiplied by 100, negative values set to zero). This analysis included only genes with ≥5 kb to the next up- or downstream gene. By 3 to 4 h p.i., read-in transcription was essentially absent (i.e., much less than 5%) for almost all genes in nearly all clusters (Fig. 5e). Only a few clusters (clusters 7, 23, 33, to 37) exhibited a small extent of read-in transcription already this early in infection; however, these clusters did not exhibit substantial downstream shifts in Pol II occupancy or additional secondary peaks (Fig. S14). The largest of these clusters, cluster 7, indeed showed significantly increased Pol II occupancy in the sense direction upstream of the TSS in HSV-1 infection (Fig. S14a), consistent with read-in transcription. We conclude that read-in transcription extending (partially) into downstream genes does not explain extended TSS peaks or novel or increasing downstream peaks in Pol II occupancy observed in HSV-1 infection.

### Delayed pausing in HSV-1 infection occurs downstream of secondary pause sites used upon NELF depletion.

**FIG 6:**
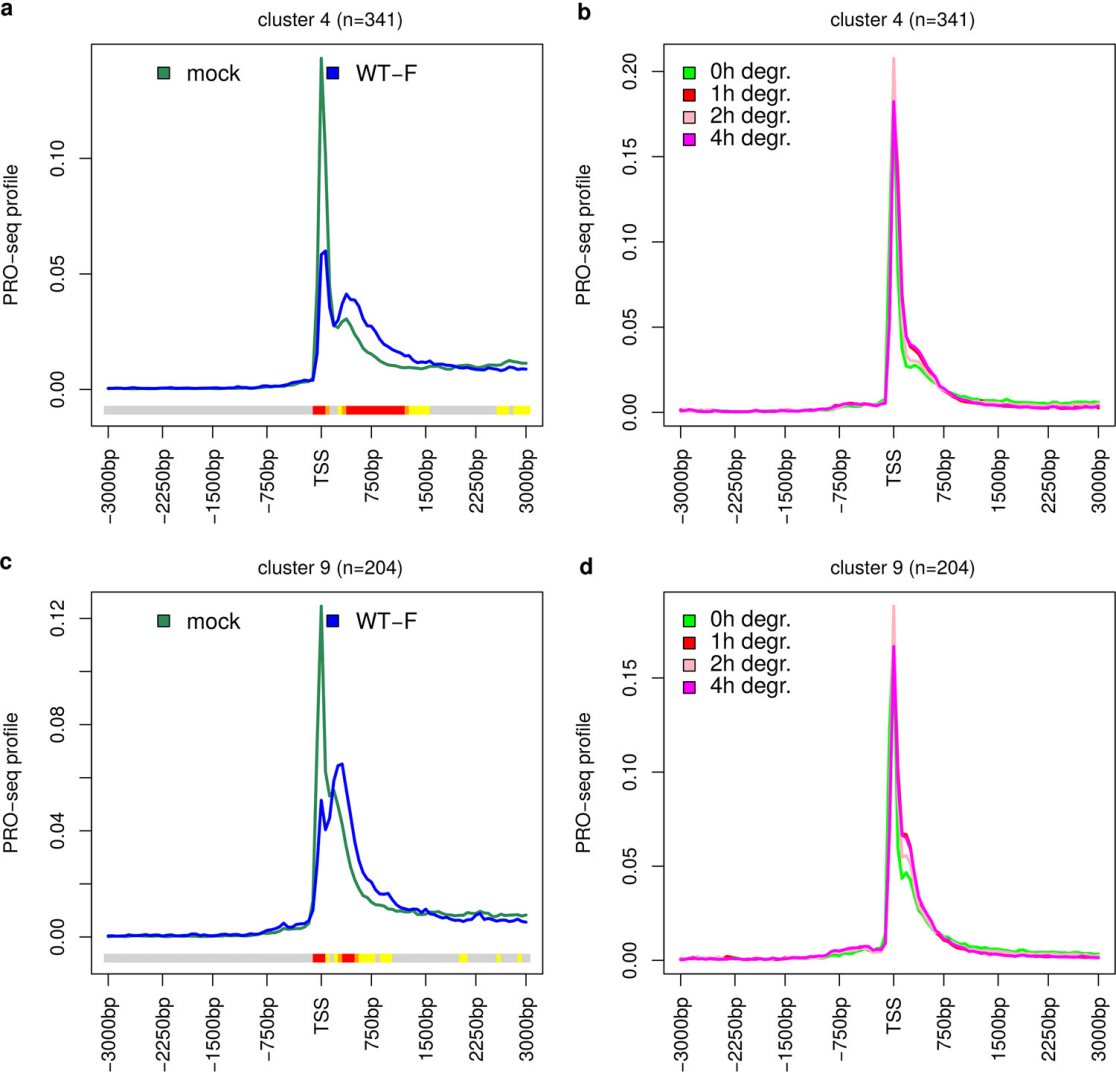
HSV-1 infection leads to stronger downstream shifts in pause sites than NELF depletion. Metagene plots around the TSS of PRO-seq profiles for mock and 3-h p.i. WT-F infection from the study of Birkenheuer et al. (5) (a and c) and 0-, 1-, 2-, and 4-h auxin-inducible degradation of NELF from the study by Aoi et al. (47) (b and d) for example clusters showing a broadening of the TSS peak (a and b) or a small downstream peak (c and d) upon NELF degradation. See Materials and Methods and Fig. 1 and 2 legends for the explanation of metagene plots.

Recently, Aoi et al. (47) showed that rapid depletion of NELF, the key mediator of Pol II pausing, does not completely abolish pausing. Instead, Pol II is paused at a secondary more downstream pause site around the +1 nucleosome. Since Rivas et al. (9) showed an ICP4-dependent decrease of NELF in the promoter-proximal region of some HSV-1-activated genes, we reanalyzed PRO-seq data from the study of Aoi et al. (47) for 0-, 1-, 2-, and 4-h auxin-induced degradation of NELF for our 50 clusters to investigate whether changes in pausing upon NELF depletion showed similarities to changes of HSV-1 infection. For this purpose, we also used the TSS positions identified from the PROcap-seq and PRO-seq data of flavopiridol-treated HFF for the NELF degradation data. Although Aoi et al. performed PRO-seq in DLD-1 (colorectal adenocarcinoma) cells, our identified TSS matched well to PRO-seq peak positions for 0 h NELF degradation in these cells (Fig. S15a). This was also confirmed in metagene analyses for our 50 clusters (Fig. 6b and d; Fig. S15c to k).

In the metagene analyses, we indeed observed an increased second PRO-seq peak upon NELF degradation or a broadening of the first PRO-seq peak downstream of the TSS for a few of our clusters (e.g., Fig. 6; Fig. S15b to e). For most clusters, however, we only observed a reduction in the major peak height and a minor broadening of the peak into downstream regions (e.g., Fig. S15f to k). In either case, the changes in the distribution of Pol II occupancy in HSV-1 infection were much more pronounced than after NELF depletion, with more extensive broadening of peaks and new secondary peaks arising further downstream of the major peak. In summary, delayed pause sites in HSV-1 infection are further downstream than “normal” secondary pause sites at +1 nucleosome positions used upon NELF depletion.

## DISCUSSION

Promoter-proximal Pol II pausing is a key regulatory step between transcription initiation and productive elongation. HSV-1 infection has previously been reported to dramatically impact Pol II positioning on host genes, including promoter-proximal regions (5). Promoter-proximal Pol II pausing on HSV-1 genes also plays a key role in HSV-1 transcription (30). Rivas et al. (9) recently reported that HSV-1 infection leads to a reduction in pausing indices for activated host genes and an increase in pausing indices at repressed host genes. While our reanalysis of PRO-seq data of mock and 3-h p.i. WT-F infection also showed a reduction of pausing indices for most expressed host genes during HSV-1 infection, it also illustrated that pausing indices are an inadequate measure of promoter-proximal Pol II pausing. Pausing indices are altered by any change in the distribution of Pol II between the promoter and gene body. Thus, more in-depth analyses are necessary to characterize changes in promoter-proximal Pol II pausing, not only during HSV-1 infection. Our metagene analyses revealed that HSV-1 infection does not lead to a simple reduction of promoter-proximal Pol II pausing with a relative increase of elongating Pol II on the whole gene body. Instead, we observed that Pol II pausing is retained for the vast majority of genes but is shifted to downstream pause sites. This is reflected in broadened Pol II promoter-proximal peaks that extend further into the gene body and newly originating or increasing downstream peaks. A fine-grained clustering analysis identified a wide range of different patterns for different genes in HSV-1 infection, which contrasts with the sharp promoter-proximal peaks commonly observed in uninfected cells. This indicates that the positioning of shifted pause sites in HSV-1 infection is less well defined than that of “normal” pause sites in uninfected cells. Pronounced downstream shifts of Pol II pausing were only observed after 1.5 h p.i. but remained stable until at least 6 h p.i. Analysis of transcript start site profiling for early (2 and 4 h p.i.) and later (6 and 8 h p.i.) infection time points and of newly transcribed RNA in HSV-1 infection excluded that this was due to *de novo* initiation at downstream sites or read-in transcription originating from disrupted transcription termination for upstream genes.

Interestingly, analysis of promoter-proximal pausing in the antisense direction also showed a broadening of antisense TSS peaks upon HSV-1 infection, with antisense transcription extending further upstream of the TSS than in uninfected cells. However, secondary antisense peaks were only observed for a small fraction of genes (12.2%). It has been proposed that Pol II is particularly prone to pausing and termination during early elongation, specifically on AT-rich sequences often found upstream of promoters (48). Previously, we reported both widespread disruption of transcription termination in HSV-1 infection (8) and activation of antisense transcription at promoters and within gene bodies (49). It is thus tempting to speculate that activation of antisense transcription in HSV-1 infection could be linked to alterations in antisense Pol II pausing, potentially in combination with disruption of transcription termination in the antisense direction.

Nucleosomes represent a natural barrier to transcription and are disassembled before and reassembled after transcribing Pol II (50). Nucleosomes directly downstream of the TSS are generally well positioned at specific locations, in particular the +1 nucleosome, but less so further up- or downstream (51–53). In the presence of NELF, Pol II pausing occurs between the promoter and the +1 nucleosome, and strong positioning of the +1 nucleosome increases pausing (54). While NELF has previously been considered to be required for establishing Pol II pausing, rapid depletion of NELF using auxin-inducible degron does not abolish pausing (47). Instead, pausing appears to be a two-step process with Pol II transitioning from the first to a secondary pause site associated with +1 nucleosomes upon NELF depletion. As Rivas et al. (9) reported decreased levels of NELF at promoter regions of four activated genes tested by ChIP, depletion of NELF from host promoters may play a role in the downstream shift of promoter-proximal Pol II pausing in HSV-1 infection. Notably, a few clusters with additional downstream peaks observed in HSV-1 infection already showed small and much less pronounced peaks at these positions in mock infection. This supports the hypothesis that loss of pausing at major pause sites upon HSV-1 infection leads to pausing of Pol II at secondary downstream pause sites. However, a comparison of the effects of NELF degradation and HSV-1 infection for the 50 identified clusters showed that HSV-1 infection led to much more pronounced alterations in Pol II pausing and more extensive downstream shifts of pause sites than degradation of NELF. We conclude that NELF depletion at promoters alone is unlikely to explain the delay in Pol II pausing observed in HSV-1 infection.

The role of ICP22 in shaping Pol II pausing during WT HSV-1 infection remains an intriguing open question given its previously reported inhibition of the CDK9 subunit of P-TEFb (23). Moreover, lack of ICP22 during HSV-1 infection reduces promoter-proximal Pol II pausing of immediate early genes ICP4, ICP0, and ICP27 and some host genes compared to infection with a repair virus carrying ICP22 (10). We therefore investigated whether infection with an ICP22-null mutant (10) exhibited differences in Pol II pausing compared to the repair virus. While we observed small differences directly at the TSS for some genes, no significant differences between ΔICP22 and the repair virus were detected downstream of the TSS at 3 and 6 h p.i. These results confirm an effect of ICP22 on pausing directly at the TSS for some host genes but also show that ICP22 is not required for the widespread downstream shift of Pol II pausing. Interestingly, global inhibition of protein translation and thus *de novo* lytic viral gene expression by CHX during the first 3 h of HSV-1 infection increased pausing peaks directly at the TSS and largely abolished the downstream shift in Pol II pausing but still resulted in differences in Pol II occupancy in the promoter region compared to mock infection. The HSV-1 tegument protein VP16 delivered with incoming virions contains a region with structural similarity to ICP22 that interacts with P-TEFb (24). Furthermore, the coexpression of VP16 and ICP22 restores phosphorylation of CDK9 pThr186, a mark of CDK9 activity, which is reduced when expressing ICP22 on its own (24). Based on these and previous findings, Isa et al. (24) proposed a model in which ICP22 activity leads to promoter-proximal stalling and premature termination of elongating Pol II on cellular genes, which is attenuated by VP16 to redirect cellular resources for transcription of viral immediate early genes. This raises the possibility that VP16 plays a role in HSV-1-mediated changes in Pol II pausing. Considering the many ways HSV-1 manipulates the host transcription factory, including other factors involved in Pol II pausing like FACT, SPT5, and SPT6 (required for RNA Pol II progression through +1 and subsequent nucleosomes; reference 55), it is likely that no single viral protein is solely responsible. As such, the downstream shift in Pol II pausing may simply be a by-product of other processes ongoing in HSV-1 infection, e.g., the general loss of Pol II and elongation factors from the host genome and their recruitment to viral genomes. Moreover, considering the high GC content of the HSV-1 genome (68%) similar to the GC content at host pause sites (70%; reference 39) and evidence that high GC content stabilizes DNA-RNA hybrids downstream of the pause site and in this way contributes to pausing (56), HSV-1 may need to manipulate host pausing factors to alleviate Pol II pausing at viral promoters and allow active elongation for viral genes. In this case, the downstream shift of Pol II pausing on host genes may simply be a bystander effect.

The functional impact of the observed changes in promoter-proximal Pol II pausing during HSV-1 infection also remains unclear. Rivas et al. (9) concluded that ICP4 activates host genes by promoting the release of paused Pol II into elongation. However, our analysis showed that Pol II is not fully released from pausing but pausing is shifted to downstream sites for most genes. Nevertheless, the global changes in pausing might still serve to promote increased elongation for a few genes as some genes strongly upregulated in total RNA indeed exhibited increased elongation rather than delayed pausing. This is exemplified by the JUNB gene in Fig. S16a. JUNB encodes the JunB subunit of the heterodimeric AP-1 transcription complex, which is composed of members of the JUN, FOS, ATF, and MAF protein families (57). JUNB is an immediate early gene induced rapidly and transiently by various stimuli and depletion of a NELF subunit increased JUNB expression both before and after induction by interleukin-6 stimulation (58), indicating that NELF-mediated pausing is involved in attenuating JUNB expression. HSV-1 infection has been shown to activate AP-1 binding activity via JNK/SAPK and p38 MAPK pathways, with JunB and JunD being the major AP-1 components by 11 h p.i. (59). While the role of AP-1 in HSV-1 infection has not been completely resolved, AP-1 has recently been reported to induce a gene encoding miR-24, a microRNA that dampens the host antiviral response to HSV-1 (60). On the other hand, the downstream shift in Pol II pausing might also have a negative effect on transcription for affected genes. Premature termination at promoter-proximal pausing sites is both an essential aspect of gene regulation and a response to the accumulation of Pol II stalling and arrest (61). It would thus be tempting to speculate that alterations of Pol II pausing lead to increased premature termination and in this way contribute to the loss of host transcriptional activity. Since transcripts terminated prematurely close to the TSS are generally unprocessed and nonpolyadenylated and thus rapidly degraded (62), they are unlikely to have any functional impact themselves. What lends some weight to this hypothesis is that a number of genes with shifted Pol II pausing play a role in antiviral responses. For instance, METTL3 (Fig. S16b) stabilizes IRF3 mRNA via *N*^6^-methyladenosine modification, and type I interferon (IFN) induction, e.g., in response to HSV-1 infection, is impaired in METTL3 knockout cells (63). In contrast, overexpression of METTL3 enhanced type I IFN induction by HSV-1 (63). Similarly, the DExD-box RNA helicase DDX50 (Fig. S16c) activates the IRF3 signaling pathway following infection with RNA and DNA viruses, including an ICP0-null mutant of HSV-1 (64). Several other DExD/H-box helicases, many of which have been identified as regulators of antiviral innate immunity (65), show shifts in Pol II pausing, e.g., DHX36 (Fig. S16d, involved in DNA virus sensing; reference 66) or DDX3X (Fig. S5a, contributes to IRF3 activation; reference 67). Notably, however, HSV-1 depends on optimal DDX3X protein levels for viral gene expression, replication, propagation, and infectivity and incorporates DDX3X proteins into mature particles (68–70). This highlights that it is difficult to fully appreciate the functional impact of downstream shifts in Pol II pausing on virus infection.

Finally, it should be noted that our study has also important implications for the analysis of functional genomics studies on HSV-1 and potentially other viral infections. As already observed in previous studies reporting on disruption of transcription termination or activation of antisense transcription observed upon HSV-1 infection (8, 49), standard sequencing data analysis methods are not designed and are thus insufficient to uncover previously unsuspected alterations in transcription. Thus, more in-depth analyses and customized methods are required. In summary, our study highlights a novel aspect in which HSV-1 infection fundamentally alters the host transcriptional cycle, which has implications for our understanding not only of HSV-1 infection but also of the maintenance of Pol II pausing in eukaryotic cells.

## MATERIALS AND METHODS

### Previously published sequencing data analyzed in this study.

PROcap-seq and PRO-seq data of flavopiridol-treated uninfected HFF cells were taken from the study by Parida et al. (32) (GEO accession: GSE113394, samples GSM3104917 and GSM3104913). PRO-seq data for mock and WT-F infection at 3 h p.i. of HEp-2 cells were taken from the study by Birkenheuer et al. (5) (*n* = 3 replicates, GEO accession: GSE106126, samples GSM2830123 to GSM2830127). PRO-seq data of HEp-2 cells for 1.5, 3, and 6 h of WT-F, ΔICP22, and ICP22 repair virus infection and 3-h p.i. WT-F infection + CHX treatment were taken from studies by Birkenheuer et al. (30) and Dunn et al. (37) (*n* = 2 to 6, GEO accessions: GSE130342, samples GSM3736426 to GSM3736437; GSE169574, samples GSM5210187 to GSM5210194; GSE202363, samples GSM6112020 to GSM6112028). PRO-seq data for 0, 1, 2, and 4 h of auxin-induced degradation of NELF were taken from the study by Aoi et al. (47) (*n* = 1 apart from 0 h with *n* = 2, GEO accession: GSE144786, samples GSM4296314 to GSM4296316, GSM4296318, and GSM4296319). dRNA-seq data for mock and 8-h p.i. HSV-1 infection with and without XRN1 treatment and cRNA-seq data for mock and 1-, 2-, 4-, 6-, and 8-h p.i. HSV-1 infection of HFFF was taken from our previous study (42) (*n* = 2, GSE128324, samples GSM3671394 to GSM3671411). 4sU-seq data for mock and hourly intervals for the first 8 h of WT-17 infection of HFFF were taken from our previous study (8) (*n* = 2, GEO accession: GSE59717, samples GSM1444171 to GSM1444179, GSM1444185 to GSM1444193). Total RNA-seq for mock and WT-F and WT-17 infection at 8 and 12 h p.i. were taken from our previous studies (8, 44) (*n* = 2, GEO accession: GSE59717, samples GSM1444166, GSM1444170, GSM1444180, GSM1444193; GSE185239, samples GSM5608630 to GSM5608643 without PAA) and the study by Pheasant et al. (43) (*n* = 5, SRA accession: SRP168592, samples SRR8187008 to SRR8187014, SRR8186995 to SRR8186999).

### Read alignment.

The read alignment pipeline was implemented and run in the workflow management system Watchdog (71, 72). Published sequencing data were first downloaded from SRA using the sratoolkit version 2.10.8. Sequencing reads were aligned against the human genome (GRCh37/hg19) and human rRNA sequences using ContextMap2 version 2.7.9 (73) (using BWA as short read aligner (74) and allowing a maximum indel size of 3 and at most 5 mismatches). PRO-seq reads commonly contain parts of sequence adapters that cannot be aligned to the genome. While these can be removed before alignment using, e.g., cutadapt (75) as outlined in the protocol by Mahat et al. (76), we did not include it in our workflow as ContextMap2 automatically trims parts of reads that cannot be aligned to the genome. As a consequence, adapter sequences were automatically removed during alignment. For sequencing data of HSV-1 infection, alignment also included the HSV-1 genome (human herpesvirus 1 strain 17, GenBank accession code: JN555585). For the two repeat regions in the HSV-1 genome, only one copy was retained each, excluding nucleotides 1 to 9,213 and 145,590 to 152,222 from the alignment. SAM output files of ContextMap2 were converted to BAM files using samtools (77). Read coverage in bedGraph format was calculated from BAM files using BEDTools (78).

### Data plotting and statistical analysis.

All figures were created in R, and all statistical analyses were performed in R (79). Read coverage plots were created using the R Bioconductor package Gviz (80).

### Transcription start site identification.

We used the iTiSS program to identify candidate TSS in PROcap-seq and PRO-seq of flavopiridol-treated HFF (42, 81). For this purpose, iTiSS was run separately for each sample in the SPARSE_PEAK mode with standard parameters. Afterward, the iTiSS TSRMerger program was used to select only peaks that were identified in both samples within ±5 bp. Consistent peaks were only further considered if they were within 500 bp of the nearest annotated gene, and for each gene the TSS with the highest read count (weighted by the number of possible alignments for the read) was selected for further analyses.

### Calculation of pausing indices.

PRO-seq read counts in promoter windows (TSS to TSS + 250 bp) and gene bodies (TSS + 250 bp to TSS + 2,250 bp or gene 3′ end if closer) were determined using featureCounts (82) and gene annotations from Ensembl (version 87 for GRCh37) (83) in a strand-specific manner and normalized by the total number of reads and window lengths to obtain RPKM values. RPKM values were averaged between replicates and genes with zero reads in either promoter or gene body window were excluded from the analysis. PI for a gene was then calculated as the ratio of promoter RPKM to gene body RPKM.

### Metagene and clustering analysis.

Metagene analyses were performed as previously described (84) using the R program developed for this previous publication (available with the Watchdog *binGenome* module in the Watchdog module repository (https://github.com/watchdog-wms/watchdog-wms-modules/)). For promoter region analyses, the regions −3 kb to +3 kb of the TSS were divided into 101-bp bins for each gene. For each bin, the average coverage per genome position was calculated in a strand-specific manner for PRO-seq data and bin read coverages were then normalized by dividing by the total sum of all bins. Metagene curves for each replicate were created by averaging results for corresponding bins across all genes and metagene plots and then showing the average metagene curves across replicates. Genes without any reads in any of the analyzed samples were excluded from the analysis. For metagene analyses on the whole gene, the regions from −3 kb to +1.5 kb of the TSS and from −1.5 kb to +3 kb of the TTS were divided into 90-bp bins, and the remainder of the gene body (+1.5 kb of TSS to −1.5 kb of TTS) into 100 bins of variable length to compare genes with different lengths. Genes with a gene length <3 kb were excluded as regions around the TSS and TTS would overlap otherwise. To determine the statistical significance of differences between average metagene curves for two conditions, paired Wilcoxon signed rank tests were performed for each bin comparing normalized coverage values for each gene for this bin between the two conditions. *P* values were adjusted for multiple testing with the Bonferroni method across all bins within each subfigure and are color-coded in the bottom track of subfigures: red = adj. *P* value ≤ 10^−15^, orange = adj. *P* value ≤ 10^−10^, yellow = adj. *P* value ≤ 10^−3^.

For hierarchical clustering analysis, PRO-seq profiles for each gene and condition were calculated for sense or antisense strand as for metagene analyses (without averaging across genes). PRO-seq profiles in promoter windows for mock and WT-F infection at 3 h p.i. were then concatenated and divided by the maximum value in the concatenated vector. Hierarchical clustering was performed using the hclust function in R according to Euclidean distances and Ward’s clustering criterion. Peaks in metagene plots for each cluster were then determined in the following way: first, all local and global maxima and minima of metagene curves for each condition were identified for each cluster using the find_peaks function in the R ggpmisc package. The major peak was the global maximum. Subsequently, the next highest local maxima up- or downstream of the major peak were determined and retained as secondary peaks if (i) they were sufficiently removed from the borders of the 6 kb promoter window (i.e., within bins 30 to 80 of the 101 bins), (ii) the difference between the height of the secondary peak and the minimum value between the major and secondary peak was at least 10% of the major peak height, and (iii) the height of the secondary peak was at least 20% of the major peak height.

### Over- and underrepresentation analysis.

Over- and underrepresentation analysis of Gene Ontology (GO) terms and transcription factor binding motifs from TRANSFAC was performed for each cluster using the g:Profiler webserver (85) and the R package gprofiler2 (86), which provides an R interface to the webserver. *P* values were corrected for multiple testing using the Benjamini-Hochberg false discovery rate (87) and significant terms or motifs were identified at an adjusted *P* value cutoff of 0.001.

### Calculation of GC content and GC skew.

Genome sequences in the ±3kb around the TSS for each gene were extracted from the hg19 genome with twoBitToFa (http://genome.ucsc.edu/goldenPath/help/twoBit.html) and mean GC content and GC skew (G − C)/(G + C) was calculated in 100-bp sliding windows with steps of 1 bp as described by Watts et al. (39).

### Differential gene expression analysis and quantification of read-in transcription.

Number of fragments (=read pairs) per gene or in the 5 kb upstream of a gene were determined from mapped paired-end 4sU-seq reads in a strand-specific manner using featureCounts (82) and gene annotations from Ensembl (version 87 for GRCh37). For genes, all fragments overlapping exonic regions on the corresponding strand by ≥25bp were counted for the corresponding gene. For the 5-kb upstream regions, all fragments overlapping the 5 kb upstream of the gene 5′ end were counted. Fold changes in gene expression and statistical significance of changes were determined using DESeq2 (88), and *P* values were adjusted for multiple testing using the method by Benjamini and Hochberg (87). Gene expression and upstream transcriptional activity were quantified in terms of fragments per kilobase of exons per million mapped reads (FPKM). Only reads mapped to the human genome were counted for the total number of mapped reads for FPKM calculation. The percentage of read-in transcription was calculated as previously described (7, 46) for 7,271 genes that had no up- or downstream gene within 5 kb and were well expressed (average FPKM over replicates ≥1) in at least one time point of our 4sU-seq time course. For this purpose, the percentage of transcription upstream of a gene was first calculated separately for each replicate as percentage of upstream transcription = 100 × (FPKM in 5 kb upstream of gene)/(gene FPKM) and averaged between replicates. Second, the percentage of read-in at each 4sU-seq time point of infection was calculated as the percentage of upstream transcription in infected cells minus the percentage of downstream transcription in uninfected cells. Negative values were set to 0.

### Code availability.

Workflows for PI calculation, metagene analyses, clustering, and figure creation were implemented and run in Watchdog (71, 72) and are available at https://doi.org/10.5281/zenodo.7322848. Corresponding Watchdog modules are available in the Watchdog module repository (https://github.com/watchdog-wms/watchdog-wms-modules/).
