# Supplementary material for: HSV-1 Infection Induces a Downstream Shift of Promoter-Proximal Pausing for Host Genes
Source: J Virol. 2023 Apr 24;97(5):e00381-23. doi: 10.1128/jvi.00381-23 (PMC10231138; doi:10.1128/jvi.00381-23)
Supplement: Supplemental file 3 — Fig. S1 to S16. Download jvi.00381-23-s0003.pdf, PDF file, 7.2 MB [file jvi.00381-23-s0003.pdf]

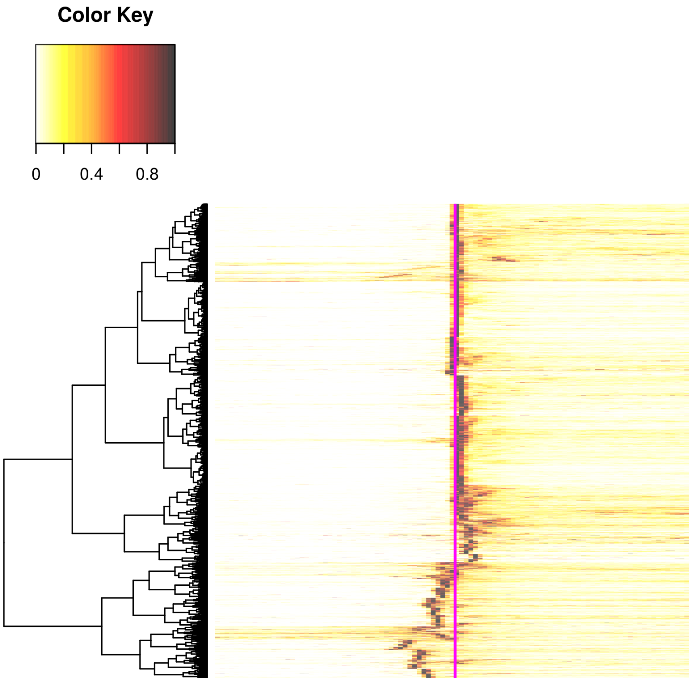

(a)

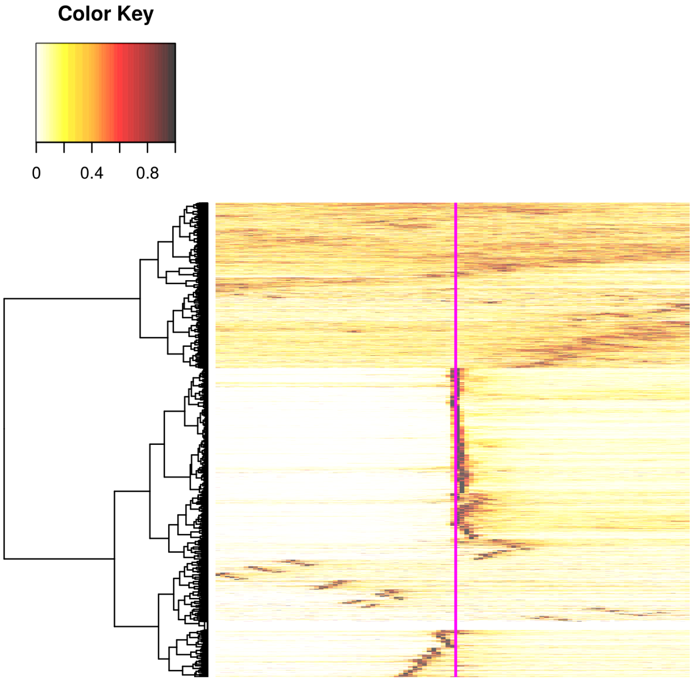

(b)

Fig. S1 Heatmaps of PRO-seq profiles on the sense strand in mock infection in a window of ± 3 kb around **(a)** the TSS positions identified from PROcap-seq and PRO-seq data of flavopiridol-treated HFF or **(b)** annotated gene 5' ends. For this purpose, PRO-seq profiles were divided by the maximum value in the ± 3 kb promoter window, resulting in a value of 1 for the position of the highest peak in PRO-seq profiles. Hierarchical clustering of normalized PRO-seq profiles for all genes was performed using the *hclust* function in R according to Euclidean distances and Ward's clustering criterion. The central position in the promoter window (= the identified TSS) is marked by a vertical magenta line.

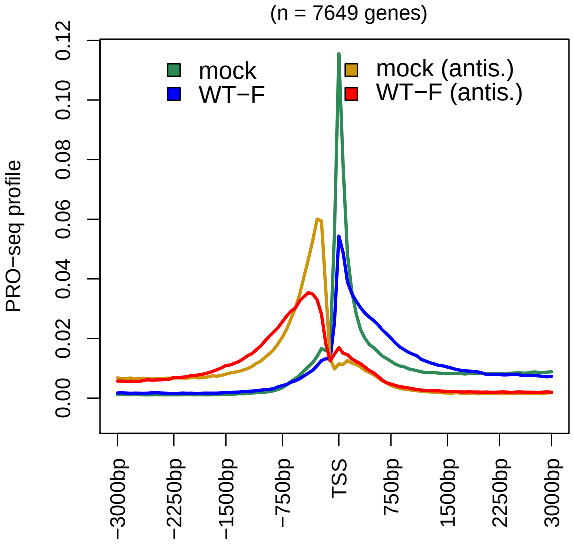

(a)

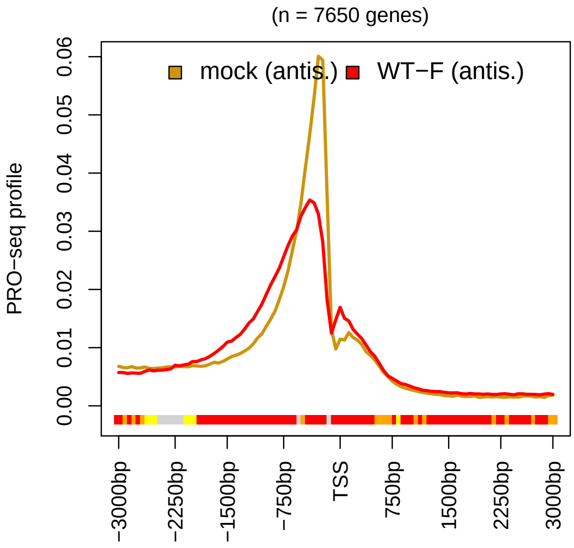

(b)

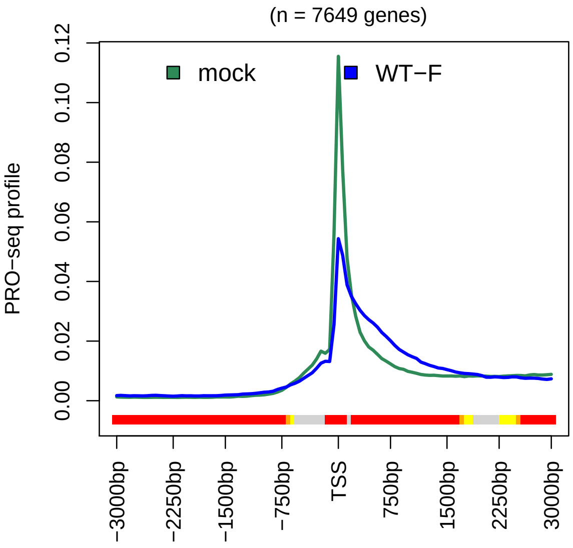

(c)

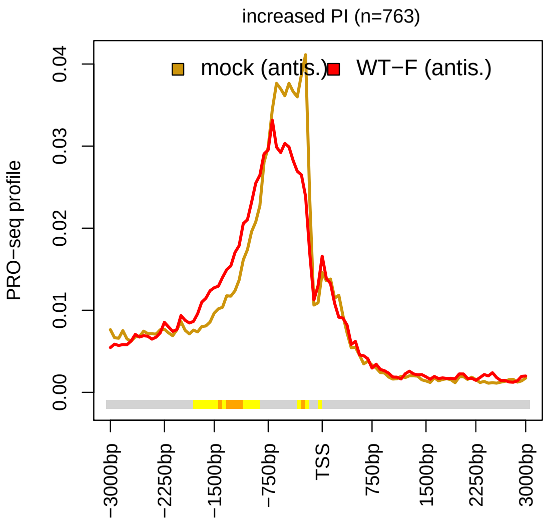

(d)

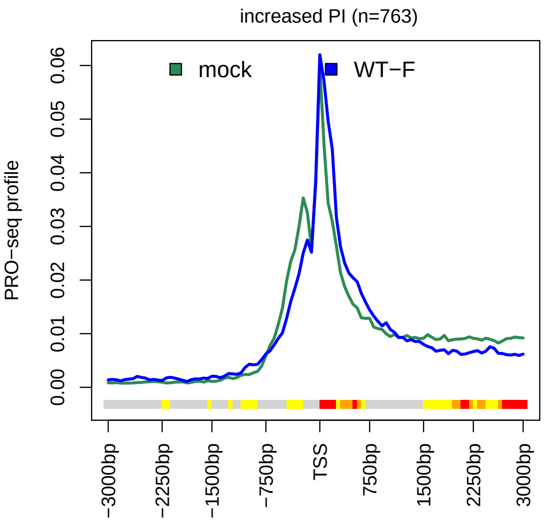

(e)

(Continued on next page)

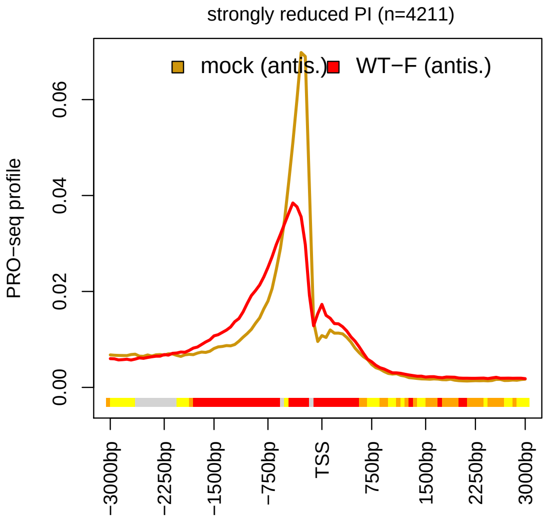

(f)

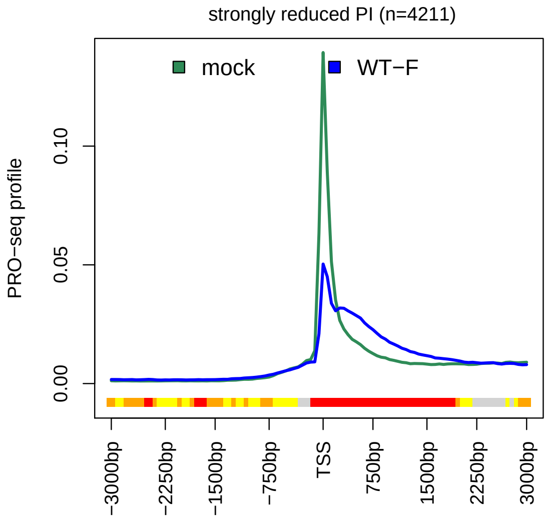

(g)

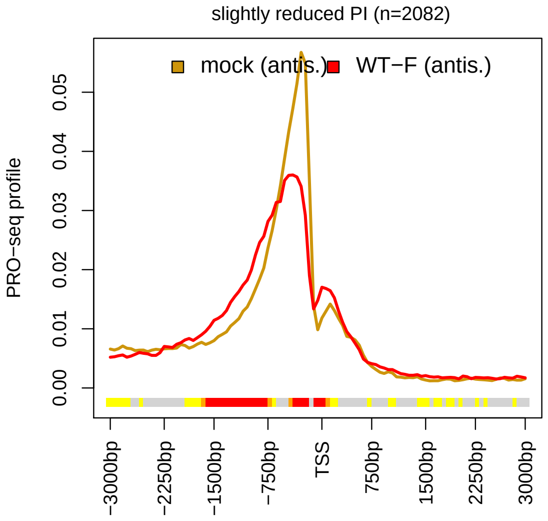

(h)

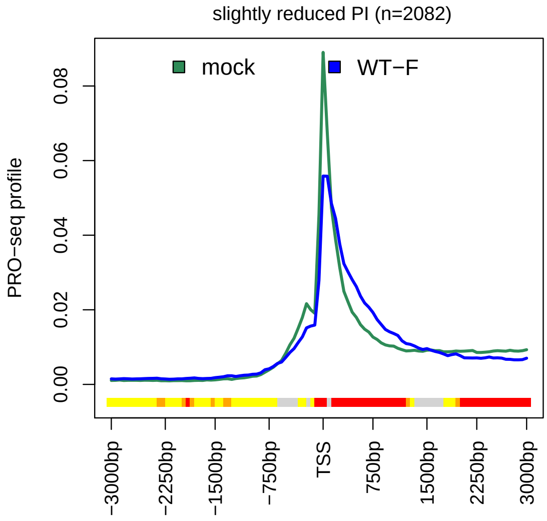

(i)

Fig. S2 (a-c) Metagene plot showing the distribution of PRO-seq profiles in sense (dark green and blue) and antisense (gold and red) direction from -3 kb to +3 kb around the TSS for all analyzed genes for mock infection (dark green and gold) and WT-F 3 h p.i. infection (dark blue and red). One gene without reads on the sense strand in some of the analyzed samples was excluded. **(b)** and **(c)** show metagene curves from **(a)** separately for antisense **(b)** and sense **(c)** direction. The color track at the bottom indicates the significance of paired Wilcoxon tests comparing the normalized PRO-seq coverages of genes for each bin between mock and WT-F 3 h p.i. infection. P-values are adjusted for multiple testing with the Bonferroni method within each subfigure; color code: red = adj. p-value $\leq 10^{-15}$, orange = adj. p-value $\leq 10^{-10}$, yellow = adj. p-value $\leq 10^{-3}$. **(d-i)** Metagene plots showing the distribution of PRO-seq profiles separately for antisense **(d,f,h)** and sense **(e,g,i)** direction for genes with increased PI **(d,e)**, strongly reduced PI **(f,g)** and slightly reduced PI **(h,i)**. The color track at the bottom indicates the significance of paired Wilcoxon tests comparing the normalized PRO-seq coverages of genes for each bin between mock and WT-F 3 h p.i. infection.

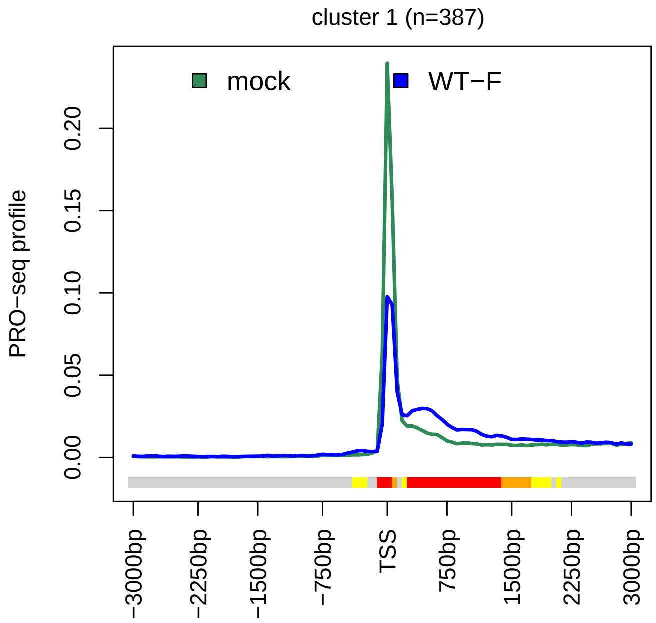

(a)

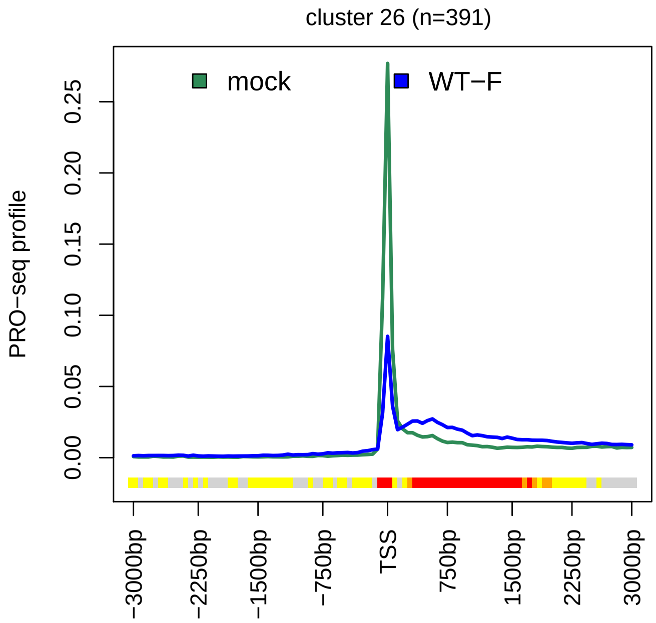

(b)

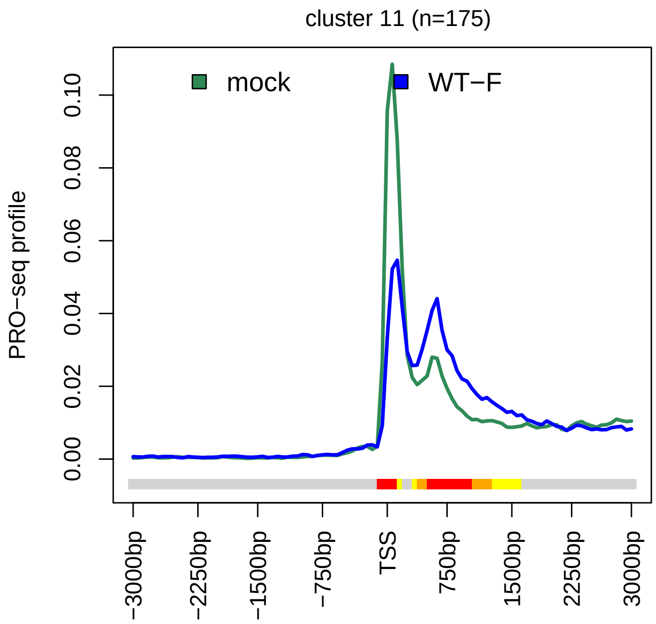

(c)

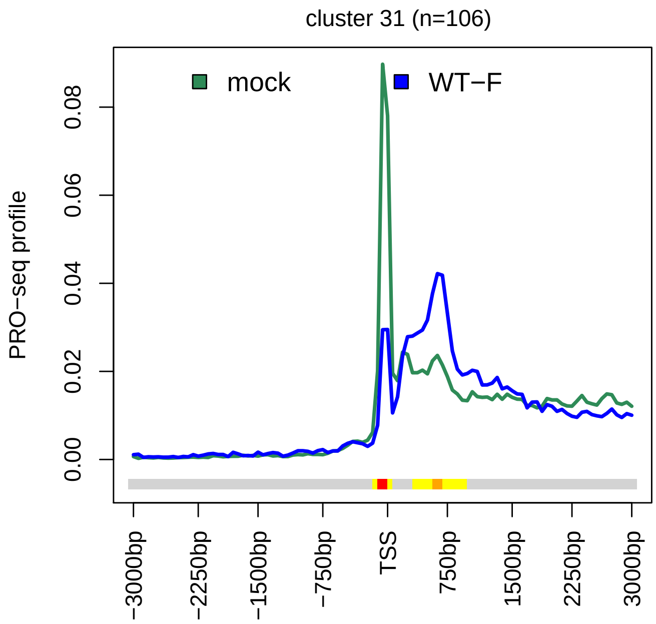

(d)

(Continued on next page)

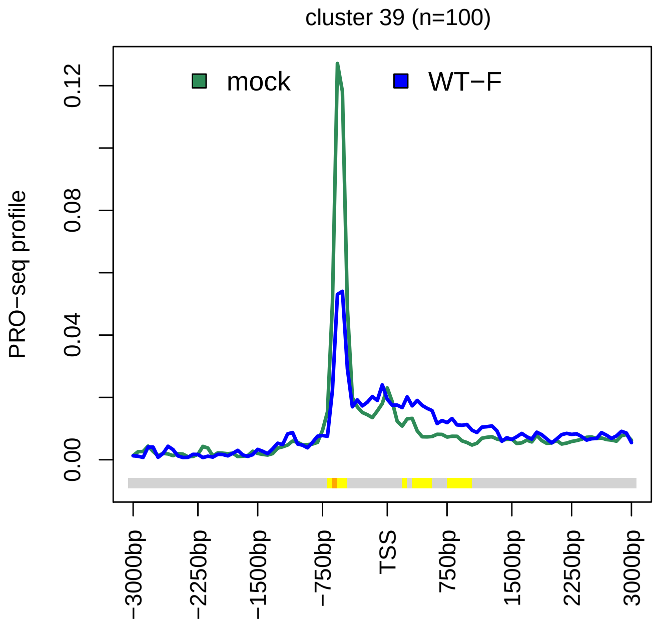

(e)

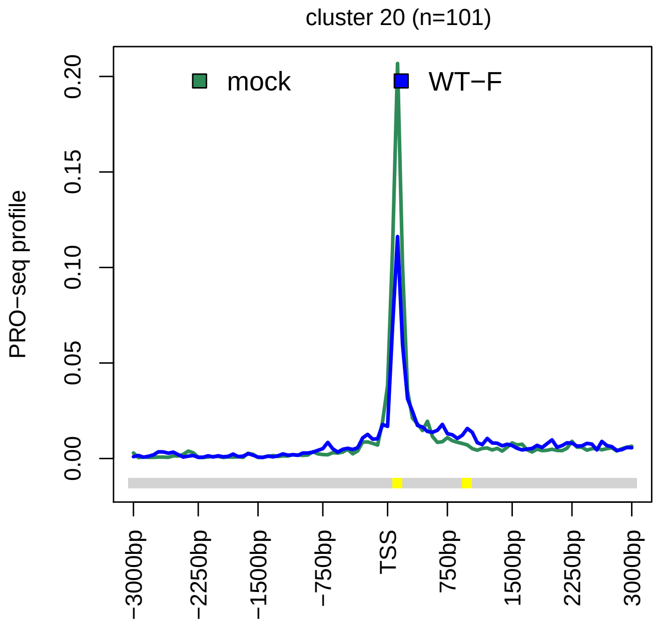

(f)

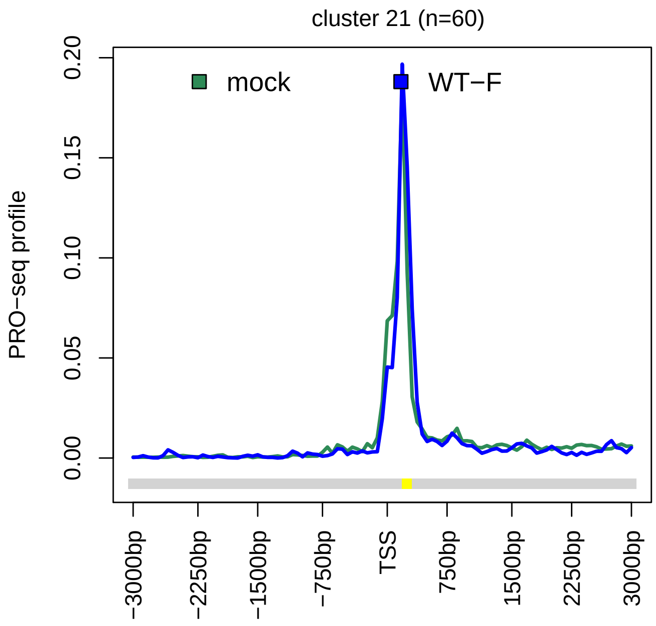

(g)

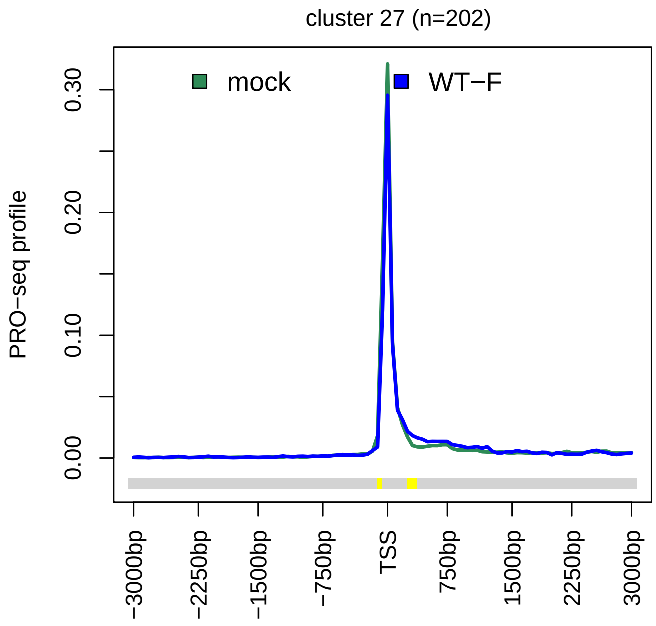

(h)

Fig. S3 Metagene plots showing the PRO-seq profile in sense direction from -3 kb to +3 kb around the TSS for mock infection (dark green) and WT-F 3 h p.i. infection (dark blue) separately for example clusters. Cluster numbers and number of genes in each cluster are indicated on top of subfigures. The color track at the bottom of each subfigure indicates the significance of paired Wilcoxon tests comparing the normalized PRO-seq coverages of genes for each bin between mock and WT-F 3 h p.i. infection. P-values are adjusted for multiple testing with the Bonferroni method within each subfigure; color code: red = adj. p-value $\leq 10^{-15}$, orange = adj. p-value $\leq 10^{-10}$, yellow = adj. p-value $\leq 10^{-3}$.

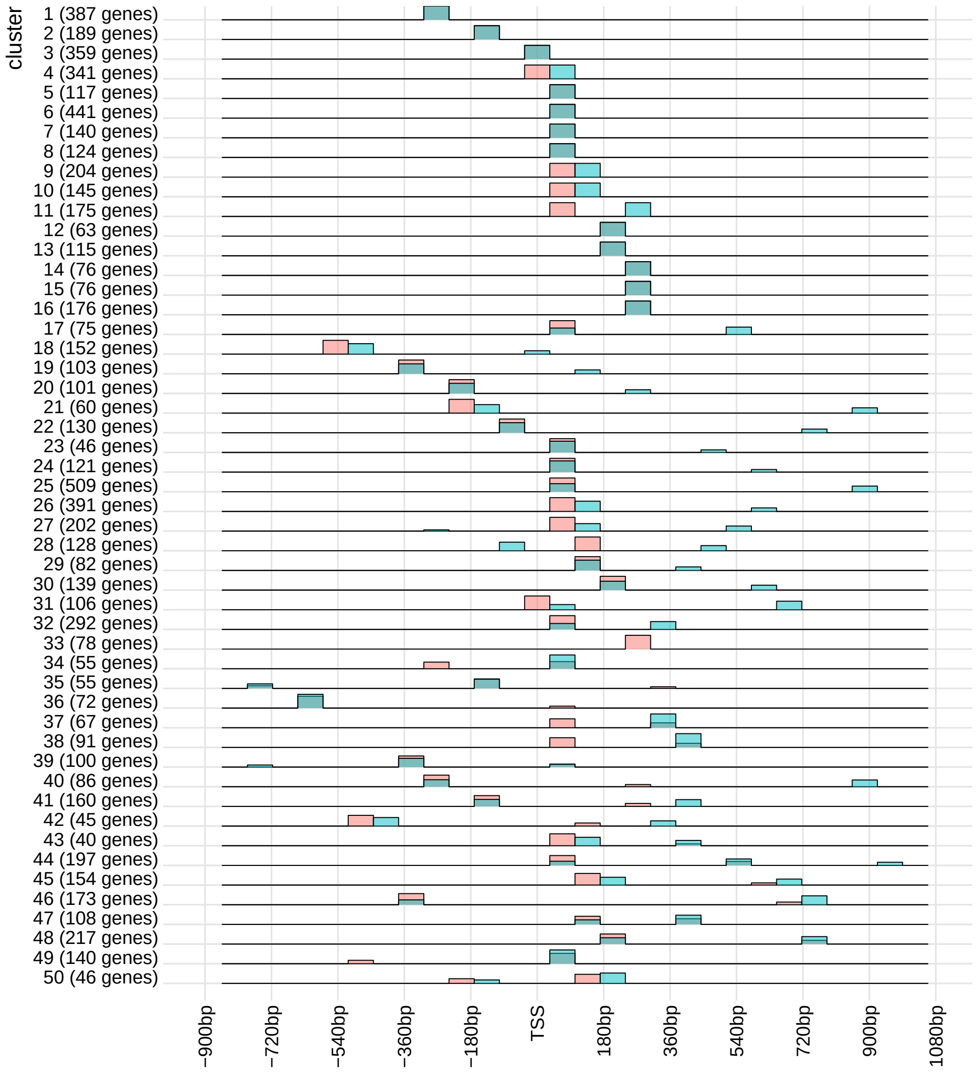

(a)

Peak pattern	mock no. clusters	mock no. genes	WT-F 3 h p.i. no. clusters	WT-F 3 h p.i. no. genes
One TSS peak	33	5728	21	3347
Additional minor peak downstream of major TSS peak	13	1461	17	2986
Two approximately equally high peaks	2	232	4	296
Additional downstream peak higher than TSS peak	0	0	7	834
none of the above	2	228	1	46

(b)

Fig. S4 (a) Positions, number, and relative heights of peaks identified in PRO-seq profiles in sense direction for the 50 clusters. Mock infection is shown in light red and WT-F 3 h p.i. infection in turquoise. Darker turquoise indicates that a peak is present at the same position in mock and WT-F 3 h p.i. infection. The relative peak height is calculated as the peak height divided by the sum of all peak heights for the same condition. Thus, a single peak has a value of 1, two equally high peaks both have a value of 0.5, and so on. **(b)** Statistics on the number of clusters and number of genes with different types of peak patterns defined by the number and relative height of peaks for mock and WT-F 3 h p.i. infection shown in **(a)**.

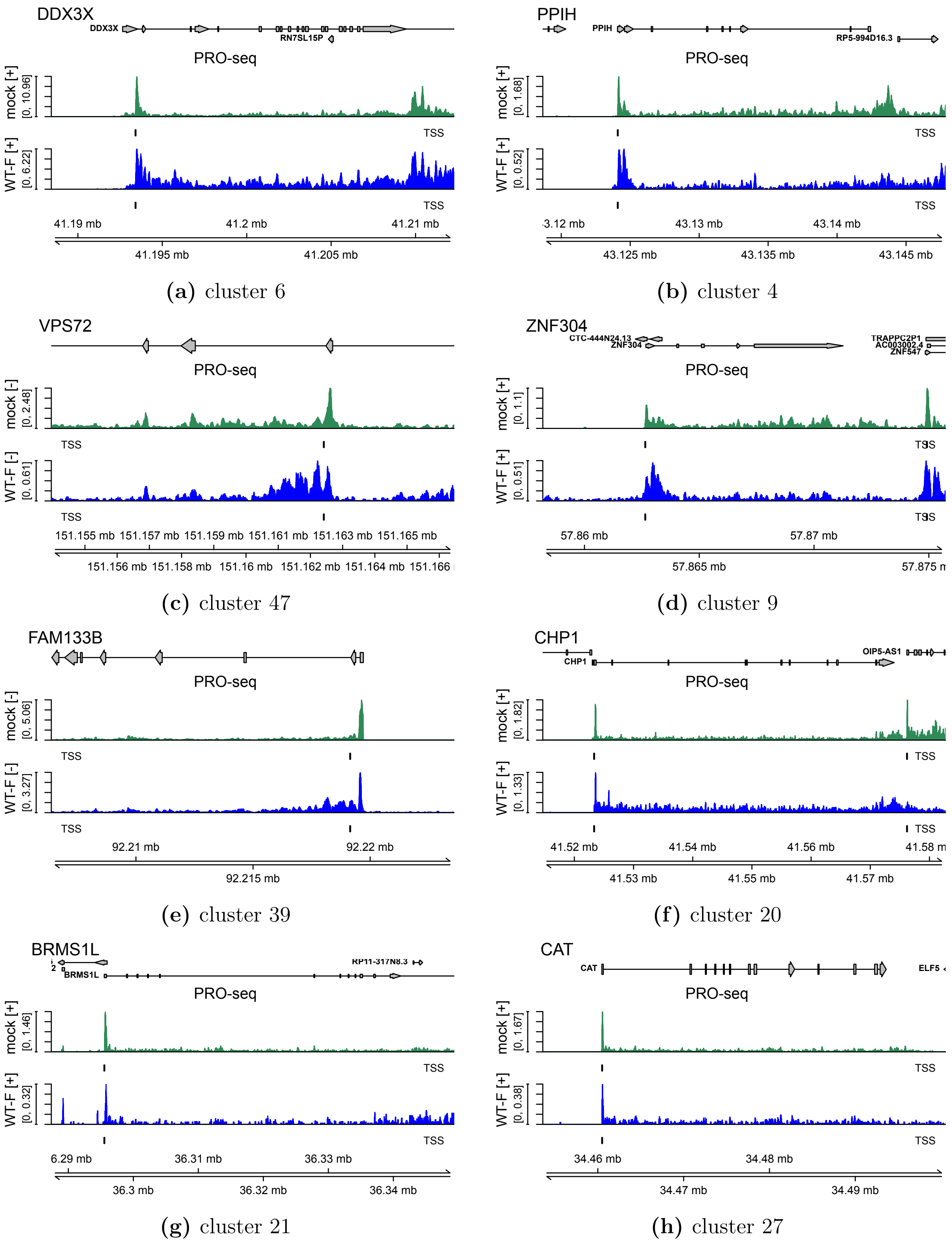

(a) cluster 6 (b) cluster 4 (c) cluster 47 (d) cluster 9 (e) cluster 39 (f) cluster 20 (g) cluster 21 (h) cluster 27

Fig. S5 Read coverage around the TSS in PRO-Seq data (sense strand only) for mock (green) and WT-F infection (blue) at 3 h p.i. for example genes (gene name of the selected gene on the top left) in different clusters (cluster number shown below subfigures). Read coverage was normalized to total number of mapped reads and averaged between replicates. The identified TSS used in the analysis is indicated by a short vertical line below each read coverage track. Gene annotation is indicated at the top. Boxes represent exons, lines represent introns and direction is indicated by arrowheads. Genomic coordinates are shown on the bottom. Please note that figures are not centered around the TSS, but a larger region downstream of the TSS was included than upstream of the TSS.

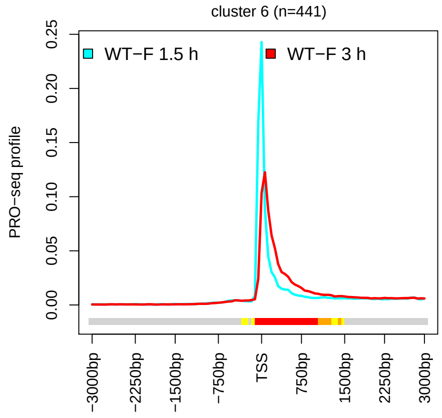

(a)

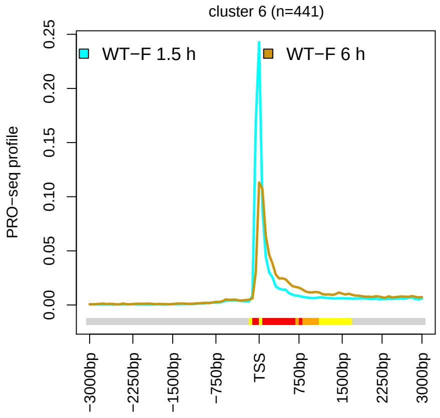

(b)

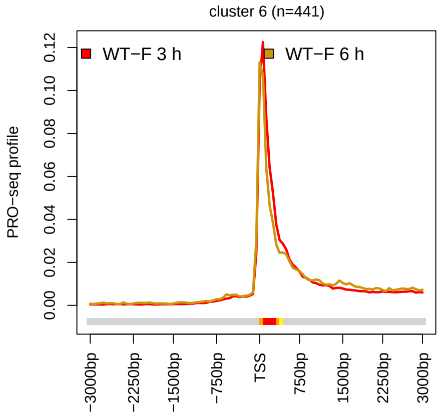

(c)

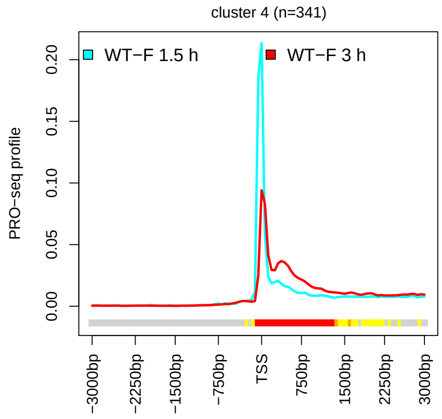

(d)

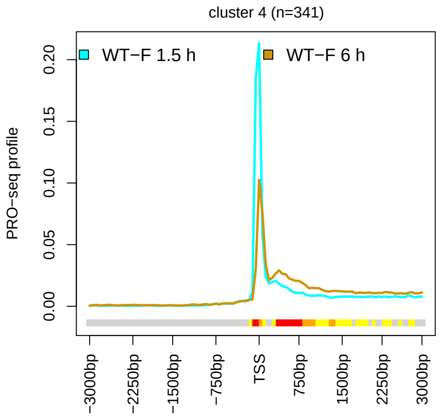

(e)

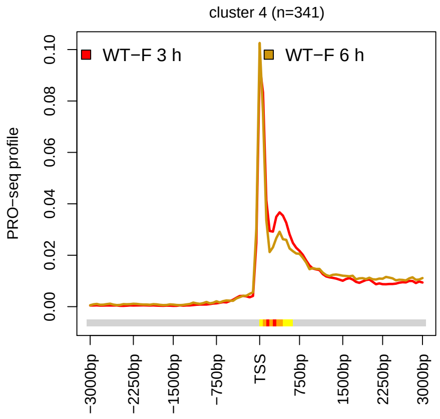

(f)

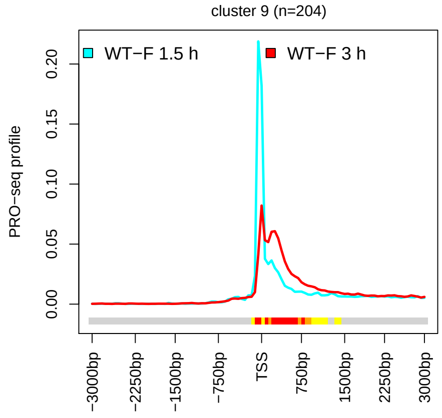

(g)

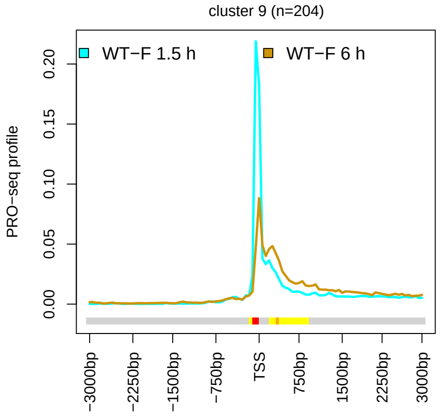

(h)

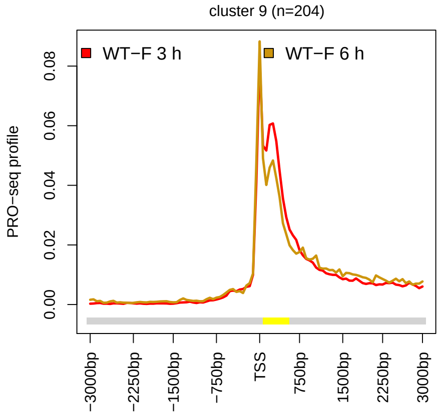

(i)

(Continued on next page)

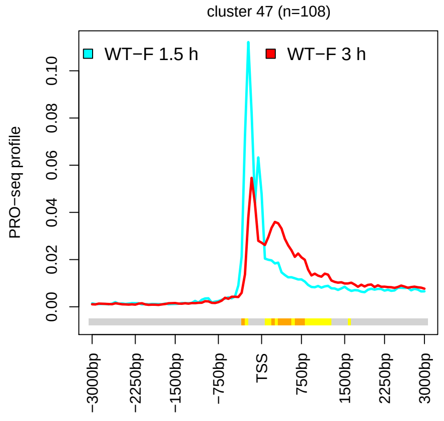

(j)

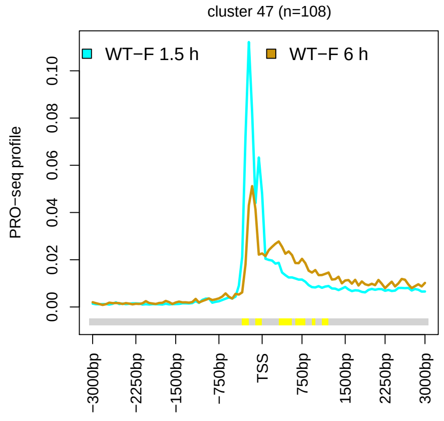

(k)

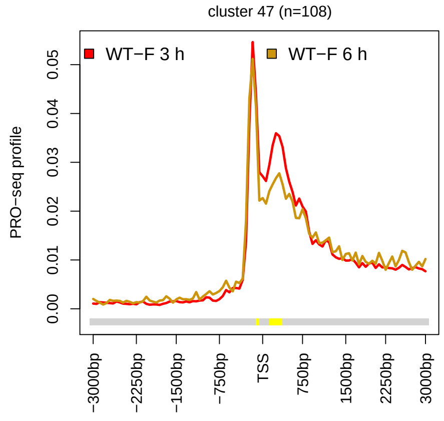

(l)

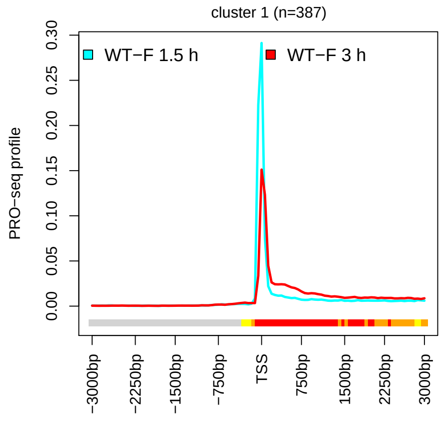

(m)

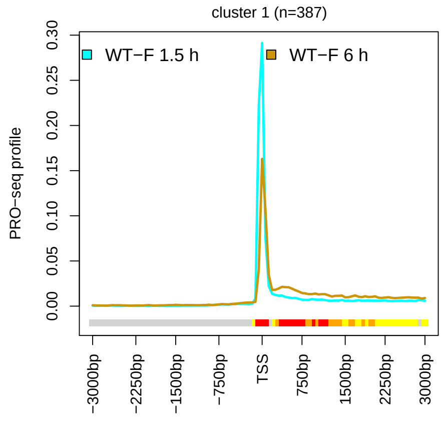

(n)

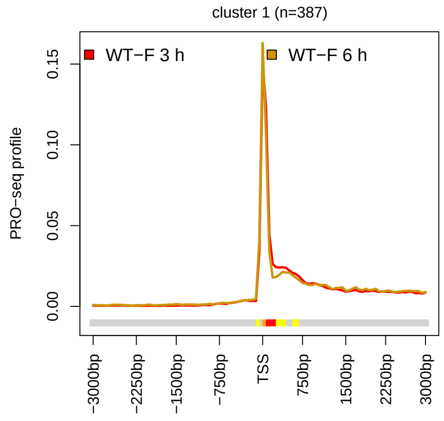

(o)

Fig. S6 Metagene plots showing the PRO-seq profile in sense direction from -3 kb to +3 kb around the TSS for the pairwise comparisons of WT-F 1.5, 3 and 6 h p.i. infection for example clusters. Cluster numbers and number of genes in each cluster are indicated on top of subfigures. The color track at the bottom of each subfigure indicates the significance of paired Wilcoxon tests comparing the normalized PRO-seq coverages of genes for each bin between the two time-points of WT-F infection. P-values are adjusted for multiple testing with the Bonferroni method within each subfigure; color code: red = adj. p-value $\leq 10^{-15}$, orange = adj. p-value $\leq 10^{-10}$, yellow = adj. p-value $\leq 10^{-3}$.

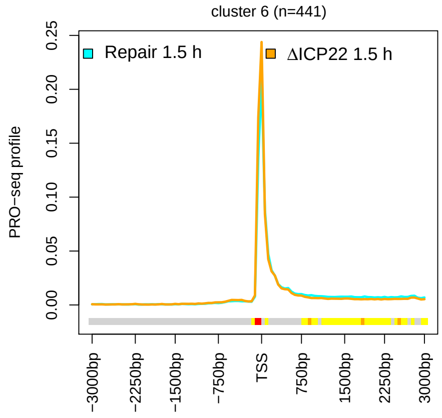

(a)

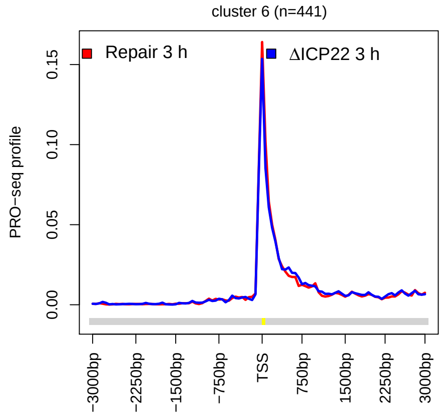

(b)

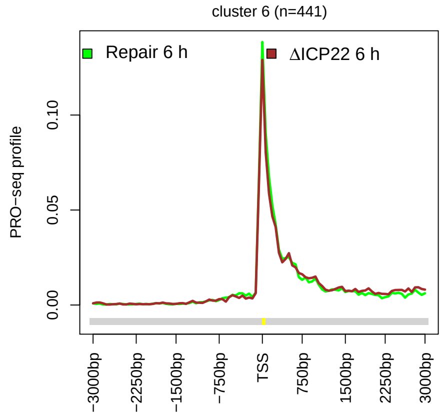

(c)

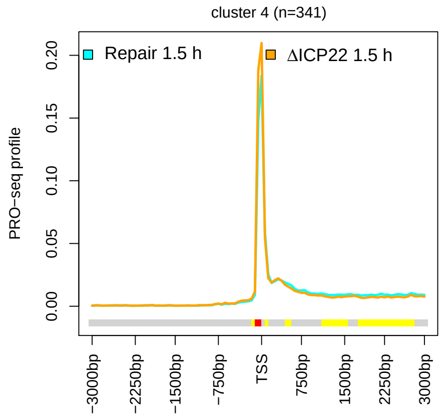

(d)

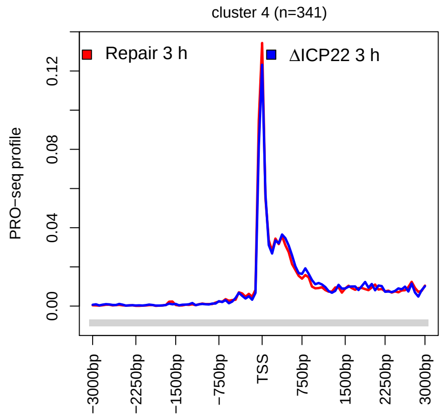

(e)

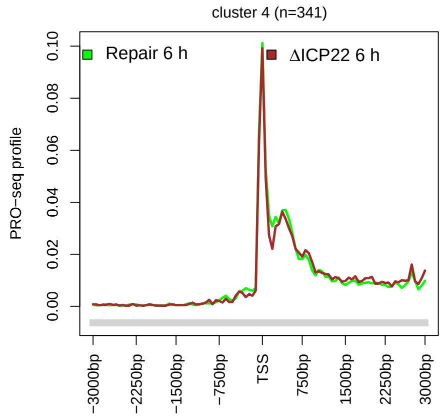

(f)

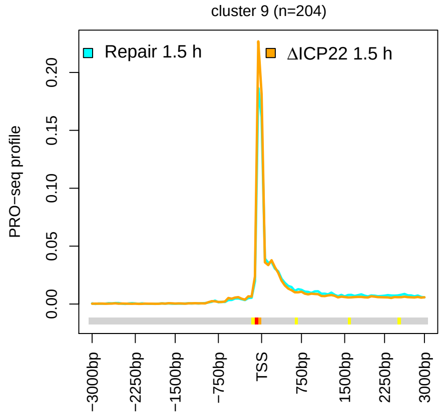

(g)

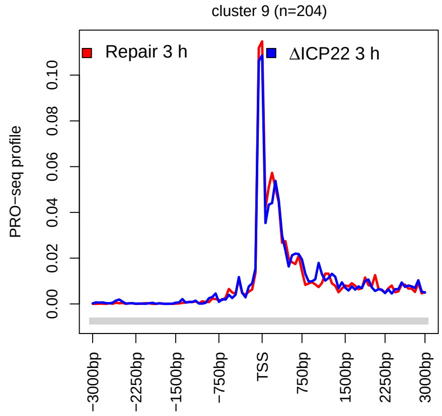

(h)

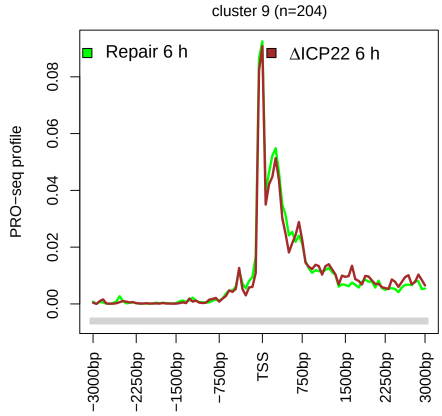

(i)

(Continued on next page)

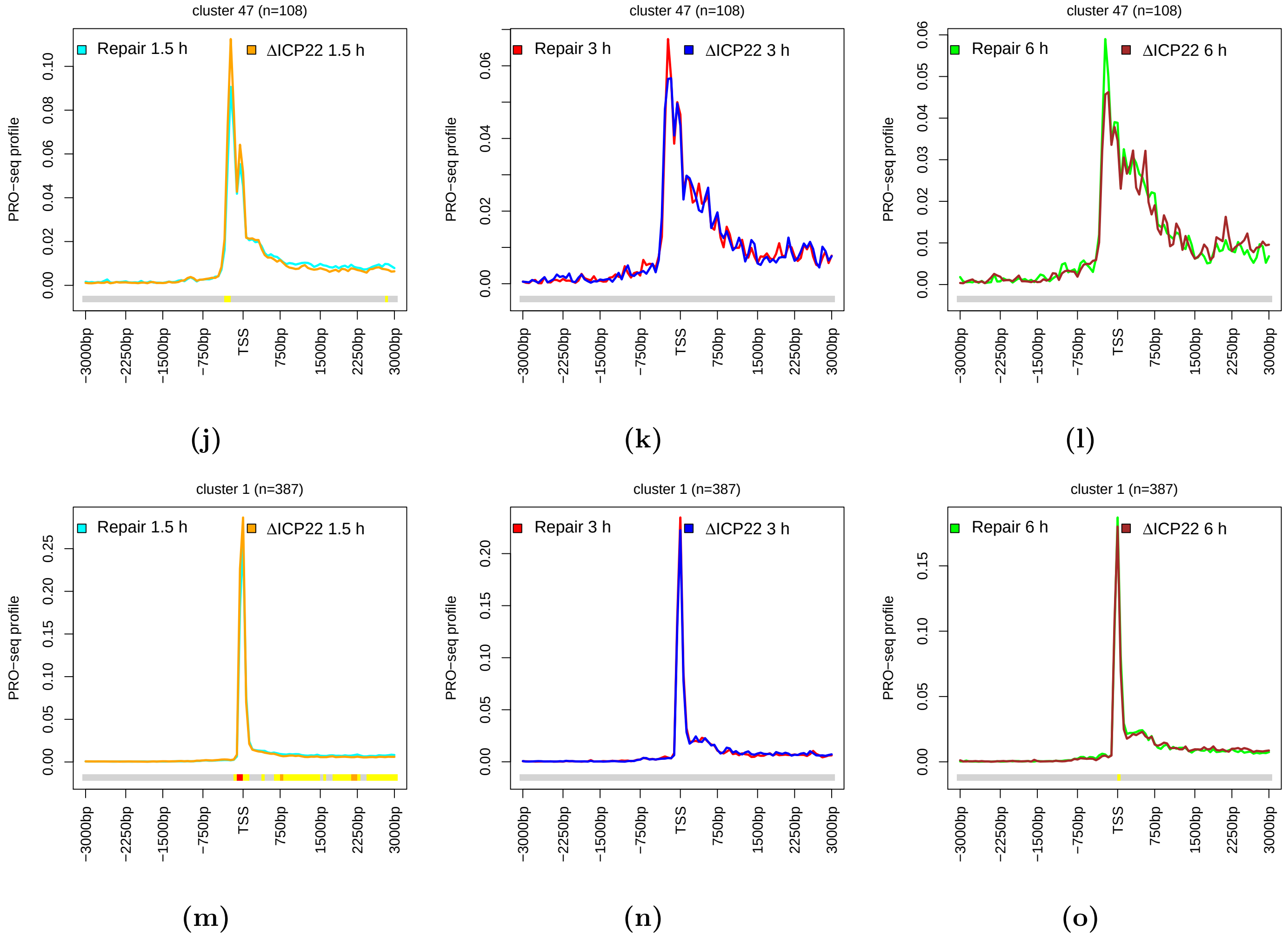

Fig. S7 Metagene plots showing the PRO-seq profile in sense direction from -3 kb to +3 kb around the TSS for the pairwise comparisons of ΔICP22 and repair virus infection at 1.5 (left column), 3 (middle column) and 6 h (right column) for example clusters. Cluster numbers and number of genes in each cluster are indicated on top of subfigures. The color track at the bottom of each subfigure indicates the significance of paired Wilcoxon tests comparing the normalized PRO-seq coverages of genes for each bin between the two time-points of WT-F infection. P-values are adjusted for multiple testing with the Bonferroni method within each subfigure; color code: red = adj. p-value $\leq 10^{-15}$, orange = adj. p-value $\leq 10^{-10}$, yellow = adj. p-value $\leq 10^{-3}$.

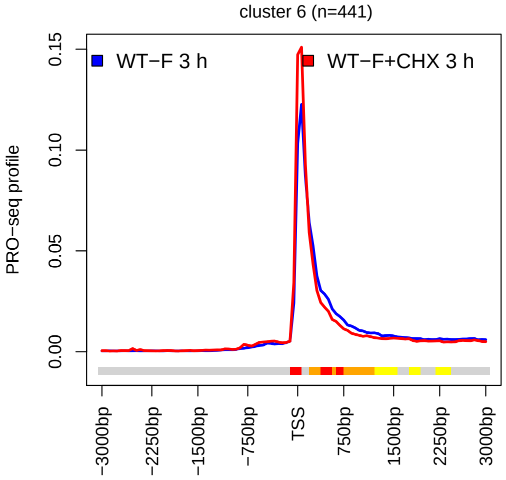

(a)

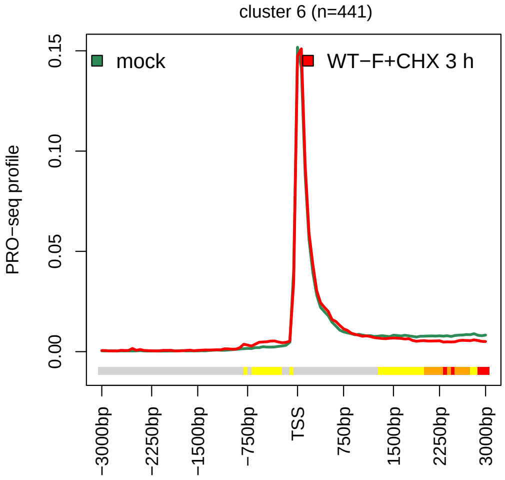

(b)

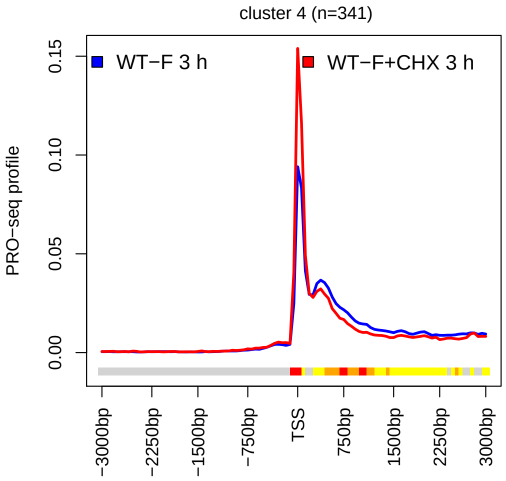

(c)

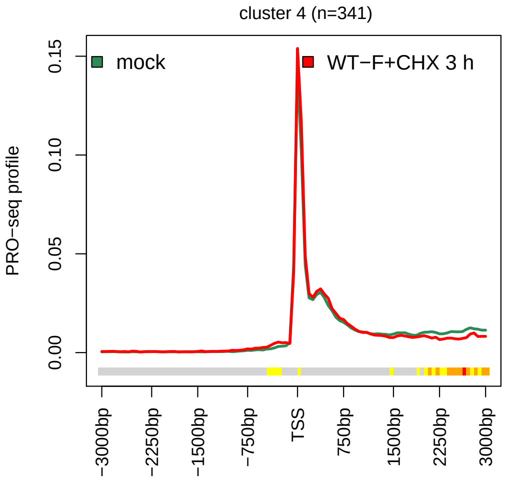

(d)

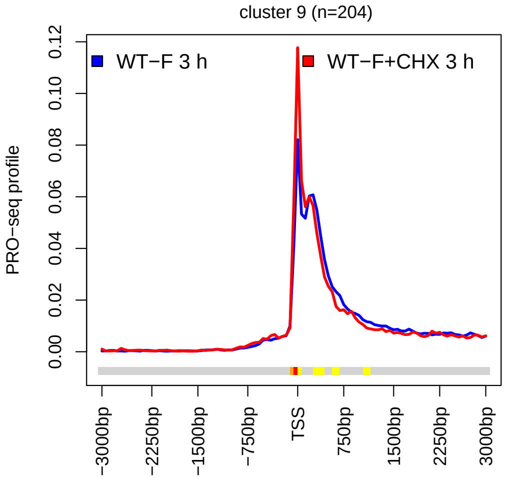

(e)

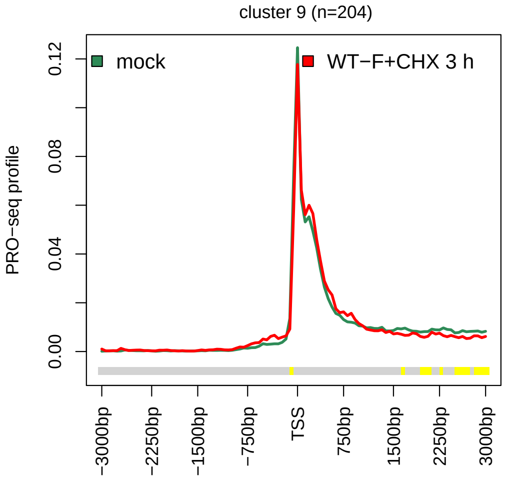

(f)

(Continued on next page)

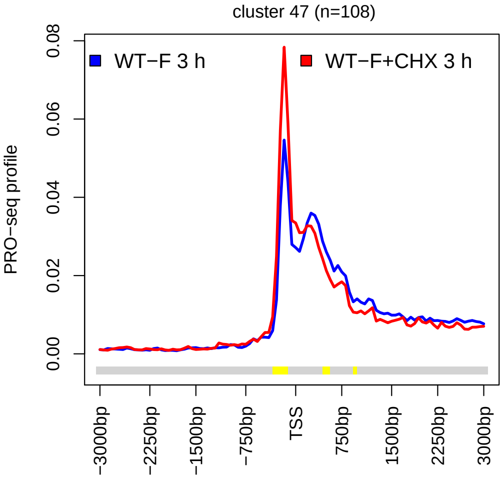

(g)

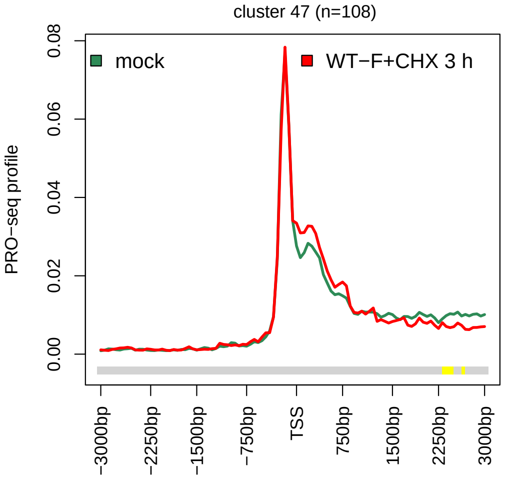

(h)

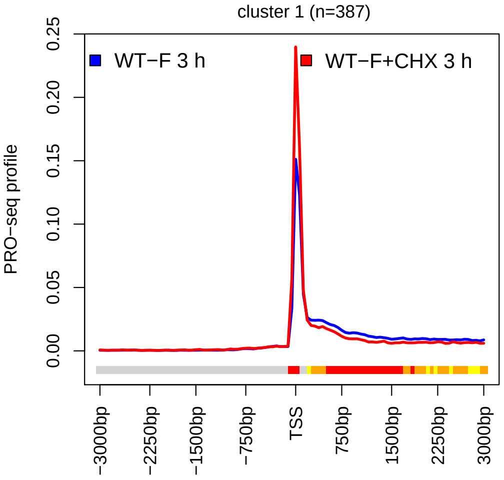

(i)

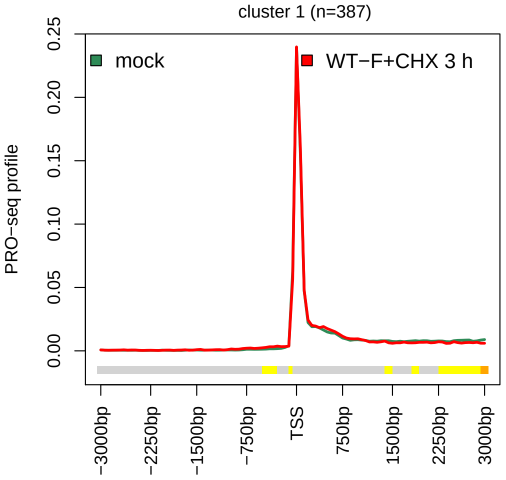

(j)

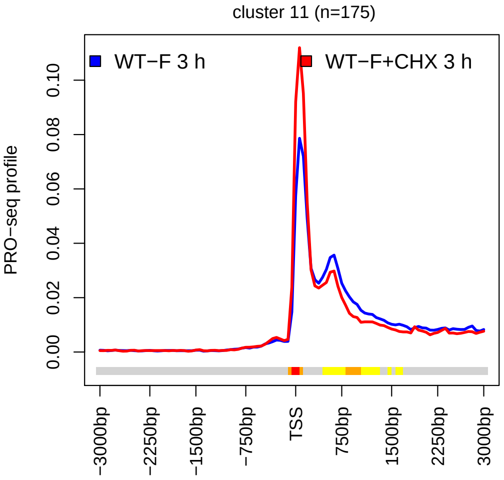

(k)

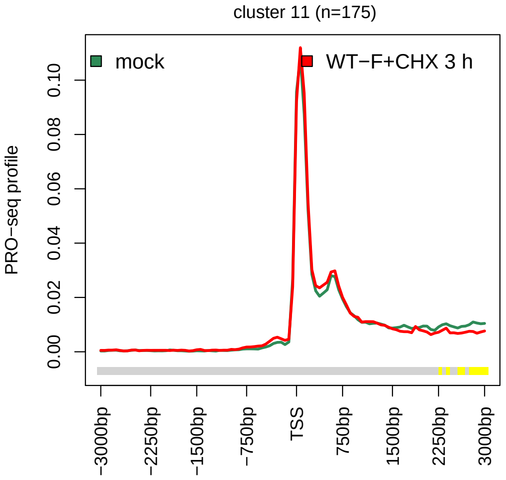

(l)

Fig. S8 Metagene plots showing the PRO-seq profile in sense direction from -3 kb to +3 kb around the TSS for WT-F 3 h p.i. ± CHX (left column) and for mock infection and WT-F 3 h p.i.+CHX (right column) for example clusters. Cluster numbers and number of genes in each cluster are indicated in subfigures. The color track at the bottom of subfigures indicates the significance of paired Wilcoxon tests comparing the normalized PRO-seq coverages of genes for each bin between the two conditions. P-values are adjusted for multiple testing with the Bonferroni method within each subfigure; color code: red = adj. p-value $\leq 10^{-15}$, orange = adj. p-value $\leq 10^{-10}$, yellow = adj. p-value $\leq 10^{-3}$.

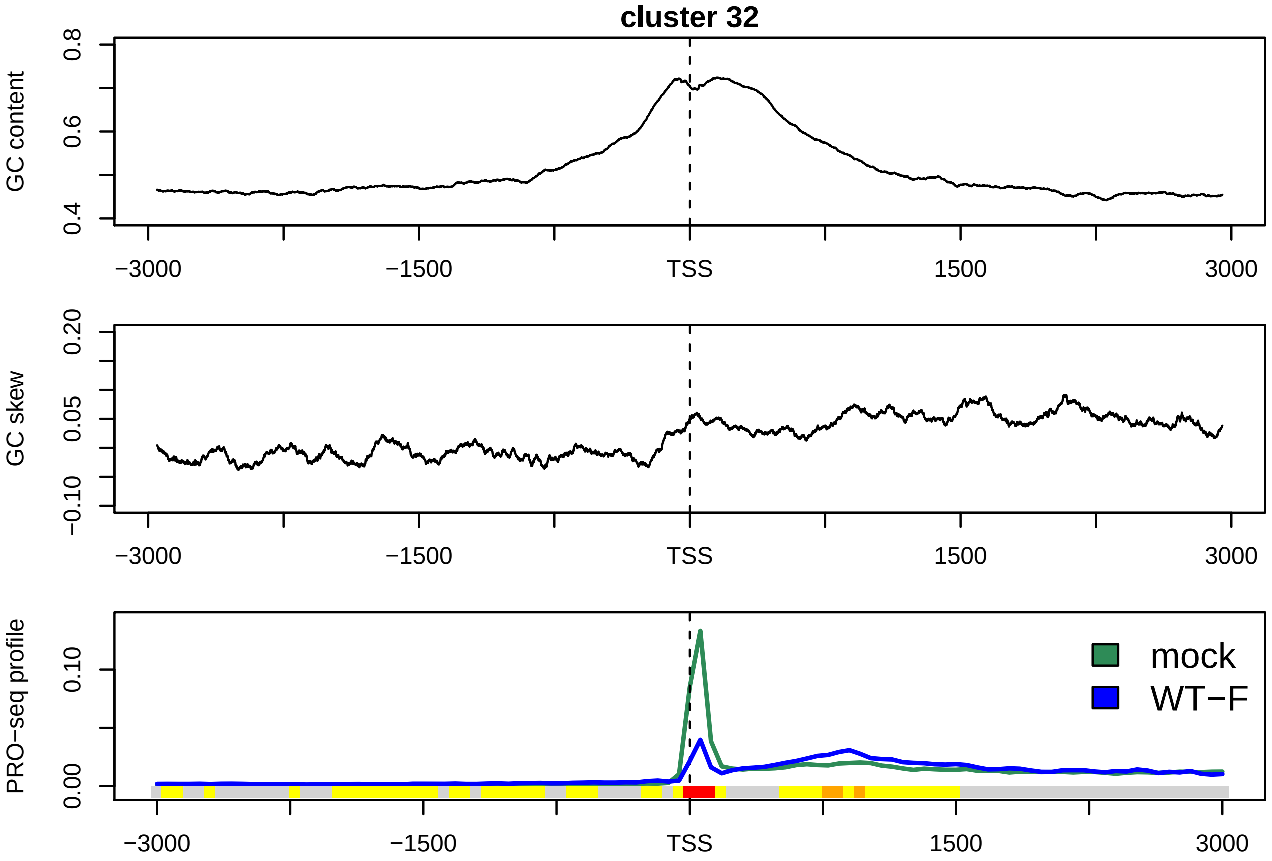

(a)

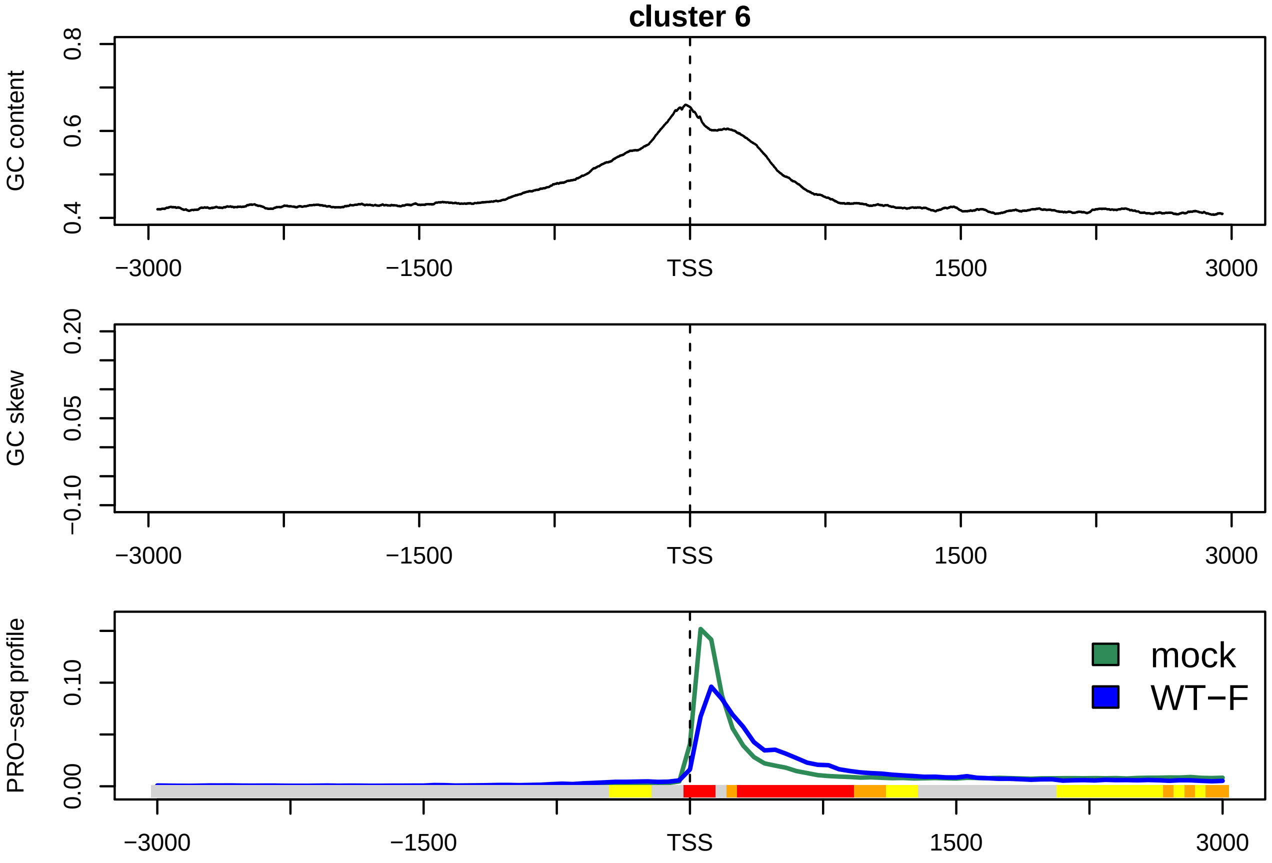

(b)

(Continued on next page)

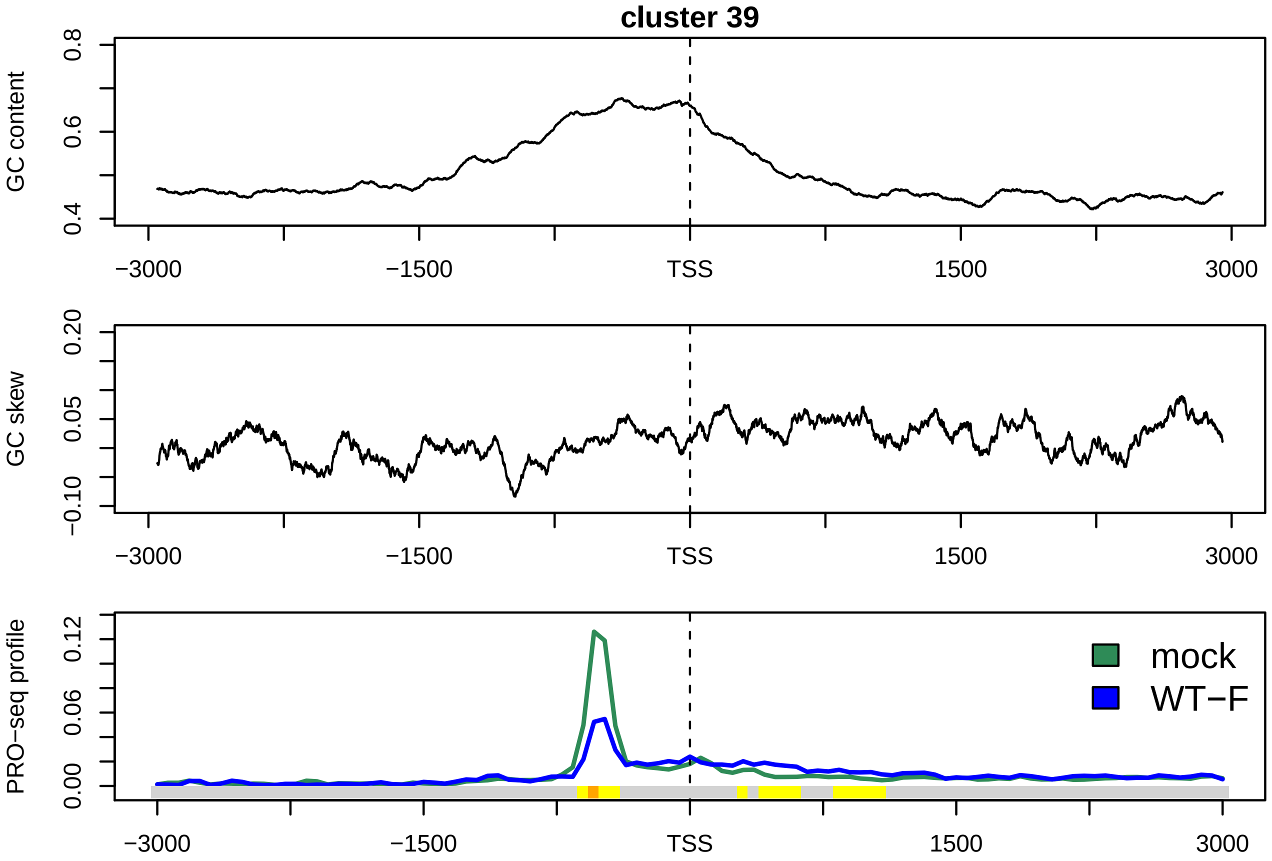

(c)

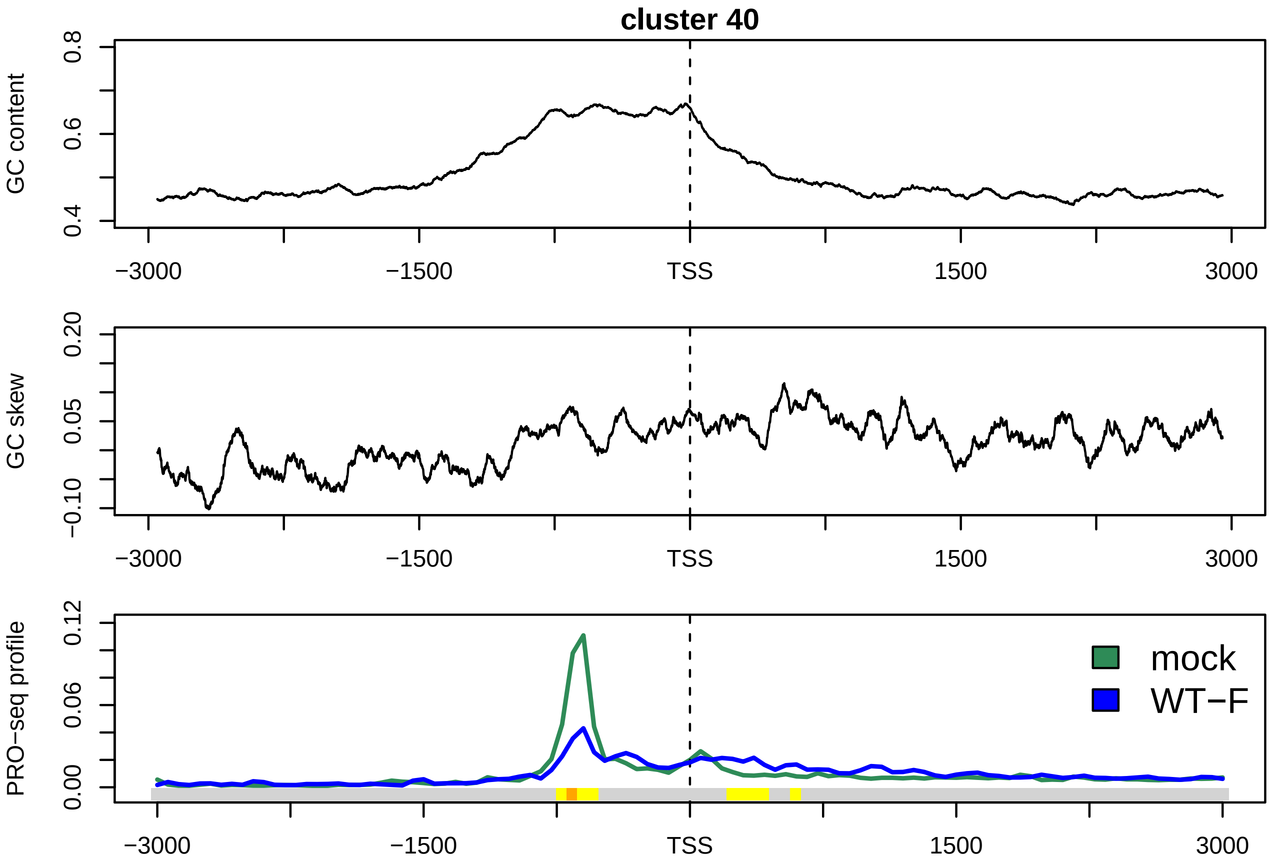

(d)

(Continued on next page)

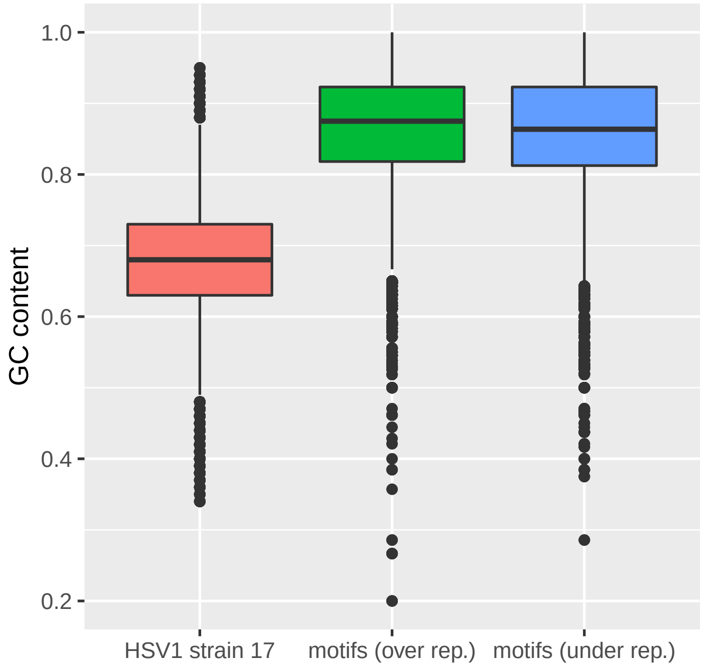

(e)

Fig. S9 (a-d) GC content and GC skew in promoter regions for example clusters (cluster numbers shown on top of subfigures). For each gene GC content and GC skew was determined in 100 bp sliding windows from -3 kb of the TSS to +3 kb of the TSS. Values for each sliding window were then averaged across genes in this cluster. The bottom panel of each subfigure shows the PRO-seq profiles in mock and WT-F 3 h p.i. infection for comparison. The color tracks at the bottom of PRO-seq panels indicate the significance of paired Wilcoxon tests comparing the normalized PRO-seq coverages of genes for each bin between the two time-points of WT-F infection. P-values are adjusted for multiple testing with the Bonferroni method within each subfigure; color code: red = adj. p-value $\leq 10^{-15}$, orange = adj. p-value $\leq 10^{-10}$, yellow = adj. p-value $\leq 10^{-3}$. **(e)** Boxplots showing the distribution of the GC content determined in sliding windows of length 100 bp on the HSV-1 genome (GenBank accession JN555585.1, red) and the GC content of motif occurrences in the HSV-1 genome for transcription factor motifs found to be either over-represented (green) or under-represented (blue) in Clusters 6 and 32. Motif occurrences were determined using the fimo function of the MEME suite.

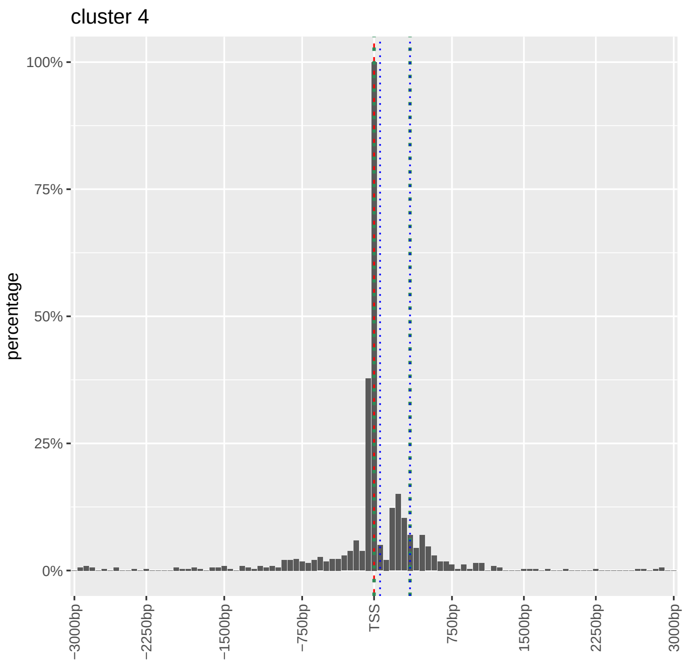

(a)

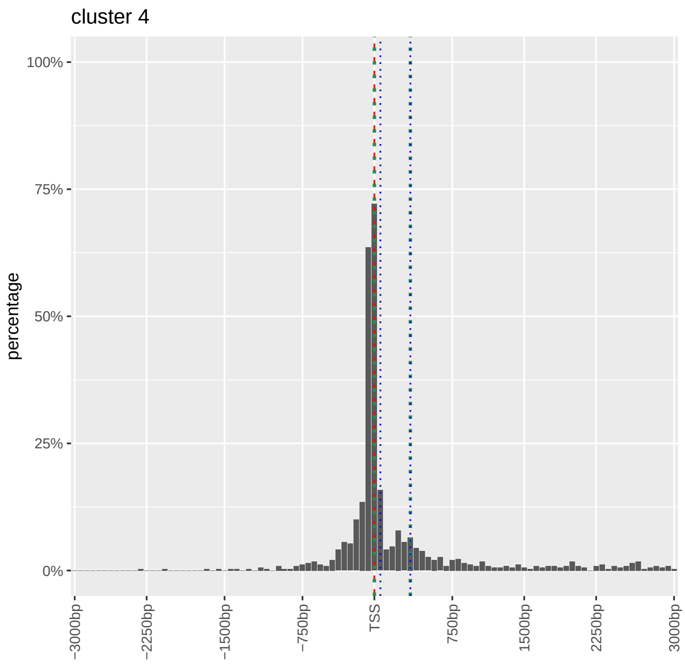

(b)

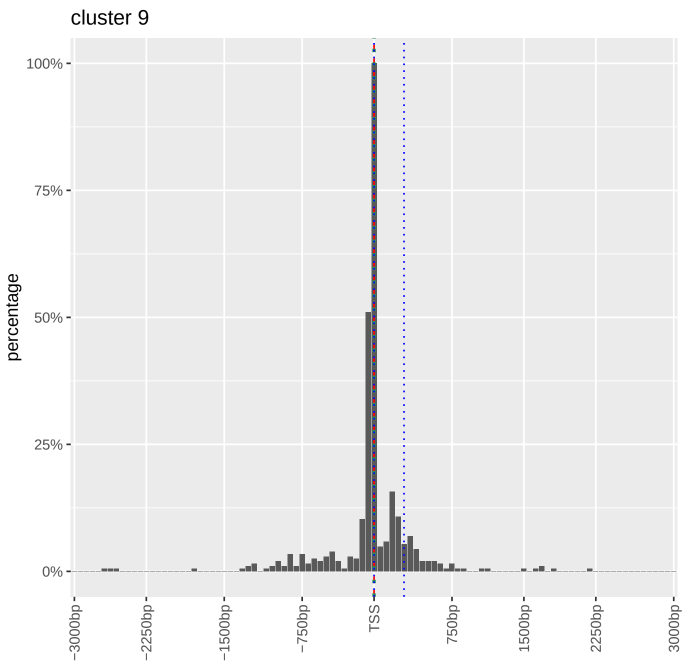

(c)

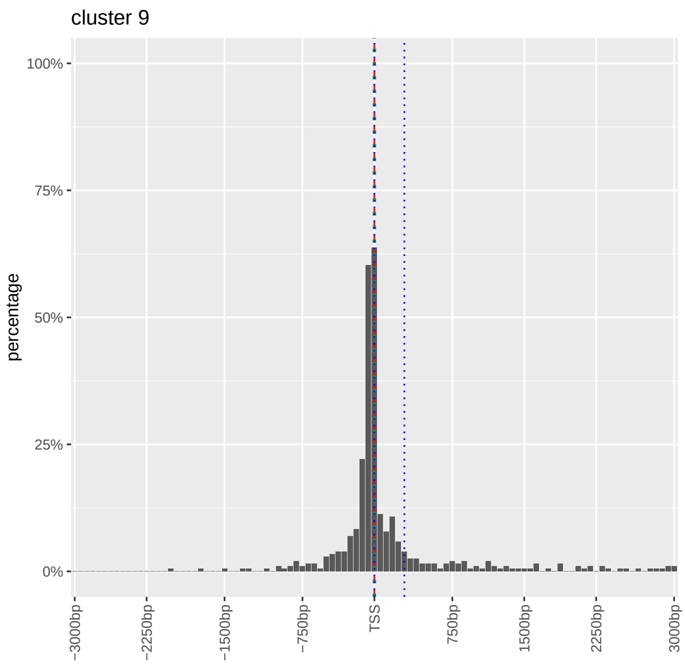

(d)

(Continued on next page)

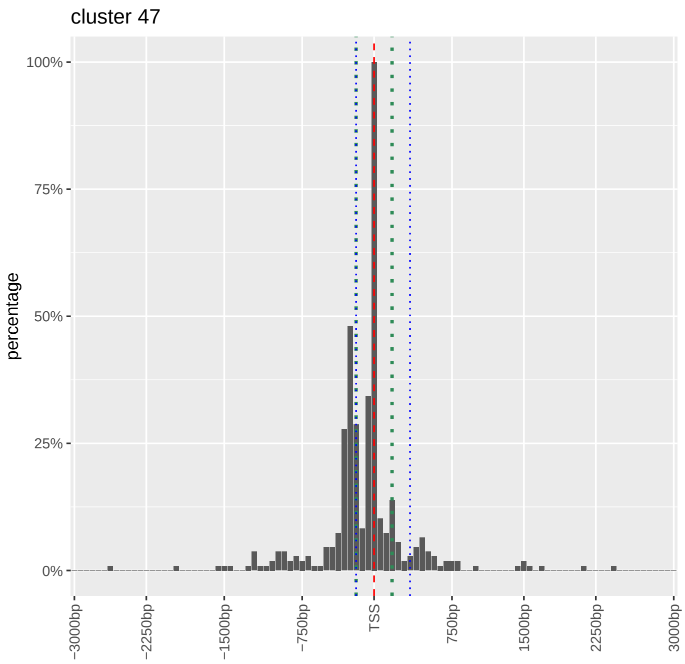

(e)

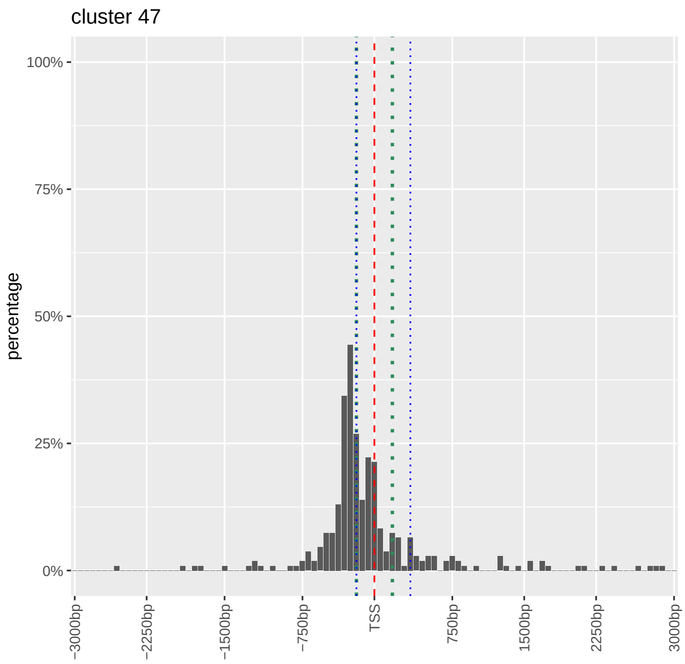

(f)

Fig. S10 (a,c,e) Percentage of genes exhibiting a peak in the PROcap-seq and PRO-seq data of flavopiridol-treated HFF at particular positions for example clusters (indicated on the top left of subfigures). This includes all identified peaks for a gene not just the major peak used for identifying the TSS. For this purpose, the region ± 3 kb around the identified peak was divided into bins of 60 bp and for each bin the percentage of genes with a peak falling into this bin were calculated. The red dashed vertical line marks the identified TSS. Green dotted vertical lines indicate peak positions in mock infection and blue dotted vertical lines peak positions in WT-F infection at 3 h p.i. **(b,d,f)** Percentage of genes exhibiting an annotated TSS in each 60 bp bin around the identified TSS for example clusters (indicated on the top left of subfigures). TSS and peak positions in WT-F infection at 3 h p.i. are indicated as in **(a,c,e)**

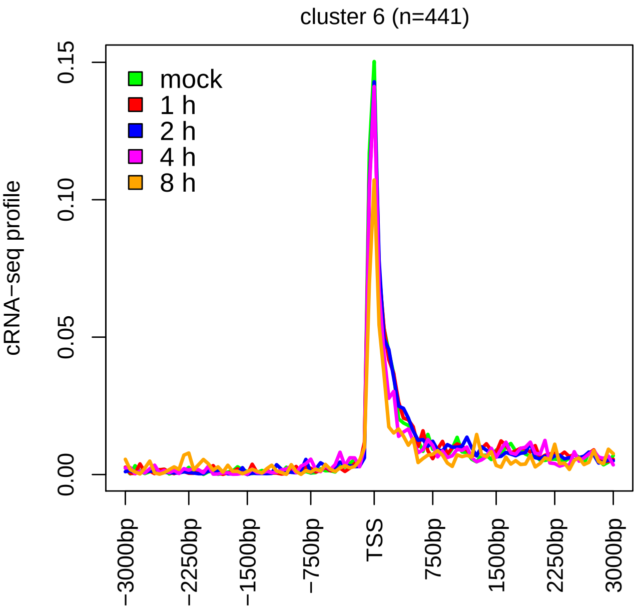

(a)

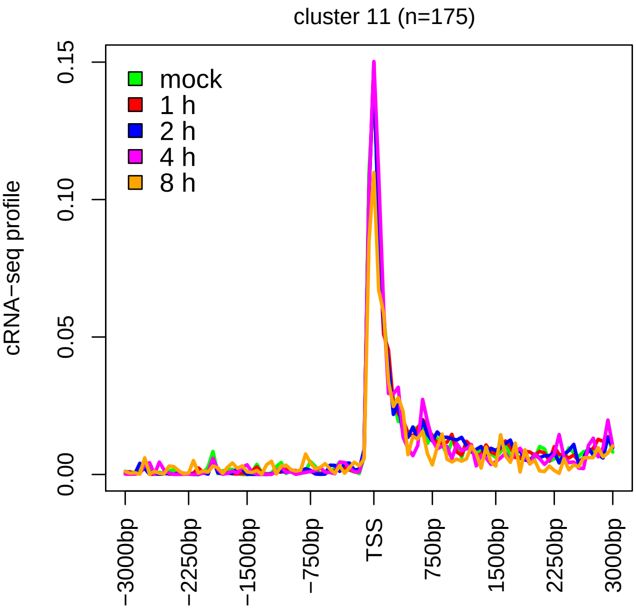

(b)

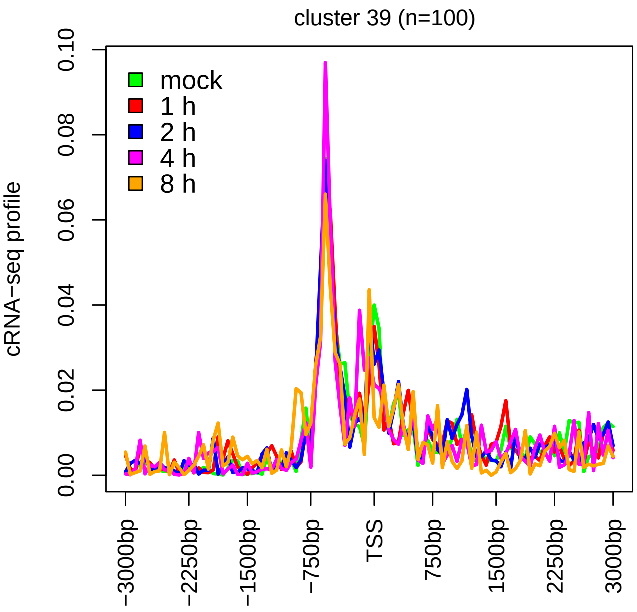

(c)

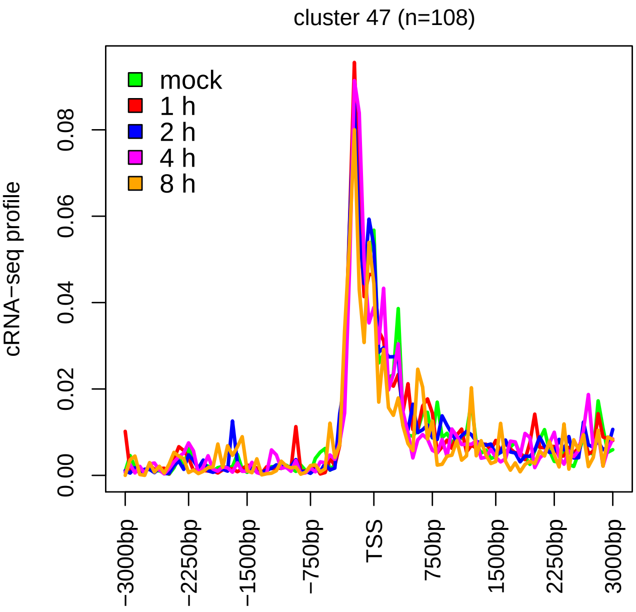

(d)

Fig. S11 Metagene plots of cRNA-seq profiles on the sense strand in mock and WT-17 infection at 1, 2, 4, and 8 h p.i. for example Clusters 6, 11, 39, and 47, which show broadening of peaks or additional peaks originating or increasing in height in PRO-seq data during WT-F infection. For metagene plots of PRO-seq profiles for these clusters see Fig. 2 and Fig. S3.

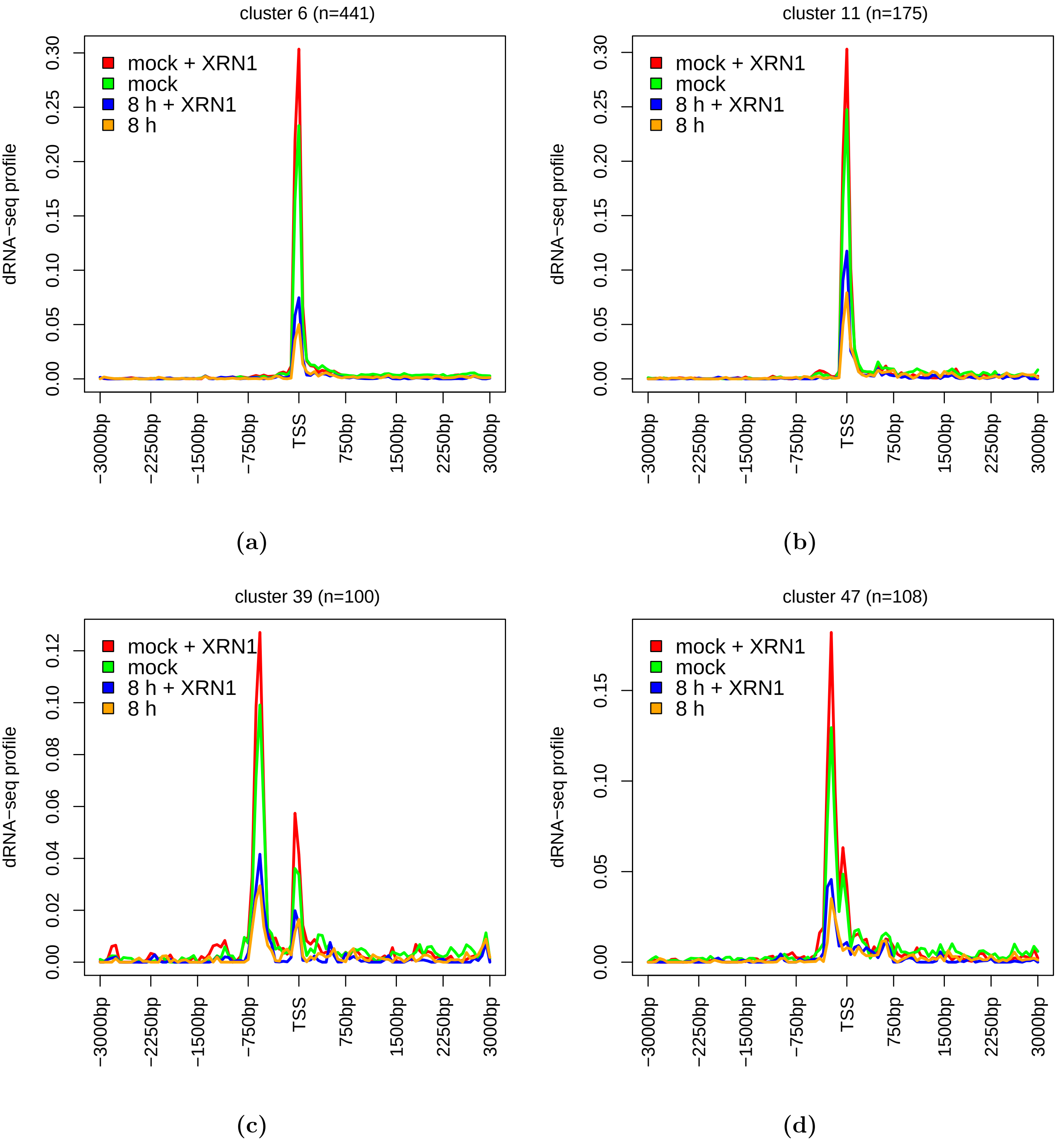

Fig. S12 Metagene plots of dRNA-seq profiles on the sense strand in mock and WT-17 8 h p.i. infection with and without XRN1 treatment for example Clusters 6, 11, 39, and 47, which show broadening of peaks or additional peaks originating or increasing in height in PRO-seq data during WT-F infection. For metagene plots of PRO-seq profiles for these clusters see Fig. 2 and Fig. S3.

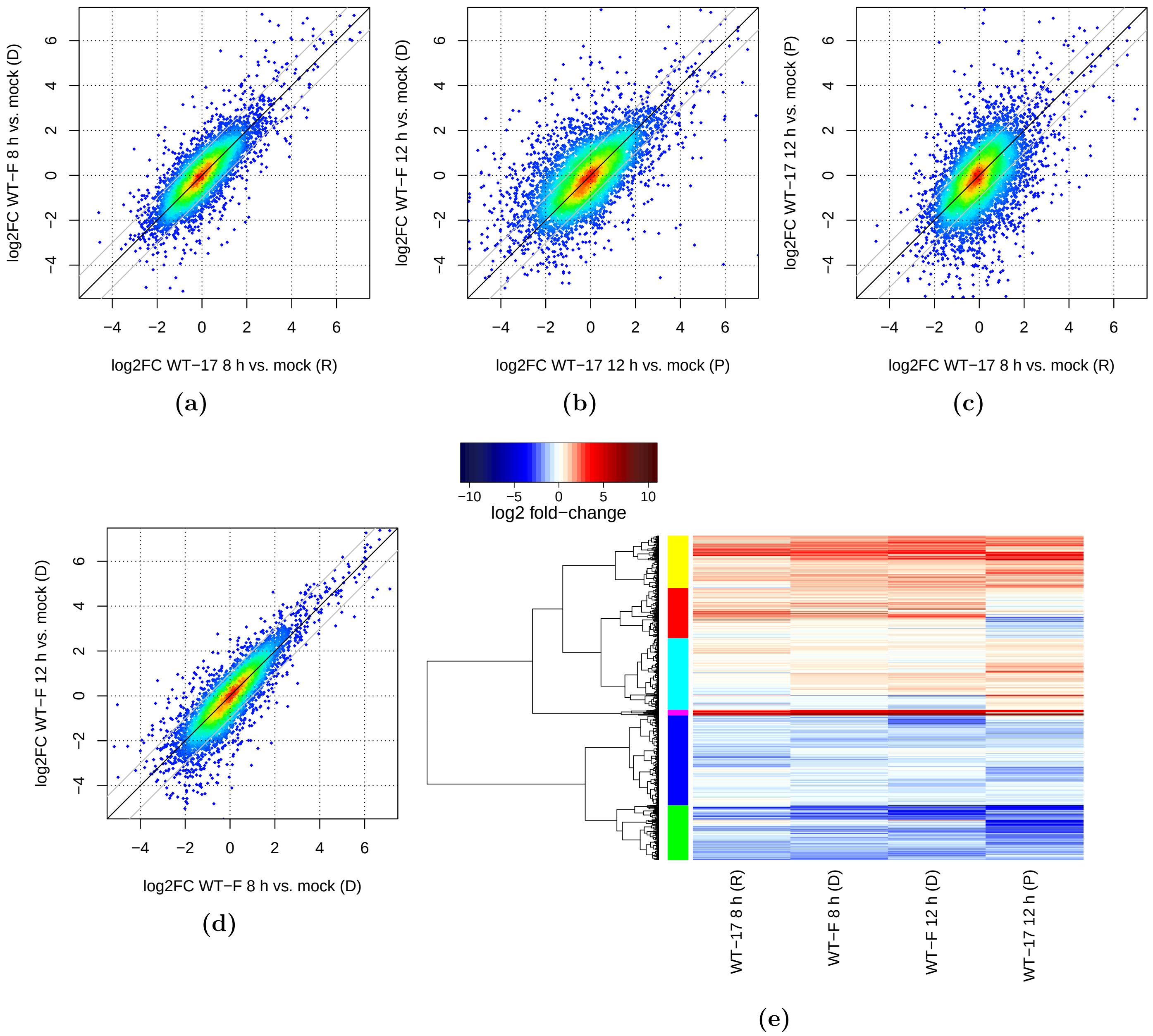

Fig. S13 (a-d) Scatter plots comparing log2 fold-changes (log2FC) in gene expression for analyzed genes between mock and WT-F or WT-17 infection at 8 or 12 h p.i. from the studies of Rutkowski *et al.* (WT-17 8 h p.i., R), Djakovic *et al.* (WT-F 8 and 12 h p.i., D) and Pheasant *et al.* (WT-17 12 h p.i., P). Colors indicate density of points from low (blue) to high (red). Black lines indicate the diagonal and gray lines a fold-change of 2. (e) Heatmap showing log2 fold-changes for WT-F or WT-17 infection at 8 or 12 h p.i. compared to mock for all genes differentially expressed (multiple testing adjusted p-value < 0.01) in at least one virus strain or time-point of infection. Hierarchical clustering was performed in R using Euclidean distances and Ward's clustering criterion. Six broad clusters were identified and are marked by colored rectangles on the right.

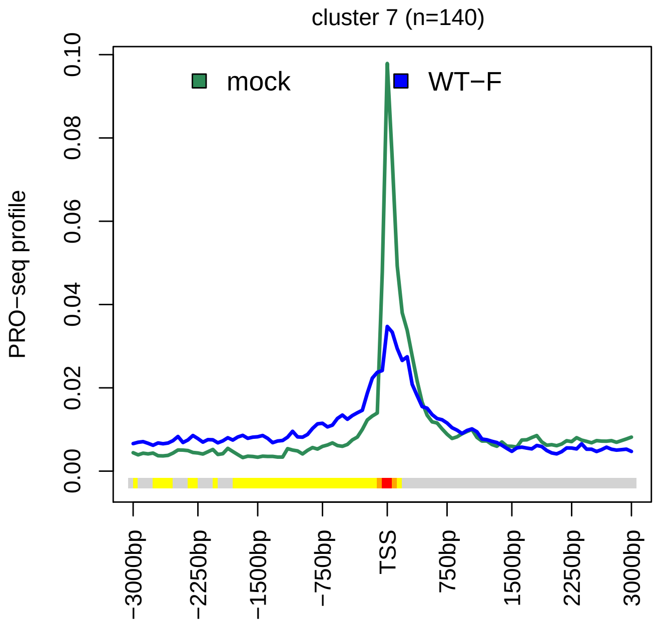

(a)

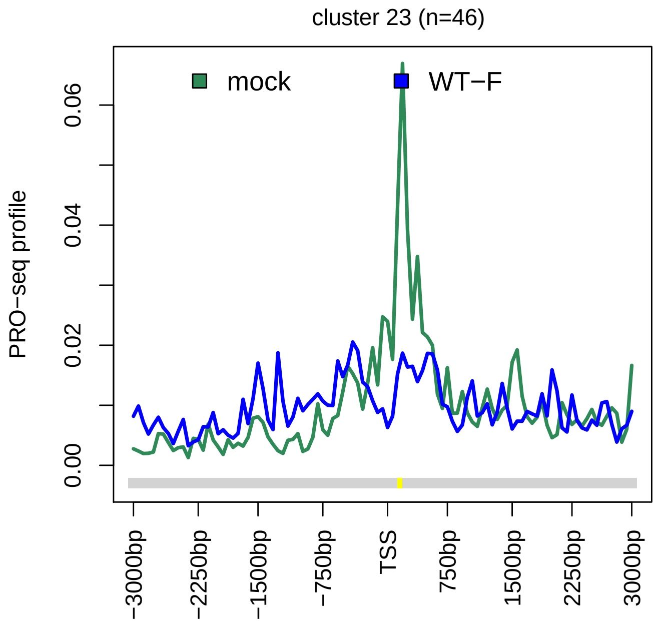

(b)

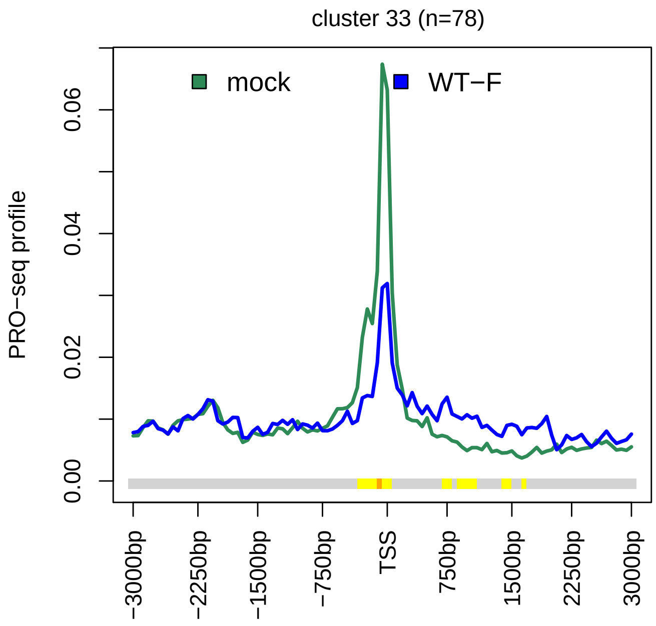

(c)

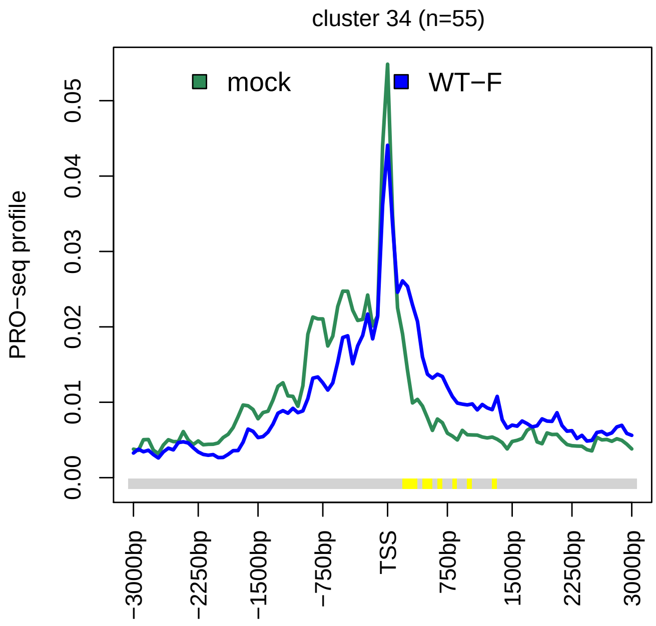

(d)

(Continued on next page)

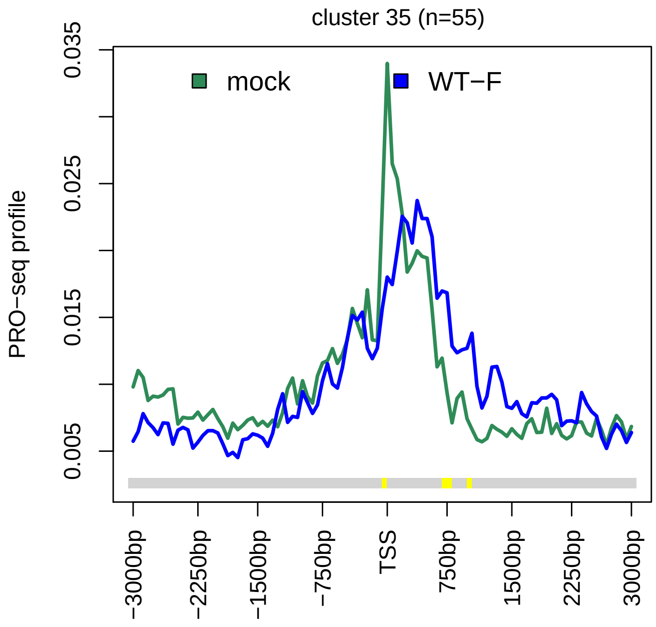

(e)

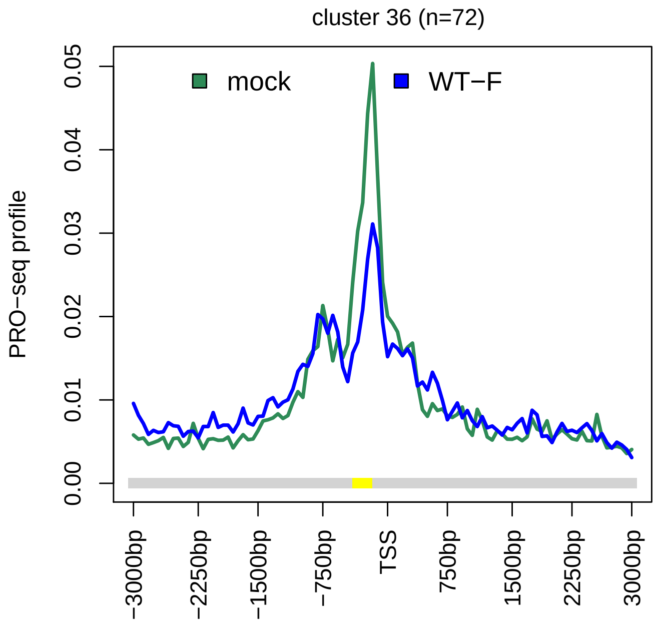

(f)

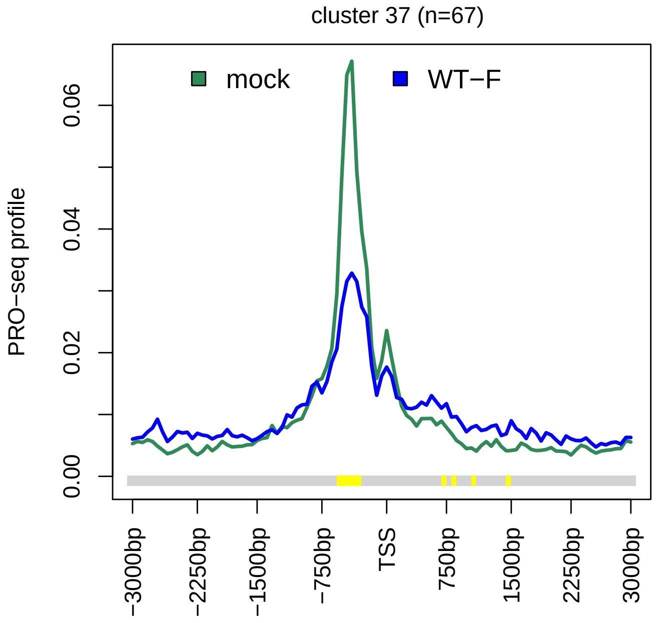

(g)

Fig. S14 Metagene plots showing the PRO-seq profile in sense direction from -3 kb to +3 kb around the TSS for mock infection (dark green) and WT-F 3 h p.i. infection (dark blue) separately for Clusters 7, 23, and 33 to 37, which exhibit a small extent of read-in transcription in 3-4 h p.i. 4sU-seq (see Fig. 5). Cluster numbers and number of genes in each cluster are indicated on top of subfigures. The color track at the bottom of each subfigure indicates the significance of paired Wilcoxon tests comparing the normalized PRO-seq coverages of genes for each bin between mock and WT-F 3 h p.i. infection. P-values are adjusted for multiple testing with the Bonferroni method within each subfigure; color code: red = adj. p-value $\leq 10^{-15}$, orange = adj. p-value $\leq 10^{-10}$, yellow = adj. p-value $\leq 10^{-3}$.

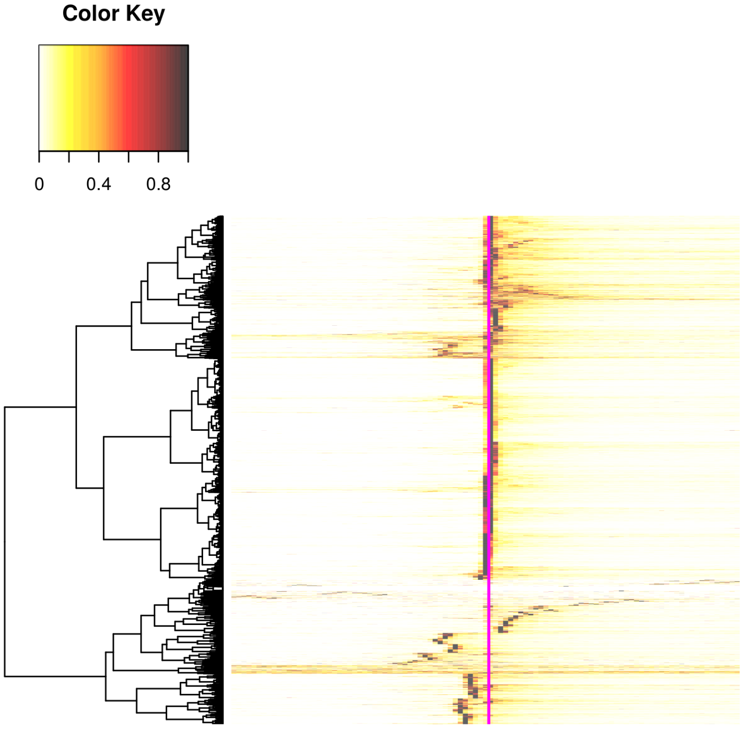

(a)

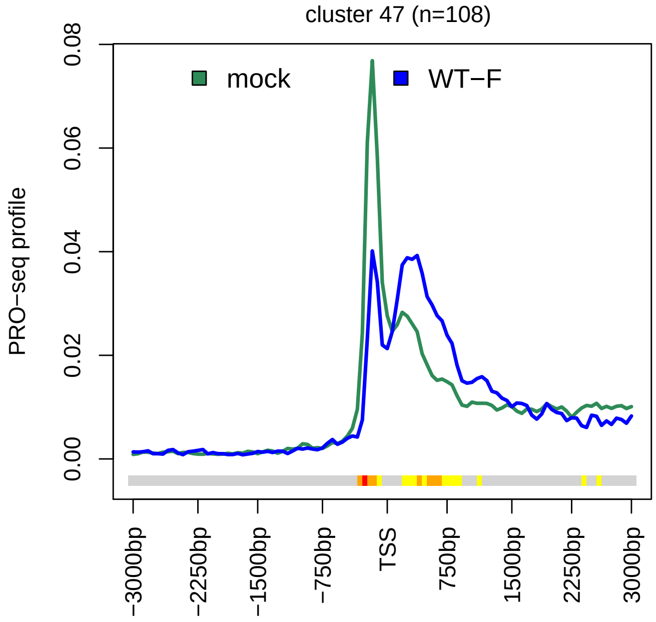

(b)

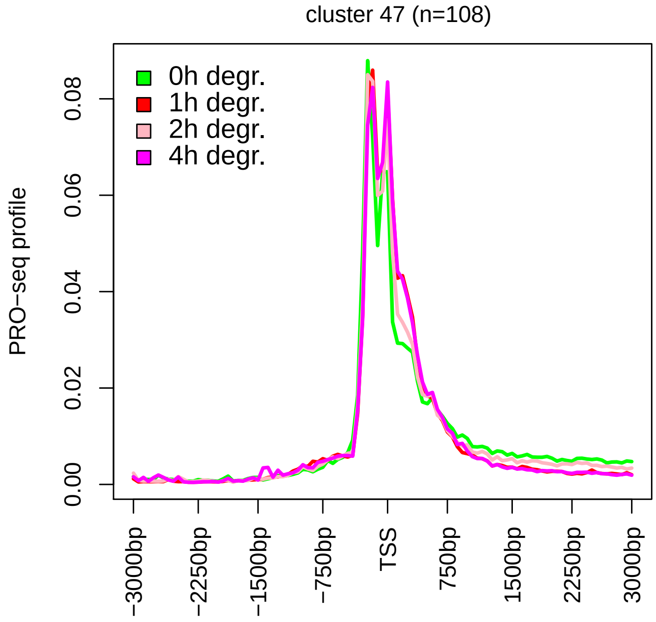

(c)

(Continued on next page)

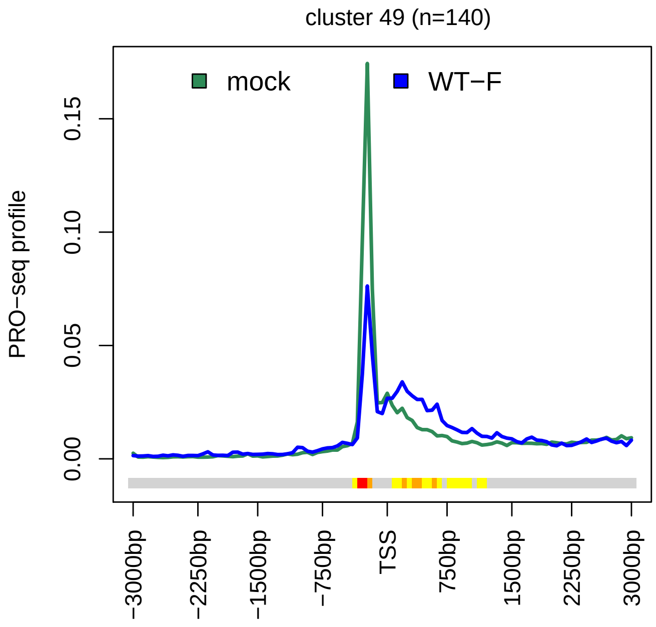

(d)

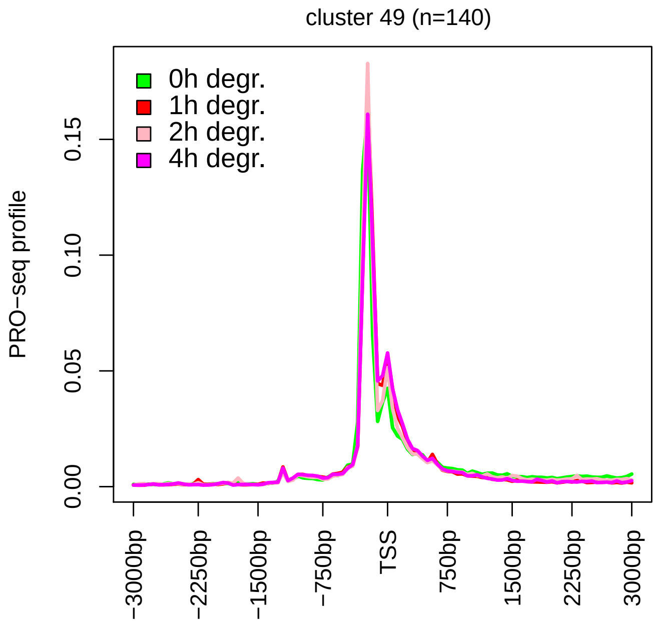

(e)

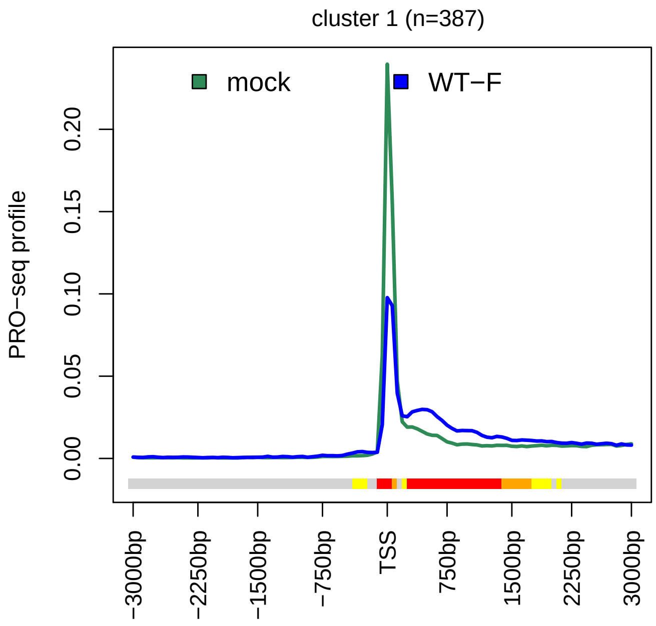

(f)

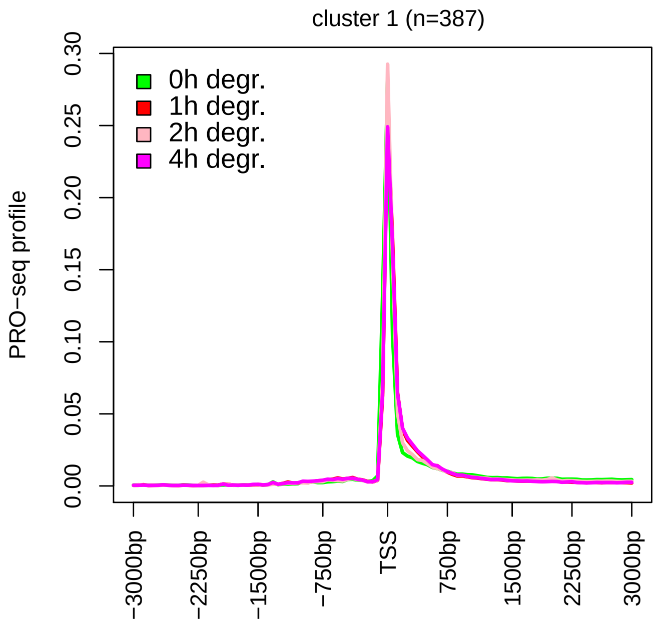

(g)

(Continued on next page)

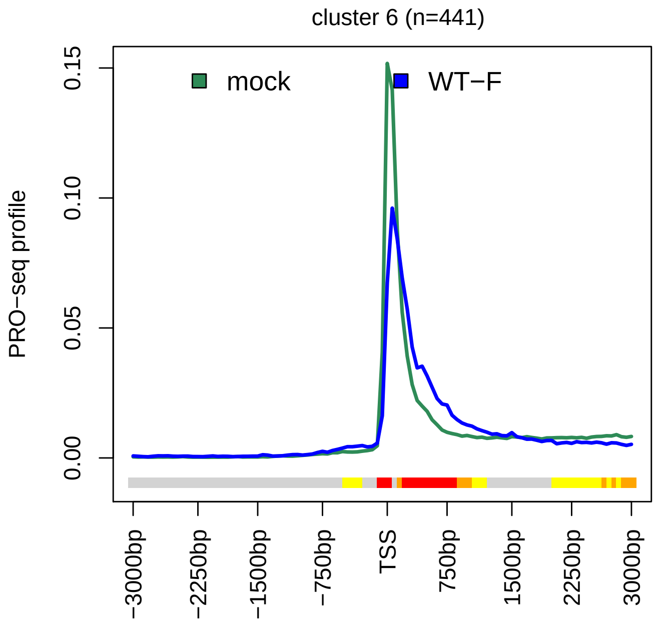

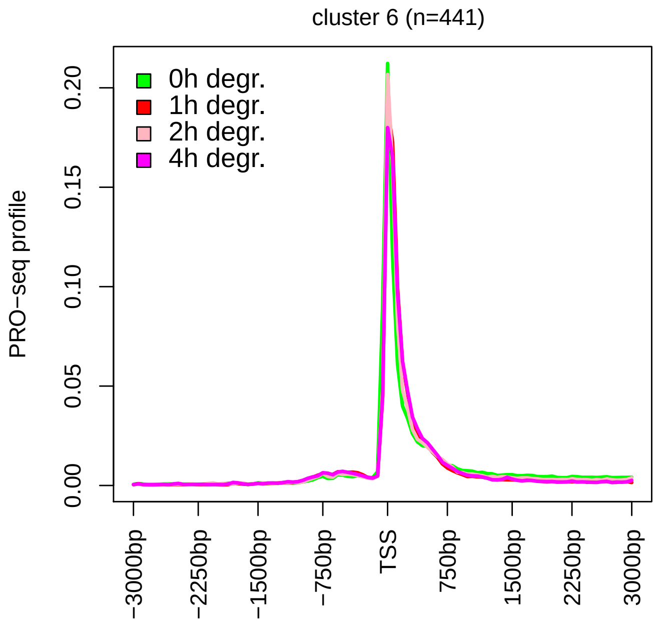

(h)

(i)

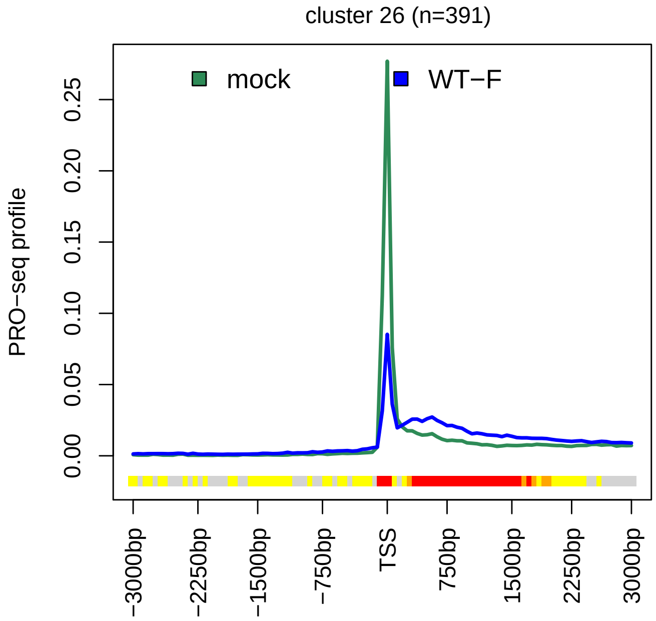

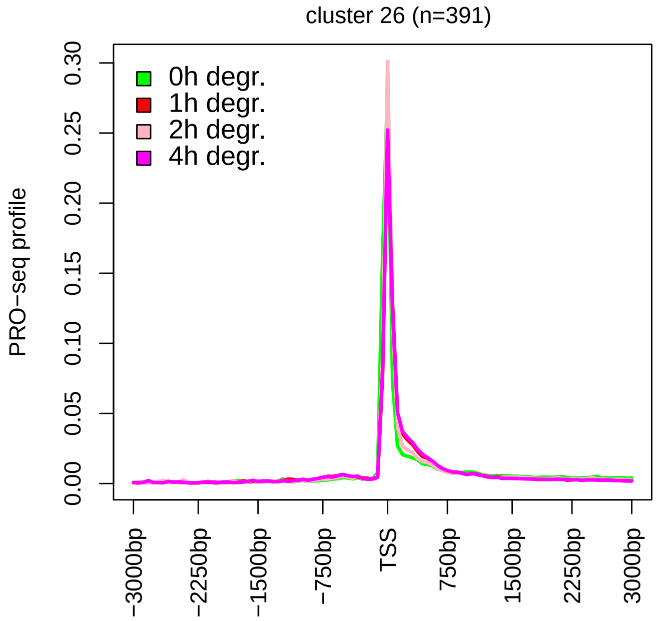

(j)

(k)

Fig. S15 **(a)** Heatmaps of PRO-seq profiles for 0 h auxin-inducible degradation of NELF from the study by Aoi *et al.* in a window of ±3 kb around the TSS positions identified from PROcap-seq and PRO-seq data of flavopiridol-treated HFF. For this purpose, PRO-seq profiles were divided by the maximum value in the ±3 kb promoter window, resulting in a value of 1 for the position of the highest peak in PRO-seq profiles. Hierarchical clustering of normalized PRO-seq profiles for all genes was performed using the *hclust* function in R according to Euclidean distances and Ward's clustering criterion. The central position in the promoter window (= the TSS identified in flavopiridol-treated HFF) is marked by a vertical magenta line. **(b-k)** Metagene plots around the TSS of PRO-Seq profiles for mock and WT-F 3 h p.i. infection from the study of Birkenheuer *et al.* (left column) and 0, 1, 2, and 4 h auxin-inducible degradation of NELF from the study by Aoi *et al.* (right column) for example clusters showing **(b-e)** an increased downstream peak or **(f-k)** only a reduced and slightly broadened TSS peak upon NELF degradation.

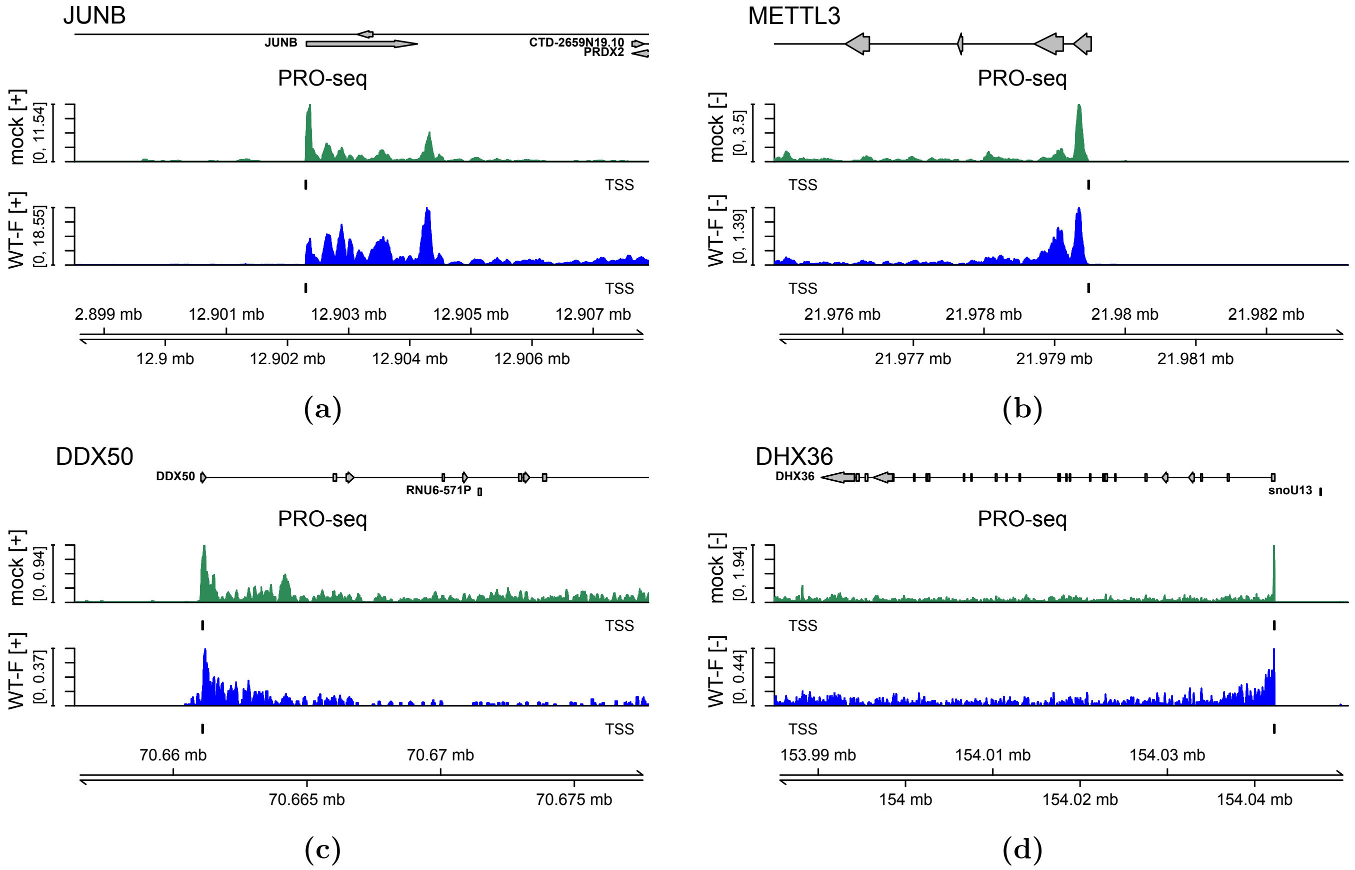

Fig. S16 Read coverage around the TSS in PRO-Seq data (sense strand only) for mock (green) and WT-F infection (blue) at 3 h p.i. for example host genes (gene name of the selected gene on the top left) mentioned in the discussion. Read coverage was normalized to total number of mapped reads and averaged between replicates. The identified TSS used in the analysis is indicated by a short vertical line below each read coverage track. Gene annotation is indicated at the top. Boxes represent exons, lines represent introns and direction is indicated by arrowheads. Genomic coordinates are shown on the bottom. Please note that figures are not centered around the TSS, but a larger region downstream of the TSS was included than upstream of the TSS.
